# Evolutionary history and patterns of geographical variation, fertility, and hybridization in *Stuckenia* (Potamogetonaceae)

**DOI:** 10.3389/fpls.2022.1042517

**Published:** 2022-11-03

**Authors:** Judith Fehrer, Michaela Nagy Nejedlá, C. Barre Hellquist, Alexander A. Bobrov, Zdenek Kaplan

**Affiliations:** ^1^ Institute of Botany, Czech Academy of Sciences, Průhonice, Czechia; ^2^ Department of Biology, Massachusetts College of Liberal Arts, North Adams, MA, United States; ^3^ Papanin Institute for Biology of Inland Waters, Russian Academy of Sciences, Borok, Russia; ^4^ Tyumen State University, AquaBioSafe, Tyumen, Russia; ^5^ Department of Botany, Faculty of Science, Charles University, Prague, Czechia

**Keywords:** geographic distribution, *Groenlandia*, hybridization, intraspecific variation, multigene phylogeny, Potamogetonaceae, species delimitation, *Stuckenia*

## Abstract

Aquatic plant species are often widespread, even across continents. They pose a challenge to species delimitation and taxonomy due to their reduced morphology and high phenotypic plasticity. These difficulties are even more pronounced in the case of interspecific hybridization. We investigate the aquatic plant genus *Stuckenia* for the first time on a worldwide scale. Expert species determination is aided by sequencing of nuclear ribosomal *ITS* and *5S-NTS* regions and the plastid intergenic spacers *rpl20-5’rps12* and *trnT–trnL*. Nuclear markers are used to infer hybridization, and the maternal origin of hybrids is addressed with plastid markers. Pure species are subjected to phylogenetic analyses. Two main *Stuckenia* lineages are found: one consists of *S. amblyphylla*, *S. filiformis*, *S. pamirica*, and *S. vaginata*, the other includes *S. pectinata* and *S. striata.* The widespread species *S. pectinata*, *S. filiformis*, and *S. vaginata* show intraspecific genetic variation, which is structured geographically. Many intraspecific hybrids, which are usually fertile, occur between those genotypes. Interspecific hybrids, which are consistently sterile, are detected among all widespread species; some are reported for the first time in several countries and regions. They originated multiple times from reciprocal crosses and reflect the geographical origins of parental genotypes. Intraspecific genetic variation can be higher than interspecific differences between closely related species. Comparison of phenotypic variation in the field and in cultivation with genotypic variation shows that numerous conspicuous forms have been overestimated taxonomically. These are resolved as phenotypes responding to unusual environments, have recurrently evolved adaptations, or represent extreme forms of continuous variation of the recognized species. However, some specific regional lineages, which have evolved from variable species, may be interpreted as early steps of the speciation process. Hybridization has been underestimated in some regions as a source of *Stuckenia* diversity, and the respective hybrid plants have been misidentified as intraspecific taxa or even as separate species. Many erroneous entries in sequence databases are detected and summarized. This work provides a sound basis for species delimitation and hybrid recognition in this difficult genus.

## Introduction

Taxonomic revisions are essential for a fundamental understanding of biodiversity. The most appropriate way is to revise the entire existing diversity of the taxon in question, usually a genus or a family. Many taxa in these ranks have large geographical ranges, and dealing with all specimens from their entire ranges is technically difficult. Most taxonomic revisions are therefore produced for rather small areas, such as a country, often within the framework of producing a national flora. However, these studies inevitably deal with only a part of the total taxonomic diversity and unrepresentative sampling of variation, ignoring some information relevant to adequate taxonomic decisions and conclusions. This often leads to incompatible taxonomic treatments in different geographical parts of the taxon’s range. The consequences include different numbers of recognized species (and other taxa), different delimitations of included taxa, and misapplied names of taxa first described from distant areas. It is, therefore, necessary to study material from the entire range of the respective taxon.

Potamogetonaceae is one of the most diverse and taxonomically difficult families of aquatic plants (Wiegleb and Kaplan, 1998). The main sources of taxonomic complexity include their reduced morphology, which limits the number of taxonomic characters that can be used to separate species (Preston and Croft, 1997; Kaplan and Štěpánek, 2003; Kaplan et al., 2009); extensive phenotypic plasticity (Kaplan, 2002; Kaplan and Zalewska-Gałosz, 2004; Kaplan, 2005; Kaplan, 2008), partitioning of genetic variation between rather than within populations (Hettiarachchi and Triest, 1991; Kaplan and Štěpánek, 2003) and the occurrence of numerous hybrids (e.g., Preston, 1995; Wiegleb and Kaplan, 1998; Kaplan and Fehrer, 2007; Kaplan et al., 2009; Kaplan, 2010a). In the taxonomic concept adopted here (see Aykurt et al., 2020 for discussion), the Potamogetonaceae includes about 80 species and 105 hybrids classified into three genera, namely *Potamogeton* with about 72 species and 99 hybrids, monotypic *Groenlandia*, and *Stuckenia* with seven species and six hybrids (Kaplan, 2008; Kaplan, 2010a; Kaplan et al., 2013). *Stuckenia* includes only hexaploids, whereas *Groenlandia* is diploid, and *Potamogeton* includes mainly diploids and tetraploids (Kaplan et al., 2013).

The group of *Potamogeton* s. l. encompassing species that now belong to *Stuckenia*, was first distinguished already in the first half of the 19th century (for a review, see Kaplan, 2008). However, the genus *Stuckenia* has been widely recognized only in the past few decades. It includes seven species: the cosmopolitan *S. pectinata* (L.) Börner; *S. filiformis* (Pers.) Börner, distributed in the Northern Hemisphere and South America; *S. vaginata* (Turcz.) Holub with a markedly disjunct range consisting of three main areas (Scandinavia, southern Siberia, and North America) and several isolated outposts; *S. amblyphylla* (C. A. Mey.) Holub, distributed in western and Central Asia; *S. macrocarpa* (Dobrochot.) Tzvelev and *S. pamirica* (Baagöe) Z. Kaplan, both endemic to Central Asia; and *S. striata* (Ruiz & Pav.) Holub, distributed in North and South America. The center of diversity is in the mountains of Central Asia and the adjacent lowlands of Siberia and Kazakhstan, where six of the seven species occur (Kaplan, 2008). While fruiting, well-developed specimens can usually be identified by an experienced aquatic botanist, the identity of sterile, underdeveloped, and running-water forms is sometimes obscure, and the identification process is even more complicated by the occasional occurrence of hybrids. Three interspecific hybrids are recognized: *S.* ×*bottnica* (Hagstr.) Holub (= *S. pectinata × S. vaginata*), *S.* ×*fennica* (Hagstr.) Holub (= *S. filiformis × S. vaginata*), and *S.* ×*suecica* (K. Richt.) Holub (= *S. filiformis × S. pectinata*). All of these were discovered and documented based on morphological intermediacy associated with observations of sterility, but treated within the genus *Potamogeton* (e.g., Hagström, 1916; Dandy, 1975; Preston, 1995). So far, molecular methods have been insufficiently applied to investigate genetic variation patterns in *Stuckenia*, and the respective studies have either focused on hybridization (Preston et al., 1998; King et al., 2001; McMullan et al., 2011; Du and Wang, 2016), or have been geographically and taxonomically very limited (e.g., Lindqvist et al., 2006; Wang et al., 2007; Zhang et al., 2008; Volkova et al., 2017). No thorough global study on species relationships and delimitation in *Stuckenia* based on molecular data has been published.

To fill this gap, we sampled *Stuckenia* from all over the world and applied four highly variable molecular markers: the nuclear ribosomal internal transcribed spacer (*ITS*) and the 5S non-transcribed spacer (*5S-NTS*) regions (two unlinked multicopy markers) and the plastid *rpl20-5’rps12* and *trnT-trnL* intergenic spacers, which we applied to (i) inform about species delimitation, (ii) reconstruct the phylogeny of *Stuckenia*, (iii) detect hybrids, and (iv) determine the maternal parent of hybrids. In the sister genus *Potamogeton*, like in most angiosperms, plastid DNA is inherited maternally (Kaplan and Fehrer, 2006), and we, therefore, assume that this is also the case with *Stuckenia*. All these markers have proved suitable to resolve species relationships in *Potamogeton* (e.g., Iida et al., 2004; Lindqvist et al., 2006; Kaplan and Fehrer, 2011; Kaplan et al., 2013; Ito et al., 2016; Kaplan et al., 2018; Fehrer et al., 2022), and some of them have also been applied in *Stuckenia* (Iida et al., 2004; Lindqvist et al., 2006; Wang et al., 2007; Zhang et al., 2008; Du and Wang, 2016; Kuzmina et al., 2017; Volkova et al., 2017).

## Materials and methods

### Plant material for molecular analyses

All accessible species and as many populations of *Stuckenia* as possible were obtained in a worldwide sampling effort from 1996 to 2014. This effort covered as much of the geographic distribution range of individual species as possible. Aside from the ordinary forms of pure species, we intentionally collected hybrids and aberrant phenotypes of unclear identity. This is to cover as much of the existing diversity within the genus as possible. Three samples of *Groenlandia densa* (L.) Fourr., a monotypic sister genus of *Stuckenia*, were included as an outgroup. Additional samples were obtained from herbarium specimens (for a list of herbaria studied, see Kaplan, 2008; Kaplan, 2010a; Kaplan, 2010b). Altogether, the study includes 170 samples of almost the same number of populations from 23 countries; among them are large collections from Russia and the USA. The *ITS* region was sequenced for all samples; for the other markers, large and geographically representative subsets were sequenced (**Supplementary Table 1**). Material freshly collected for DNA isolation was preserved in CTAB solution or dried in silica gel. Voucher specimens were prepared from the same plants and preserved in the herbarium PRA (**Supplementary Table 1**).

### Cultivation experiments

Numerous samples, particularly vegetative plants from running water without fruits, plants morphologically intermediate between recognized species, and taxonomically unclear forms, were transferred to cultivation in the experimental garden at the Institute of Botany, Průhonice, Czech Republic, and used for tests of fertility and phenotypic plasticity. To correctly distinguish truly (genetically) sterile plants from vegetative phenotypes that do not produce reproductive organs due to unsuitable habitat conditions (such as a strong water current, deep water, or a lack of nutrients), the plants were grown in pots with standard pond mud in tanks with standing shallow water for at least 5 years, and the capacity to flower and set fruit was recorded.

### Molecular procedures

DNA was isolated following a sorbitol extraction protocol (Štorchová et al., 2000). The *ITS* region was amplified as described in Kaplan and Fehrer (2004); amplification of the *5S-NTS* was done as in Kaplan et al. (2013); *rpl20-5’rps12* amplification follows Kaplan and Fehrer (2006); the *trnT-trnL* spacer was amplified as described in Iida et al. (2004) and Kaplan et al. (2018). PCR products were purified using the QIAquick PCR purification kit (Qiagen, Hilden, Germany) and sequenced at GATC Biotech (Cologne, Germany)/Eurofins Genomics (Ebersberg, Germany). The PCR primers were employed for sequencing; *ITS*, *rpl20-5’rps12*, and *trnT-trnL* templates were usually sequenced in one direction only, in the case of difficult reads in both directions; the *5S-NTS* region was always sequenced in both directions because of the high number of intra-individual polymorphic sites and occasional length variation (Kaplan et al., 2018). Sequence electropherograms were edited manually using Chromas version 1.45 (Technelysium Pty Ltd., Australia) and aligned in Bioedit version 7.0.9.0 (Hall, 1999).

If required for readability and to determine the exact position and length of indels, individual samples of the *5S-NTS* marker were cloned as described in Fehrer et al. (2009) except that vector primers instead of PCR primers were used for re-amplification; several clones were sequenced with the M13 primer to obtain full length reads. Most hybrids in *Potamogeton* maintain *ITS* ribotypes of both parents (e.g., Kaplan and Fehrer, 2007; Kaplan et al., 2009; Kaplan and Fehrer, 2011; Kaplan et al., 2019); hybrid identification in *Stuckenia* is therefore based on the same marker. For samples that showed character additivity in direct sequences of the *ITS* region, in the case of strongly skewed ratios of parental copy types, minor and major sequences were determined by peak subtraction; otherwise, the sample was cloned. A minimum of five clones per sample were sequenced to retrieve both parental copies; if needed, additional clones were sequenced until the missing copy type was found. If only one parent was represented by several clones and the other parent’s sequences consisted only of recombinants, the second parent was also inferred by peak subtraction based on the direct sequence and the clones of the first parent. Recombinant clones were dismissed, and polymerase errors (unique substitutions in single clones that did not correspond to polymorphisms in the direct sequence) were corrected; one representative clone of each parent was chosen to represent the hybrid sample. If one of the parents was polymorphic, two clones representing both variants were chosen. In the case of several hybrids from the same population, if their direct sequences suggested they were identical, only one sample was cloned, and direct sequences of the other sample(s) were submitted with additive polymorphisms until the position of the first indel. All sequences were submitted to the GenBank database (accession numbers OP101176–OP101375, OP136177–OP136533). They are listed in **Supplementary Table 1**.

### Data treatment and selection of samples for tree construction

For all markers, the intraspecific variation was compiled (**Supplementary Tables 2** **–** **5**). Different genotypes within species were assigned; for the nuclear markers, the number of intra-individual polymorphic sites and indels was summarized.

**Figure 1:**
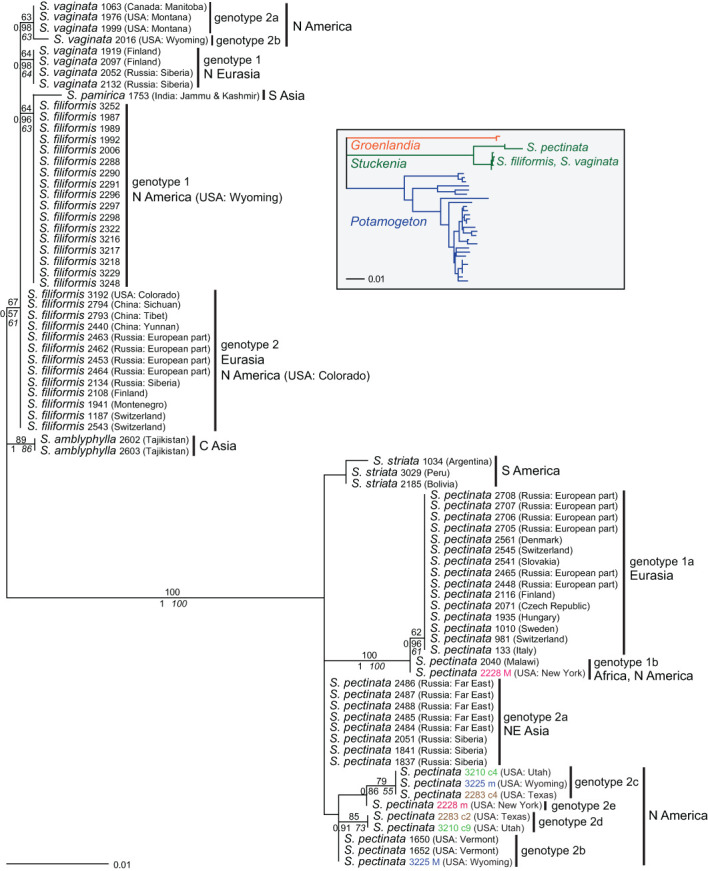
Phylogenetic analyses of *Stuckenia* based on the *ITS* region. The Maximum Likelihood tree with the highest log likelihood (−1,345.98) is shown with bootstrap values above branches. Posterior probabilities from Bayesian inference and bootstrap support of Maximum Parsimony analyses are below branches. Genotypes within species are indicated as well as the geographic origin of the samples. Four pairs of accessions are shown in color; matching colors belong to the same accession and represent different alleles or clones of intraspecific hybrids of *S. pectinata*. The inset shows a simplified tree from Kaplan et al. (2013) showing the relationships among Potamogetonaceae genera and the two main clades of *Stuckenia*.

***ITS***—Samples showing a combination of different genotypes within the same species (intraspecific hybrids) were excluded from phylogenetic analyses except for alleles or clones that represented unique genotypes (**Supplementary Table 2**). All sequences containing further polymorphisms, but otherwise identical to particular genotypes were excluded, except for one sample (*S. pectinata* 3225) that contained one polymorphic site but showed a unique combination of different genotypes. Samples of *S. striata* and *S. pamirica* were included to represent the species. However, all of them showed one to three polymorphisms, and most of them also had one indel polymorphism. A partial sequence (*S. pectinata* 2694) was excluded as well. Sequences of *Groenlandia* (and *Potamogeton*) could not be aligned unequivocally. Therefore, to focus on the intrageneric patterns of *Stuckenia*, no external outgroup was used. An *ITS* tree depicting the relationships at the genus level from Kaplan et al. (2013) is included in **Figure 1**.

Sequences of samples showing character additivity between species (interspecific hybrids) were aligned with a selection of all the genotypes found in their respective parents, and variable positions were summarized (**Supplementary Table 6**). Several genotypes of hybrids were not found in their parental species; these samples were subjected to phylogenetic analysis along with all representative genotypes of pure parental species.

***5S–NTS***—In *Potamogeton*, this marker is characterized by a large number of intra-individual polymorphic substitutions and indels that are species specific but not additive except in the case of hybrids (Kaplan et al., 2018). The same feature was found for *Stuckenia*. Sequences including all polymorphic sites and sequences showing only the dominant character states in both reading directions were aligned, and their intraspecific variation was assessed (**Supplementary Table 3**). One sample that showed character additivity (intraspecific hybrid) was excluded from phylogenetic analyses. Small additional peaks that did not constitute more than about 30% of the total signal in both reading directions and did not show indications of character additivity with other species were ignored, and only the major sequences were used for tree construction. Polymorphic, as well as major sequences, were submitted to GenBank, but only the major sequences were used for tree construction. *Groenlandia densa* was too divergent to be aligned with sequences of *Stuckenia* with this marker. Each of the three samples analyzed included pairs of highly divergent sequences that were even difficult to align to each other; clones of these samples were analyzed separately.

***Rpl20–5’rps12***—Based on the intraspecific variation (**Supplementary Table 4**), 82 identical samples were excluded from phylogenetic analyses and mapped afterwards on the tree. Sequences of hybrids were included to show their maternal origins; the identification of hybrids was based on *ITS*. *G. densa* was alignable with this marker and was included as an outgroup.

***TrnT-trnL***—According to the intraspecific variation observed with this marker (**Supplementary Table 5**), only a few identical sequences were included and the rest (40 samples) were mapped later on the tree. Hybrids between *S. pectinata* and *S. filiformis* or *S. vaginata* were not sequenced for this marker; their maternal parent was already unequivocally determined by *rpl*20–5’*rps*12. Some hybrids between the closely related species *S. vaginata* and *S. filiformis* as well as all hybrids whose identification remained equivocal with *rpl*20–5’*rps*12 were included. *G. densa* was too divergent with this marker to be aligned unambiguously and was excluded as an outgroup.

***Combined dataset***—Sequences of samples for which all four markers were available were concatenated; all intra- and interspecific hybrids and some further sequences showing partial additivity between different genotypes of the same species in the *5S-NTS* were excluded. For one sample (2705), the *rpl*20–5’*rps*12 region did not amplify, missing data for this region were specified as N.

### Phylogenetic analyses

All datasets contained indels that were parsimony informative. They were coded as additional characters using FastGap version 1.2 (Borchsenius, 2009), based on the simple method of Simmons and Ochoterena (2000). Maximum Parsimony (MP) analyses were performed with PAUP version 4.0b10 (Swofford, 2002); Maximum Likelihood (ML) analyses were done using MEGA X version 10.0.05 (Kumar et al., 2018); Bayesian inference (BI) was computed with MrBayes version 3.2.2 (Ronquist et al., 2012). MP analyses were computed as heuristic searches with 100 random addition sequence replicates and TBR branch swapping. These searches saved no more than 100 trees with a length greater than or equal to 1 per replicate. Bootstrapping was done with the same settings and 1,000 replicates. For ML and BI, the model best fitting the presumed molecular evolution of the respective datasets was determined in Hierarchical Likelihood Ratio Tests (hLRTs) with Modeltest version 3.5 (Posada and Crandall, 1998). If the particular model was not implemented in MEGA, a similar one was used. For ML analyses, the matrix of coded indels (A, C, N) was concatenated to the respective alignments, and extensive subtree pruning–regrafting, a very strong branch swap filter, and 1,000 bootstrap replicates were used. For BI, the basic model parameters, i.e., the distribution of rates among sites and the number of different substitution rates were set as priors; apart from that, the default settings were used. Chains were computed for a variable number of generations (see below), sampling every 1,000th tree, until all indicators (average standard deviation of split frequencies <0.01, potential scale reduction factors around 1) suggested that convergence between the different runs was achieved for each dataset. The first 25% of the trees per run were discarded as burn-in and the remaining trees were summarized.

***ITS*** *—*The resulting dataset of pure species consisted of 78 samples and 734 characters, including eight coded indels; five variable characters were uninformative, and 41 characters were parsimony informative. A JC model was found to best represent the molecular evolution of this marker within *Stuckenia*; it was used to compute ML and BI trees. One million generations were needed to reach convergence in BI. The dataset including hybrid accessions that differed from previously identified genotypes of parental species consisted of 31 taxa and 692 characters, including seven coded indels; seven variable characters were uninformative, and 44 characters were parsimony informative. The same model and settings were used for the analysis of the parental species.

***5S–NTS***—The dataset consisted of 53 samples of ‘pure’ species of *Stuckenia* and 284 aligned characters, including four coded indels; 30 variable characters were uninformative, and 71 characters were parsimony informative. A JC + Γ model was found in hLRTs to best represent molecular evolution. For BI, one substitution rate and gamma distribution among sites were set as priors; 1.2 million generations were needed to reach convergence. For ML, a JC model with gamma distribution (two categories) was applied. For *G. densa*, 26 clones of three samples were, along with one sequence available from GenBank (DQ786446), subjected to Neighbor Net analyses using Splitstree version 4.14.6 (Huson and Bryant, 2006).

***Rpl20–5’rps12***—The dataset included 41 samples of species and hybrids and 807 characters, of which four variable characters were uninformative and 46 were parsimony informative. A K81uf + Γ model was found to best match the presumed molecular evolution. Six substitution rates and a gamma distribution were used as priors for BI; 1.5 million generations were required for runs to converge. The Kimura-3-parameter model is not implemented in MEGA and was replaced by a Tamura-3-parameter model. A gamma distribution was used to model evolutionary rate differences among sites (two categories).

***TrnT-trnL***—The dataset consisted of 40 samples of species and hybrids and 856 characters, of which eight were coded indels; 13 variable characters were uninformative and 31 were parsimony informative. An F81 + Γ model was found in hLRTs to best represent the molecular evolution. For BI, one substitution rate and gamma distribution among sites were set as priors; one million generations were needed to reach convergence. The Felsenstein 81 model is not implemented in MEGA, therefore a JC model with gamma distribution (two categories) was applied.

***Combined dataset***—The concatenated dataset consisted of 35 samples and 2,599 characters, including 21 coded indels; 45 variable characters were uninformative, and 143 characters were parsimony informative. For hLRTs, an F81 + Γ model was found to best represent the molecular evolution; for ML, it was replaced by a JC model with gamma distribution (two categories). BI needed one million generations to converge.

### Comparison with sequences from other sources

To assess the degree of similarity of *Stuckenia* samples from other sources with our own collection, all sequences of *Stuckenia* available for the markers used in this study were retrieved from GenBank. In addition, BLAST searches were performed using representative genotypes of this study as queries but excluding *Stuckenia* as a taxon in order to identify samples belonging to other genera, which might have been erroneously attributed to *Stuckenia*. For *ITS*, all sequences of other genera with at least 95% similarity to any of our samples were retained (other genera differ by more than 10% from *Stuckenia*). Three sequences of *S. macrocarpa* × *S. pectinata* (MH427619, MH427621, and MH427637) and two sequences of intraspecific hybrids of *S. pectinata* (KY407954 and MH427640) containing additive polymorphisms were excluded from phylogenetic analyses. For *5S-NTS*, the divergence to other genera was so large that BLAST searches for highly similar sequences did not retrieve any matches except for one sample that was wrongly attributed to *Stuckenia*. For *trn*T–*trn*L, one sequence (EF471051) from Zhang et al. (2008) attributed to *S. filiformis* differed strongly from all other samples of *Stuckenia*. A BLAST search revealed that it was most similar to several closely related species of *Potamogeton*; these were retrieved and analyzed separately along with the sample in question. The other *trn*T–*trn*L sequences from GenBank were analyzed along with our material.

All samples retrieved from GenBank were aligned with representatives of all genotypes and species in our collection. If only partial sequences were available, missing positions were replaced by Ns. Several *trn*T–*trn*L sequences from other sources contained obvious reading errors at their beginning or end, so the alignment was truncated prior to analysis in order not to overestimate the haplotype variation. Indel coding was done as described previously, and neighbor joining trees were produced with PAUP version 4.0b10 (Swofford, 2002) for all datasets (**Supplementary Figures 1** **–** **4**). Obviously, or most likely, misidentified sequences retrieved from GenBank were summarized.

## Results

### *ITS* sequence features and intraspecific variation

Sequences of the *ITS* region were well homogenized in ‘pure’ species although the entire genus consists only of hexaploids; 73% of the sequences did not contain a single polymorphic site, a further 10% only one, and 17% contained two to three polymorphisms; among the latter were four samples that also included one indel polymorphism (**Supplementary Table 2**). Different genotypes were found within the three most widespread species for which broad sampling was available: *S. pectinata*, *S. filiformis*, and *S. vaginata*.

Within *S. pectinata*, two major genotypes (1 and 2) were found, differing by six substitutions. Genotype 1 had two variants (1a and 1b); one sample from Africa differed by a single substitution from Eurasian samples. Genotype 2 had five subtypes (2a–2e); one comprised samples from NE Asia, and the other four were from N America (**Figure 1**). Intraspecific ‘hybrids’ occurred among these genotypes, indicated by character additivity at the respective positions (**Supplementary Table 2**). Intraspecific hybrids between genotypes 1a (Eurasia) and 1b (Africa) were found in Central Europe (Switzerland, Slovakia, Denmark) and India (**Supplementary Table 1**). Several American accessions were composed of different combinations of subtypes of genotype 2 (**Figure 1**, **Supplementary Table 7**). Samples combining variants of genotypes 1 and 2 were from China, India, the USA, and Finland; the Indian sample contained the African subtype 1b. Among 61 samples of *S. pectinata*, 19 were intraspecific hybrids with various combinations of genotypes.

Within *S. filiformis*, two genotypes occurred that differed by a single substitution; they mainly corresponded to Eurasian or American samples, respectively. A single American sample from Colorado belonged to the Eurasian genotype (**Figure 1**), and three further American samples from Maine and Wyoming showed a mixture of both genotypes. Altogether, three out of 35 samples of *S. filiformis* showed a polymorphic site composed of both genotypes, all of them from North America.

Within *S. vaginata*, two main genotypes (1 and 2) were found that differed by two substitutions; genotype 2b was derived from 2a (one additional substitution; **Supplementary Table 2**). Samples with genotype 1 originated in Northern Eurasia; those with genotype 2 originated from North America. No intraspecific hybrids were found among the eight samples analyzed.

### Species relationships of *Stuckenia* based on *ITS*

The species fell into two main groups; one comprised *S. pectinata* and *S. striata*; the other consisted of *S. filiformis*, *S. vaginata*, *S. amblyphylla*, and *S. pamirica* (**Figure 1**). The American species *S. striata* was very similar to the North American genotype 2 of *S. pectinata*, even more similar than the different genotypes of *S. pectinata* were to each other. The Indian sample of *S. pamirica* appeared to be derived from the American genotype of *S. filiformis*. Samples of *S. amblyphylla* from Central Asia formed a well-supported branch; their sequence differed from that of *S. filiformis* by three to four substitutions (**Supplementary Table 2**). Relationships between *S. filiformis* and *S. vaginata* were unresolved; different intraspecific genotypes of each species were as divergent from each other as interspecific differences.

### Interspecific hybridization in *Stuckenia* inferred from the *ITS* region

**Figure 2:**
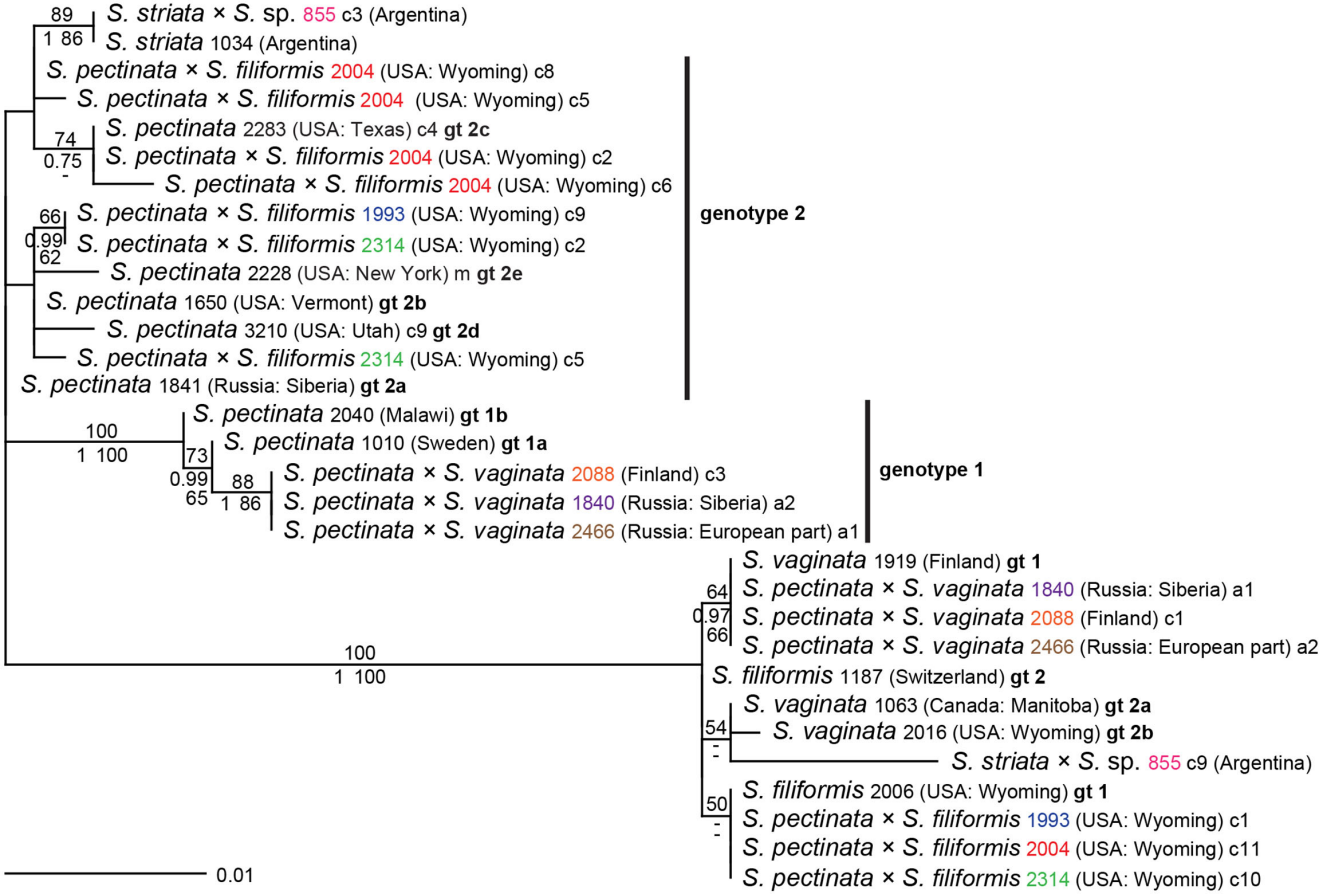
Phylogenetic analyses of *Stuckenia* hybrids with unusual *ITS* genotypes, supplemented with representative genotypes of the parental species. The Maximum Likelihood tree with the highest log likelihood (−1,354.10) is shown with bootstrap values above branches. Posterior probabilities from Bayesian inference and bootstrap support of Maximum Parsimony analyses are below branches. All representative genotypes (gt) of parental species based on **Figure 1** are included for comparison. Several identifiers of hybrids are in color; matching colors represent different alleles (a + number) or clones (c + number) of the same sample. Hybrid sample 2314 contains two novel genotypes of *S. pectinata*, one of which is shared by sample 1993; hybrid sample 2004 contains four clones of *S. pectinata*, one of which matches gt 2c, the others are unique.

Altogether, 57 accessions constituted hybrids in various combinations. While the parental species were often not monophyletic with this marker, the contribution of their particular genotypes to the hybrid samples could be inferred unequivocally in most cases; altogether, five interspecific hybrids with 20 genotype combinations were found (**Supplementary Table 7**). Expectedly, the combination of genotypes usually matched the geographic origins of the samples (**Supplementary Table 1**). For example, hybrids between the mainly Central Asian *S. amblyphylla* and the Eurasian genotype of *S. filiformis* were found in India and Kazakhstan. Eurasian genotypes of *S. pectinata* and *S. vaginata* were found in hybrids from Denmark, Finland, and Russia; American genotypes of these two species occurred in hybrids from Michigan and Maine. Hybrids between Eurasian genotypes of *S. pectinata* and *S. filiformis* occurred in Sweden and Germany; American genotypes of these species were found in Wyoming, like genotype 1 of *S. filiformis*. Hybrids of *S. vaginata* and *S. filiformis* occurred in all combinations of genotypes (**Supplementary Table 7**). Some of these matched the geographic origins of the parental genotypes. For example, the North American genotype of *S. vaginata* was found in hybrids from Wyoming, Michigan, and Canada in combination with the genotype of *S. filiformis* from Wyoming. Likewise, Eurasian genotypes of both species were found in hybrids from European Russia and Siberia. Hybrids occurring in Vermont, Maine, and Michigan combined the North American genotype of *S. vaginata* with the mainly Eurasian genotype of *S. filiformis*, which was, however, also found in one sample from Colorado; this genotype may be more widespread in the entire Northern Hemisphere. In contrast, two hybrid samples of one population from European Russia combined the Eurasian genotype of *S. vaginata* with the *S. filiformis* genotype found so far only in Wyoming. The same genotype of *S. filiformis* also occurred in combination with a so far unsampled genotype of *S. vaginata* in two samples from European Russia; one of them was from the same population as in the above case. Most interestingly, one hybrid from Argentina involving the South American species *S. striata* (855) had a second parent, whose sequence did not correspond to any species in our dataset. In these latter cases, the hybrids revealed unsampled variation in parental species and genotypes. To illustrate this unsampled variation, sequences of the respective accessions were subjected to phylogenetic analyses along with representative genotypes of their parents (**Figure 2**). The hybrid involving *S. striata* showed one sequence that was identical to *S. striata* from the same country. Its second sequence grouped with *S. vaginata* in ML analyses and with *S. filiformis* in BI and MP analyses, always with negligible support. The long branch suggests the contribution of an unknown species or genotype. Three samples of *S. pectinata* × *S. vaginata* (1840, 2088, 2466) from various parts of Eurasia showed the same unique genotype derived from *S. pectinata* genotype 1a. Of three hybrids *S. pectinata* × *S. filiformis* from Wyoming (1993, 2004, 2314), all had *S. filiformis* genotype 1 from that area, but they differed in their genotypes obtained from *S. pectinata*, and two of them showed multiple novel variants of genotype 2 (**Figure 2**).

### Species relationships and intraspecific variation based on *5S-NTS*

This marker was the most variable one that was used in this study. Also, intra-individual variation was higher than in *ITS*; *5S-NTS* sequences contained 3–25 polymorphic sites and up to two indels. Of the major ribotypes used for tree construction (see *Materials and methods*), 37% were without polymorphism, an additional 26% contained only one, and the rest between three and nine polymorphisms (**Supplementary Table 3**). Phylogenetic analysis shows that the distinction into two main clades consisting of *S. pectinata* and *S. striata* and all other species remained the same. However, species relationships were better resolved than with all other markers.

**Figure 3:**
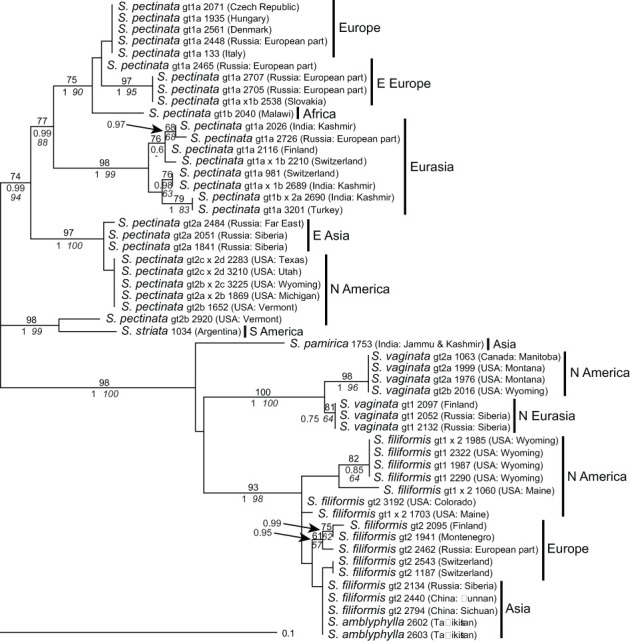
Phylogenetic analyses of *Stuckenia* based on the *5S-NTS*. The Maximum Likelihood tree with the highest log likelihood (−1,182.86) is shown with bootstrap values above branches. Posterior probabilities from Bayesian inference and bootstrap support of Maximum Parsimony analyses are below branches. Genotypes (gt) based on *ITS* sequences are shown for comparison. Geographic origins of the samples are indicated.

***Stuckenia***—For *S. pectinata*, much higher intraspecific variation was found than with *ITS*; several groups of samples showed well-supported monophyletic clades, which also showed a rather detailed geographic structure (**Figure 3**). Interestingly, the South American samples of *S. striata* clustered with one sample of *S. pectinata* from eastern North America; their lineage was most distant from all other samples of *S. pectinata*. Sequences of *S. amblyphylla* from Central Asia were identical to those of *S. filiformis* from Asia whereas *S. filiformis*, *S. vaginata*, and also *S. pamirica* were very well distinguished. Generally, genotypes based on *ITS* sequences matched the genotypes of *5S-NTS*; the latter were split into additional subgroups in *S. filiformis* and *S. pectinata* but not in *S. vaginata*. Some samples that were intraspecific hybrids according to *ITS* did not show character additivity for *5S-NTS* (except for *S. pectinata* 2797, which was therefore excluded from this analysis, see **Supplementary Table 3**).

**Figure 4:**
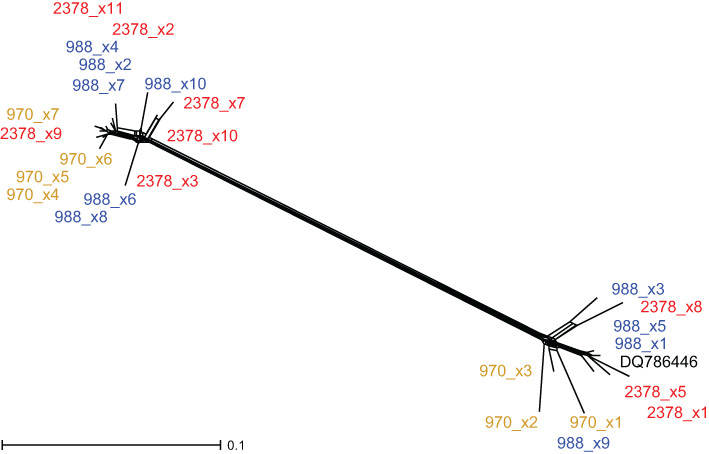
Neighbor Net of *5S-NTS* sequences of *Groenlandia densa*. The positions of cloned sequences (x plus number) of three accessions (970, 988, and 2378) of *G. densa* are illustrated. Matching colors indicate clones of the same sample. A sequence from GenBank (DQ786446) falls into one of the groups.

***Groenlandia***—Sequences of monotypic *G. densa* were not alignable with *Stuckenia* because of their high dissimilarity. Furthermore, each individual contained two main ribotypes (**Figure 4**), which were so divergent from each other that they were even difficult to align with each other. One sample of *G. densa* from GenBank clustered with one of the main ribotypes; otherwise, no similar sequences of other species exist.

### Species relationships based on *rpl*20–5’*rps*12 and comparison with *ITS* genotypes

**Figure 5:**
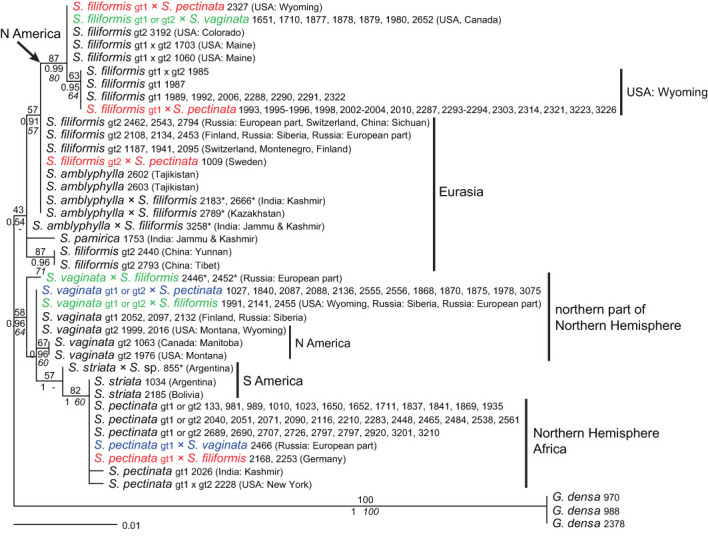
Phylogenetic analyses of *Stuckenia* and maternal origin of hybrids (*rpl*20–5’*rps*12). The Maximum Likelihood tree with the highest log likelihood (−1,404.91) is shown with bootstrap values above branches. Posterior probabilities from Bayesian inference and bootstrap support of Maximum Parsimony analyses are below branches. Asterisks indicate hybrid samples for which the determination of the maternal parent is equivocal. Main genotypes of *S. pectinata*, *S. filiformis*, and *S. vaginata* based on *ITS* sequences are indicated for each species and for the maternal parent of hybrids. Matching colors show the same hybrid with different maternal origins.

Similar to phylogenetic analyses of the *ITS* region, the tree based on the plastid intergenic spacer *rpl*20–5’*rps*12 showed a clade of *Stuckenia pectinata* together with *S. striata* (**Figure 5**). The distinction into divergent genotypes of *S. pectinata* found in *ITS* sequences was not observed; sequences of both species were identical, and only two samples of *S. pectinata* showed slightly derived haplotypes. In contrast to the *ITS* tree, samples of *S. vaginata* were clearly separated from those of *S. filiformis* even though the haplotypes of *S. vaginata* were not monophyletic and the support for the group was only significant in BI (pp 0.96). As with *S. pectinata*, samples of *S. vaginata* showing genotypes 1 or 2 with *ITS* did not differ in their haplotypes, but a slightly derived one occurred in two samples from North America. In *S. filiformis*, however, haplotypes were split into three groups that differed from each other with significant support in all analyses (BS ≥70%, pp ≥0.95). One of these haplotypes was comprised exclusively of North American samples and corresponded to *ITS* genotype 1. A second haplotype occurred in Eurasian samples and was identical to that of *S. amblyphylla*. The third one was found in two out of three samples from China. Samples of the latter two haplotypes had *ITS* genotype 2. *Stuckenia pamirica* nests among haplotypes of *S. filiformis*.

### Identification of the maternal parent of hybrids based on *rpl*20–5’*rps*12

The vast majority of hybrids were unequivocally attributed to one of the parents previously determined by *ITS*. The hybrids *S. pectinata* × *S. filiformis* (*S. ×suecica*), *S. pectinata × S. vaginata* (*S. ×bottnica*), and *S. filiformis × S. vaginata* (*S. ×fennica*) originated independently in different regions, and each of them showed formation by reciprocal crosses (**Figure 5**). Compared to the total number of hybrids, both *S. filiformis* and *S. vaginata* were far more often the maternal parents than *S. pectinata* (only three out of 45 samples). In the case of *S. filiformis × S. vaginata*, both species were approximately equally frequent as maternal parents. A few hybrids’ maternal parents could not be determined with this marker. This concerns hybrids of *S. amblyphylla* and *S. filiformis*; three of these samples showed the main Eurasian haplotype found in both parental species. The haplotype of the fourth hybrid between these species was similar but unique. Another unique haplotype was found in two hybrid samples of *S. filiformis* × *S. vaginata* from European Russia. One of the parents was *S. filiformis* according to *ITS*. The second parent showed a genotype not covered by the sampling (**Supplementary Table 2**). Based on the position of these hybrids’ haplotypes in the *rpl*20–5’*rps*12 tree, the second parent may belong to an unsampled genotype of *S. vaginata*. Also, one hybrid involving *S. striata* and an unknown species or genotype showed a haplotype that did not belong to any of the species sampled; this unknown variant apparently belonged to the maternal parent.

### Species relationships based on *trn*T–*trn*L and comparison with other markers

**Figure 6:**
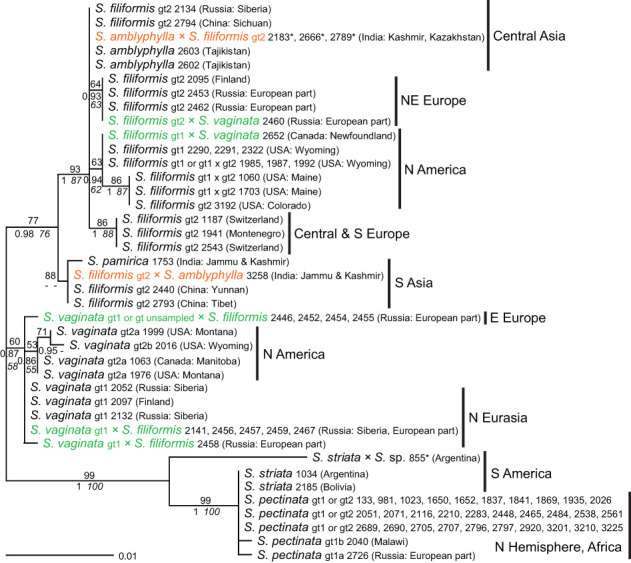
Phylogenetic analyses of *Stuckenia* and maternal origin of hybrids (*trn*T*–trn*L). The Maximum Likelihood tree with the highest log likelihood (−1,543.44) is shown with bootstrap values above branches. Posterior probabilities from Baysesian inference and bootstrap support of Maximum Parsimony analyses are below branches. Asterisks indicate hybrid samples for which the determination of the maternal parent is equivocal. Genotypes of *S. pectinata*, *S. filiformis*, and *S. vaginata* based on *ITS* sequences are indicated for each species and for the maternal parent of hybrids. Matching colors indicate hybrids with the same combination of parents.

The resolution provided by the plastid intergenic spacer *trn*T–*trn*L was higher than with *rpl*20–5’*rps*12. Relationships were basically the same as found previously, but *S. vaginata* and *S. filiformis* were much better distinguished from each other (**Figure 6**). Genotypes of *S. pectinata* determined by *ITS* were not resolved and were also identical to those of *S. striata*. Two samples of *S. pectinata* had haplotypes slightly derived from the main haplotype, but they belonged to different samples than those deviating from the main haplotype in *rpl*20–5’*rps*12. All haplotypes of *S. vaginata* fell into one clade, which was poorly supported but well distinguished from all other species. A slight distinction between *ITS* genotypes 1 and 2 was observed in *trn*T–*trn*L; the haplotypes matched the origins of samples from Northern Eurasia or North America. Like with *ITS*, the haplotype of *S. vaginata* 2016 was derived from other American haplotypes. Haplotypes of *S. filiformis* were even more divergent than with *rpl*20–5’*rps*12 and also showed a more differentiated geographical pattern: they corresponded to samples from Central Asia, northeastern Europe, Central or southern Europe, and South Asia, respectively. American samples comprised *ITS* genotypes 1 and 2, while the four other haplotypes from Eurasia had *ITS* genotype 2. The haplotype of *S. pamirica* was derived from the South Asian haplotype of *S. filiformis*. Sequences of *S. amblyphylla* were identical to those of *S. filiformis* from Central Asia in accordance with the geographical distribution of these species.

### Identification of the maternal parent of hybrids based on *trn*T–*trn*L

The haplotypes of all hybrids showed a clear correspondence with the genotypes of their maternal parents based on *ITS* and also with the geographical regions from which the samples originated (**Figure 6**). Five hybrid sequences of *S. vaginata × S. filiformis* were identical to those of *S. vaginata* from Eurasia, and two unique haplotypes of this hybrid (2458, 2446, and 2452) were derived from it. The previously uncertain maternal parent of hybrid samples 2446 and 2452 from eastern Europe was, due to the better resolution of *trn*T–*trn*L, shown to be derived from northern Eurasian samples of *S. vaginata* with *ITS* genotype 1. Likewise, the maternal parent of one hybrid sample of *S. filiformis* and *S. amblyphylla* (3258) could be unequivocally identified by *trn*T–*trn*L to be *S. filiformis* from southern Asia. However, the direction of the cross of three other hybrids of these species from Central Asia (2183, 2666, and 2789) remained unclear, because both parents from that area shared the same haplotype. Sequences of *trn*T–*trn*L also confirmed reciprocal crosses and independent hybrid origins, at least twice for *S. amblyphylla* × *S. filiformis*, and at least five times for *S. filiformis* × *S. vaginata*. The haplotype of the hybrid between *S. striata* and the unknown species or genotype was more divergent from that of *S. striata* than with *rpl*20–5’*rps*12.

### Species relationships of *Stuckenia* based on the combined dataset

**Figure 7:**
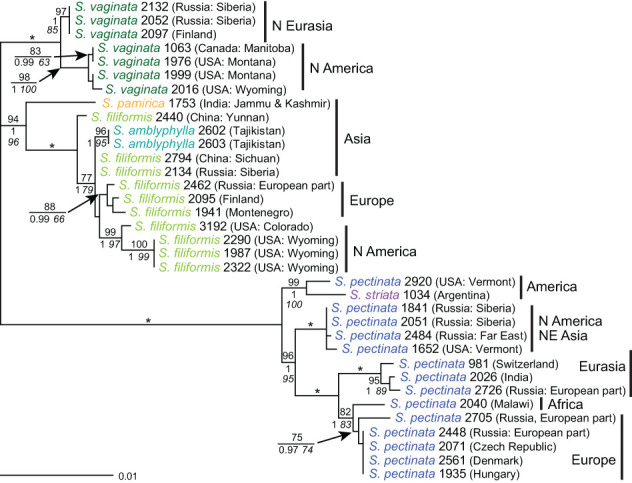
Phylogenetic analyses of *Stuckenia* based on the combined dataset. The Maximum Likelihood tree with the highest log likelihood (−5,275.63) is shown with bootstrap values above branches. Posterior probabilities from Bayesian inference and bootstrap support of Maximum Parsimony analyses are below branches. Asterisks indicate branches with 100% bootstrap support and posterior probability 1. Countries and regions of the samples’ origin are indicated.

Analyses of the dataset of concatenated sequences of all four markers produced a highly resolved tree (**Figure 7**). The majority of the signal was provided by *5S-NTS*, the most variable marker. The distinct position of *S. amblyphylla*, albeit nested within *S. filiformis*, was caused by *ITS*, the only marker showing differences between these species. In contrast, *S. pamirica* differed in all markers from other species but was always nested within *S. filiformis* except for *5S-NTS*. In the tree based on the combined dataset, it was sister to *S. filiformis* with high support. The only non-hybrid sample of *Stuckenia striata* (1034) clustered with one sample of *S. pectinata* from Vermont; another sample of *S. pectinata* from the same state occurred in a branch along with plants from the Russian Far East and Siberia. At the intraspecific level, New World and Old World samples of the widespread species *S. pectinata*, *S. filiformis*, and *S. vaginata* were well distinguished. The only exception was one branch consisting of eastern Asian as well as North American samples of *S. pectinata*. Worth mentioning is also *S. filiformis* 2440 from China. Its basal position was caused by the two plastid markers whereas the nuclear markers did not distinguish it from other Asian material.

### Fertility of the cultivated samples

The capacity of samples to flower and set fruit differed between the taxa. A vast majority of samples of the two most frequent and widespread species, *S. pectinata* and *S. filiformis*, were fertile when grown in standing water. This also holds true for samples originally collected from running and deep waters that remained vegetative in their natural habitats. The exceptions are *S. pectinata* 1023 and 2589, which produced flowers, but no fruit and their entire spikes rotted after flowering. The only available cultivated samples of *S. amblyphylla* (2603), *S. striata* (1034), and another sample morphologically corresponding to *S. striata* (855) that according to molecular markers appeared to be a hybrid with an unknown species or genotype (see above) set normal fruits, and so did one of the samples of *S. vaginata* (2132). The other two cultivated samples of *S. vaginata* either remained vegetative (2052) or flowered, but did not produce fruit in cultivation (1063).

The reproductive behavior of hybrids differed considerably, as not a single interspecific hybrid was fertile. *Stuckenia* ×*suecica* 1009, 2168, and 2252 and *S.* ×*fennica* 1710 flowered, but the flowers were abortive and set no fruit. In contrast *S.* ×*fennica* 1651 and 2141 did not initiate flowering at all in spite of cultivation under suitable conditions for more than 10 years. All samples of *S.* ×*bottnica* (1027, 1839, and 1840) also remained vegetative over long-term cultivation.

None of the hybrid samples collected in the field produced fruit either. Most stands of *S.* ×*suecica* included flowering shoots, both in Europe and the USA. In the case of *S.* ×*fennica*, stands with flowering shoots as well as those containing solely vegetative shoots were recorded in Eurasia and North America. In contrast, most stands of *S.* ×*bottnica* were only vegetative. Flowering shoots were discovered only at the sites of the European samples 2088, 2555, and 2556 and sample 1870 from the USA.

### Comparison of *Stuckenia* sequences with material from other sources

Preliminary alignments with sequences available from GenBank suggest that a considerable number of the samples registered in the database may have been wrongly identified. We, therefore, retrieved all the sequences of the four markers and analyzed them together with the diversity of genotypes present in our samples.

***ITS***—The largest number of additional sequences from GenBank was found for *ITS* (**Supplementary Figure 1**). Most samples corresponded to species from our own collection; sometimes particular genotypes were matched. Three additional taxon names occurred; of these, *S. chakassiensis* is considered a synonym of *S. pectinata*, and *S. subretusa* a synonym of *S. vaginata* (Kaplan, 2008), whereas *S. macrocarpa* falls in the range of genetic variation of *S. pectinata* s. l. (Volkova et al., 2017) and corresponds to our genotype 1a. The origins of the sequences of other authors match the Eurasian *S. pectinata* genotype 1a and also include two samples from Connecticut from an unpublished study on invasive and non-invasive aquatic plants, which suggests they may have been introduced to the New World. Besides our sample from Malawi, genotype 1b of *S. pectinata* occurred in Oman and China. Genotypes 2a–2c of *S. pectinata* were also found in other samples; the North American genotype 2b occurred also in China. Slight variation exceeding that of our genotypes of *S. pectinata* was found, extending their genetic variation and partly also their distribution areas. In some cases, however, the genetic differences of the samples from GenBank may be overestimated due to potential errors in sequence reads (unique single-base indels, especially toward the end of the sequences) and polymerase errors in cloned sequences. Concerning the clade comprising *S. vaginata*, *S. amblyphylla*, *S. filiformis*, and *S. pamirica*, many sequences were identical to genotype 1 of *S. vaginata* or genotype 2 of *S. filiformis*; additional sequences from Canada confirmed a wider distribution of the latter genotype in North America.

Many obvious misidentifications occurred among sequences of *S. pectinata*; they involve samples allegedly belonging to *S. vaginata*, *S. amblyphylla*, *S. filiformis*, and *S. pamirica*, and cloned sequences of hybrids that are attributed to these species. All of these misplaced sequences were from China (**Table 1**). Most surprising was the occurrence of two clones from Wang et al. (2007) named *Potamogeton pusillus*. Two further clones of the same sample fell into the clade comprising *S. vaginata*, *S. amblyphylla*, *S. filiformis*, and *S. pamirica*. Among the latter were also nested single clones from the same study attributed to *Potamogeton obtusifolius* and *P. maackianus* (**Supplementary Figure 1**). As this clade contained many additional genotypes from GenBank and, given that *ITS* did not resolve the relationships of these species, other misidentifications could not be as easily ruled out as for the *S. pectinata* clade. One exception was a sample from Canada attributed to *S. pectinata* (Kuzmina et al., 2017), which may belong to a misidentified herbarium specimen in this extensive study or may represent PCR contamination. Its sequence was identical to a genotype of *S. vaginata* from the same country published later by the same group (Kuzmina et al., 2018).

**Table 1:** Misidentified samples of *Stuckenia* in GenBank.

Marker	Accession Numbers	Erroneous Identification	Source	Remarks
*ITS*	DQ840297–DQ840300	*Potamogeton pusillus*	Wang et al. (2007)	four clones of the same sample in different clades, wrong genus
	DQ840271	*Potamogeton maackianus*	Wang et al. (2007)	one clone of this sample, wrong genus
	DQ840315	*Potamogeton obtusifolius*	Wang et al. (2007)	one clone of this sample, wrong genus
	DQ840335–DQ840337	*Stuckenia vaginata*	Wang et al. (2007)	three clones of the same sample, all wrong clade, one or two of them probably recombinant
	FJ956806–FJ956807	*Stuckenia pamirica*	Du et al. (unpubl.)	two samples, wrong clade
	FJ956921–FJ956922	*Stuckenia pamirica × S. filiformis*	Du et al. (unpubl.)	two clones of the same sample, both wrong clade
	FJ956920	*Stuckenia amblyphylla × S. pamirica*	Du et al. (unpubl.)	one clone of this sample in the wrong clade
	FJ956808–FJ956810	*Stuckenia filiformis*	Du et al. (unpubl.)	three samples, wrong clade
	FJ956923–FJ956926	*Stuckenia pectinata × S. filiformis*	Du et al. (unpubl.)	two clones of two samples, but no *S. filiformis*
	MG216537	*Stuckenia pectinata*	Kuzmina et al. (2017)	wrong clade
	KY407966	*Stuckenia filiformis*	Volkova et al. (2017)	potentially wrong, very similar to *S. vaginata* KY407968 and KY407969
*5S-NTS*	FJ495502	*Potamogeton pusillus*	Zhang and Wang (unpubl.)	wrong genus
	FJ495517	*Stuckenia vaginata*	Zhang and Wang (unpubl.)	wrong clade
	FJ495521–FJ495522	*Stuckenia pamirica*	Zhang and Wang (unpubl.)	groups with *S. filiformis*, but *S. pamirica* from India is well distinguished with this marker
*trn*T–*trn*L	EF471051	*Stuckenia filiformis*	Zhang et al. (2008)	similar to *Potamogeton gramineus*
	EF471064	*Stuckenia vaginata*	Zhang et al. (2008)	wrong clade

***5S-NTS***—Several samples from other sources clustered with conspecifics of our material and also matched the geographical origins of the samples at the intraspecific level (**Supplementary Figure 2**). One sample of *S. macrocarpa* was most similar to *S. pectinata* from Russia and other regions. One sample of *S. striata* from North America fell into a different clade of *S. pectinata* than our sample of *S. striata* from South America. One sequence attributed to *S. vaginata* occurred among samples of *S. pectinata*; this sample was apparently misidentified (**Table 1**). A further mistake was a sample named *Potamogeton pusillus* (not the same sample as with *ITS*) from an unpublished study, which occurred among sequences of *S. filiformis*. Two samples of *S. pamirica*, most probably from China, also fell into the clade comprising *S. filiformis* and *S. amblyphylla* sequences. Our sample from India was well distinguished with this marker, so the Chinese samples might be misidentified. However, we did not have access to further samples of the rare *S. pamirica* from other regions and therefore could not access its intraspecific variation. Several unique mutations or single base indels occurred in the Chinese sequences of *S. pamirica* and also in two samples of *S. filiformis*. These mutations might represent either reading errors or further genotypes within the Eurasian clade of *S. filiformis*.

***Rpl*20–5’*rps12***—For this marker, only four sequences of *S. filiformis* from Du and Wang (2016) were present in GenBank; all samples originated from the Qinghai–Tibetan Plateau. Their haplotype 1 was identical to our samples of that species from the Chinese provinces of Tibet and Yunnan; their haplotype 2 was identical to our most widespread haplotype found in samples of *S. filiformis* from all over Eurasia (and in *S. amblyphylla*). Their haplotypes 3 and 4 differed slightly from our material (**Supplementary Figure 3**), but the identification of these samples as *S. filiformis* is most likely correct.

***TrnT-trnL***—Analysis of the *trnT-trnL* region revealed new haplotypes of *S. pectinata* from China and Japan (**Supplementary Figure 4A**). While the Chinese haplotype showed unique mutations compared to the most abundant haplotype, the Japanese haplotype showed exclusively character states that alternated between those of *S. pectinata*/*S. striata* and those of the clade comprising the rest of the species, which explains its somewhat intermediate position in the tree. Another material from Japan is not available, so it remains unclear whether this sequence pattern is typical for that area or whether it represents some kind of artifact. One clearly misidentified sample of *S. vaginata* occurred among sequences of *S. pectinata* and *S. striata* (**Table 1**). Two samples identified as *S. pamirica* from China had identical haplotypes to samples of *S. filiformis* and *S. amblyphylla* (**Supplementary Figure 4A**), which stems from the misapplication of this name to robust forms of *S. filiformis* in the Chinese literature (see *Discussion*). One Chinese sample of *S. filiformis* from GenBank showed a haplotype identical to one Chinese sample from our collection; another haplotype of *S. filiformis* was unique but derived from the former. In contrast, a further sample from China determined as *S. filiformis* had a sequence that was most similar to *P. gramineus* and related taxa (**Supplementary Figure 4B**). Confusion about these two species is hardly possible, therefore, PCR contamination with samples of *P. gramineus* from the same study (Zhang et al., 2008) is likely.

## Discussion

We present the most comprehensive molecular study of the aquatic plant genus *Stuckenia* based on worldwide sampling and four DNA markers. Species relationships, intraspecific variation, and hybridization were analyzed in detail and related to the geographical origins of samples, their morphology, and the fertility of the plants.

### Species relationships

All markers show a distinction into two main clades, one comprising worldwide distributed *S. pectinata* and the American species *S. striata*; the other is formed by *S. filiformis*, which is widespread in the Northern Hemisphere, *S. amblyphylla*, distributed in western and Central Asia, *S. pamirica*, confined to Central Asia, and *S. vaginata*, which occurs in the Northern Hemisphere. Three core groups, represented by three widespread, traditionally recognized species, *S. pectinata*, *S. filiformis*, and *S. vaginata*, are particularly obvious. The positions of the remaining species are discussed in the following paragraphs.

*S. striata* is not well distinguished from *S. pectinata* (genotype 2) at the DNA level, which is consistent with the mainly quantitative morphological differentiation of these species (Haynes and Hellquist, 2000). The fact that *ITS* sequences of one of the main genotypes of *S. pectinata* are more similar to those of *S. striata* than to the other main groups of *S. pectinata* (**Figure 1**) indicates that *S. striata* evolved from that variable species. In particular, the position of one sample of *S. striata* in the *5S-NTS* tree (**Figure 3**) suggests it is derived from a particular North American lineage of *S. pectinata* and may have spread throughout South America. One North American accession of *S. striata* from GenBank (**Supplementary Figure 2**) corresponds to a different genotype of *S. pectinata*. However, this may have been caused by unrecognized hybridization in that GenBank sample. In any case, intermediate forms that are difficult to assign to one of these two species are occasionally found. The exact delimitation between *S. pectinata* and *S. striata* requires further study based on more representative material from both North and South America.

*S. amblyphylla* from Central Asia nests within *S. filiformis* except with *ITS*, the only marker that is able to distinguish this species at the molecular level. Again, this pattern is consistent with the morphological similarity of these two species (Kaplan, 2008). *S. amblyphylla* appears to be a specialized lineage that has evolved in the mountains of Central Asia from the variable and widespread *S. filiformis*.

*S. pamirica*, a species confined to high elevations in the mountains of Central Asia, is resolved as a sister species to *S. filiformis*/*S. amblyphylla* (**Figure 7**). A comparison of sequences retrieved from GenBank with our sequences indicates that *S. pamirica* continues to be misunderstood as this name is occasionally assigned to plants of *S. filiformis* or *S. pectinata* (**Supplementary Figure 1**). Although the name *S. pamirica* (as *P. pamiricus*) was widely misapplied to broad-leaved forms of *S. filiformis* (the confusion dates back to Hagström, 1916; see the review in Kaplan, 2008), the proper species is actually more similar to *S. pectinata* morphologically, with which it also shares open leaf sheaths, whereas *S. filiformis* has leaf sheaths that are closed and tubular at the base. However, genetically, *S. pamirica* is more closely related to *S. filiformis*, *S. amblyphylla*, and *S. vaginata*. Still, its ribotypes and haplotypes are unique, represented by long branches in phylogenetic trees, which all support the refined concept proposed by Kaplan (2008) and the taxonomic treatment of *S. pamirica* as a distinct species.

### Intraspecific diversity

At the intraspecific level, all markers show a distinction of several genotypes within the three widespread species. Nuclear markers show high intraspecific variation in *S. pectinata*, similar to or even exceeding the interspecific variation of other species. In contrast, plastid markers are uniform in *S. pectinata*. The opposite pattern is found in *S. filiformis* and *S. vaginata*, where plastid markers show higher intraspecific haplotype diversity than genotype diversity in the *ITS* region and similar diversity to *5S-NTS*.

Effective population size is smaller and the time to fixation of ptDNA haplotypes within a population is shorter than that of nuclear DNA, which results in the faster coalescence of plastid markers (Small et al., 2004). Therefore, the resolution of ptDNA for geographic patterns is often better than that of nuclear markers (Whittemore and Schaal, 1991; Acosta and Premoli, 2010; Zhao et al., 2020). On the other hand, while nuclear markers often show more diversity due to their higher genetic variation compared to ptDNA, one has to take into account the unpredictable behavior of multicopy nuclear markers that are prone to concerted evolution (Álvarez and Wendel, 2003). *ITS* sequences in *Stuckenia* are surprisingly well homogenized with hardly a hint of intra-individual polymorphism given that the entire genus is hexaploid. This is an indication that the genomes have been strongly diploidized for a long time. This is also evident for *5S-NTS* sequences. Even though they are much more polymorphic and less well homogenized than *ITS* sequences in *Stuckenia* (but less than in the sister genus *Potamogeton*, Kaplan et al., 2018; Fehrer et al., 2022), the dominant sequence types clearly reveal many distinct genotypes within each of the three widespread species that fit well with the geographical origins of the samples. *ITS* sequences also reveal geographical structure at the intraspecific level but it is less prominent due to the low variation of the marker.

Additional sequences from GenBank revealed further genotypes of the same species and, in some cases, larger geographical distributions of the genotypes contained in our study. Part of this additional variation may reflect errors in sequence reads or polymerase errors of cloned sequences. However, without access to the original sequence data, it is not possible to assess the degree of true genotypic variation. On the other hand, many obviously erroneous identifications were revealed in other studies (summarized in **Table 1**) that were not caused by alternative taxonomic treatments (see below); some of these samples are even assigned to several species of the genus *Potamogeton*. While *Stuckenia* and linear-leaved *Potamogeton* species (e.g., *P. pusillus*, *P. obtusifolius*) are superficially similar and may be confused by inexperienced botanists, identifications of *Stuckenia* as broad-leaved *Potamogeton* species (*P. maackianus* —Wang et al. (2007), **Supplementary Figure 1**; *P. gramineus*—Zhang et al. (2008), **Supplementary Figure 4B**) are likely caused by contamination or mixed up samples.

### Taxonomic interpretations and conclusions

Because of the extensive phenotypic plasticity and occurrence of many local or regional forms, numerous infraspecific taxa and even separate species have been described (see reviews, e.g., in Hagström, 1916; Wiegleb and Kaplan, 1998; Kaplan, 2008; Kaplan, 2010a). Only selected conspicuous phenotypes and taxa most frequently distinguished in the recent literature are discussed here.

*Stuckenia pectinata—S. pectinata* is the most widespread and variable species in the genus. It includes numerous phenotypes as well as local forms. However, much of the observed variation is due to phenotypic plasticity and ontogenetic variation (Kaplan, 2002, and the review therein). Two major genotypes with several largely geographically correlated variants are identified in this study, but no consistent morphological differences between them are found. A similar genetic pattern and a lack of morphological differentiation were previously observed in plants from southern Siberia (Volkova et al., 2017). The origin of this morphological variation was insufficiently understood in the past. Consequently, far too many taxa have been proposed. Minor variants were mostly described as varieties, while more distinctive phenotypes were treated as different species. Some of these are discussed in the following paragraphs.

*S. helvetica* (G. Fisch.) Holub was described (as *Potamogeton vaginatus* var. *helveticus*) from deep water in the perialpine lake of Untersee (Lower Lake Constance) in northern Switzerland. It is a robust form with wintergreen stems and large inflated sheaths on the basal parts of the stems. In particular, the overwintering young pioneer shoots sprouting at the beginning of spring are the most divergent feature. Morphologically corresponding plants were recollected from the type population (samples 2210–2213). In cultivation, they produced phenotypes corresponding to those that *S. pectinata* usually produces later in the season. *S. helvetica* has proven to be sterile as it sets no fruit in spite of abundant flowering for several seasons. *ITS* sequencing revealed that it is an intraspecific hybrid between *S. pectinata* genotypes 1a and 1b. Very similar plants were detected in the Gudenå River in Denmark (samples 1023, 2587, and 2589), which were also sterile and of the same genotype combination. Herbarium studies revealed the occurrence of similar forms also elsewhere in the Eurasian part of this species’ range (e.g., in the southern part of European Russia, Kazakhstan, and Turkey). They seem to be local ecotypic adaptations to deep water that have evolved under suitable conditions recurrently and independently of each other. Although these are sterile, their colonies are maintained due to vegetative persistence. No taxonomic status is assigned here to these robust forms.

Another distinct form characterized by broad and obtuse to rounded leaves that occurs mainly along the coasts of the Gulf of Bothnia and the Gulf of Finland in northern Europe was described as *P. zosteraceus* Fr. and more recently transferred to *Stuckenia* (Tzvelev, 1999). We managed to find and investigate only one sample corresponding to this form (2090). *ITS* sequencing showed that it is an intraspecific hybrid between *S. pectinata* genotypes 1a and 2a. The population included flowering and fruiting plants, and the morphology of the plant remained stable in cultivation. Herbarium studies as well as the literature (e.g., Hagström, 1916) indicate that this form is geographically confined to a relatively small geographical area, which suggests that it is a specific genotype and not a recurrently produced form. However, although these forms are morphologically markedly different from the most common phenotypes of *S. pectinata*, they are connected by all sorts of intermediates so that no clear-cut demarcation between them can be drawn (Kaplan, 2008). In addition, some of these phenotypes seem to be under environmental control and others may be associated with ontogenetic variation (Van Wijk, 1988; Kaplan, 2002; Kaplan, 2008). For all these reasons, *S. zosteracea* cannot be consistently distinguished from ordinary *S. pectinata* and is therefore not recognized here.

Local broad-leaved forms are also found in other parts of the range of *S. pectinata*. Those from Lake Malawi in southeastern Africa were described as *P. livingstonei* A. Benn. Our sample (2040) from the same lake has *ITS* genotype 1b, which differs by a single substitution from Eurasian samples of genotype 1a, and as it is identical to the major sequence of sample 2228 from the USA, we do not consider this minor variation sufficient to recognize *P. livingstonei* as a distinct species.

Additional species have been described from Siberia. Sequences of *S. chakassiensis* (Kaschina) Klinkova are identical to one of the variants of *S. pectinata* genotype 1 (**Supplementary Figure 1**). The morphological differentiation between them is also weak, which justifies the inclusion of the former into the latter (Kaplan, 2008; Volkova et al., 2017). Although *S. macrocarpa* is also placed among samples of the variable *S. pectinata* in the *ITS* tree (**Supplementary Figure 1**), it represents a unique genotype that is associated with specific morphology and a restricted geographic range (Kaplan, 2008), which led to its previous recognition as a separate species. However, in the areas of co-occurrence with *S. pectinata*, morphological intermediates between these forms have recently been observed (A. Bobrov, unpublished data), which does not allow reliable morphological separation of *S. macrocarpa*. *S. macrocarpa* may represent a lineage of the variable *S. pectinata* s. l. in an early stage of speciation, which has originated in the lowlands of west-central and northern Kazakhstan and adjacent parts of western Siberia and has so far accumulated only a very few genetic differences. Because of the weak genetic differentiation, *S. macrocarpa* was lumped into the broadly delimited *S. pectinata* by Volkova et al. (2017).

Phenotypes from running water have particularly confused researchers. They usually tend to have wider and more elongated leaves and flower whorls in a spike that is more remote compared to the same genotype growing in standing water. These forms have been repeatedly described, and the earliest name for these forms is *P. interruptus* Kit., published in Hungary (Schultes, 1814). A lectotype is a form of *S. pectinata* (Kaplan, 2008). However, in the past decades, this name has been applied to plants from various parts of Russia (Busik, 1979; Tzvelev, 1984; Tzvelev, 1996). We investigated Siberian plants identified in accordance with this concept (samples 1834, 1840) and these proved to be *S. pectinata × S. vaginata* (*S. ×bottnica*). Bobrov and Chemeris (2006) studied a river population in European Russia and proved it to be *S*. *pectinata*.

*Stuckenia filiformis—*The second most widespread species is *S. filiformis*. It also produces numerous phenotypes. The robust forms from running waters are recorded from almost the entire range of this species and have been repeatedly described as *P. juncifolius* C. Fritsch from Europe, *P. austrosibiricus* Kaschina from Siberian rivers, and *P. filiformis* var. *occidentalis* (J. W. Robbins) Morong from North America. Our samples corresponding to these phenotypes are genetically either identical to local ordinary genotypes of *S. filiformis* (2543, Europe, “*juncifolius*”; 2134, Siberia, “*austrosibiricus*”), or were resolved as interspecific hybrids. Sample 2141 (Siberia), first identified as *P. austrosibiricus*, was resolved as *S. filiformis × S. vaginata* (*S. ×fennica*), whereas the North American samples corresponding to the current usage of *P. filiformis* var. *occidentalis* were resolved partly (samples 1651, 1710, 1877–1879) as *S. filiformis × S. vaginata* (*S. ×fennica*) and partly (samples 1868, 1870, and 1875) as *S. pectinata × S. vaginata* (*S. ×bottnica*). This indicates that most, if not all, the names applied to describe the claimed taxonomic variation in *S. filiformis* refer to mere phenotypes responding to specific environments and to previously unrecognized hybridization.

*S. amblyphylla* is similar as well as closely related to *S. filiformis*, with which it shares a unique character—leaf sheaths that are closed and tubular at the base (connate)—that separates these two from all other *Stuckenia* species. In contrast to many forms of *S. filiformis*, *S. amblyphylla* is primarily distinguished by the size of its fruits and the shape of its spikes. Unfortunately, most specimens lack mature fruits and vegetative characters are less reliable (Kaplan, 2008). Consequently, the exact extent of morphological and genetic variation and geographical distribution is insufficiently known (Kaplan et al., 2013; Aykurt et al., 2020).

*Stuckenia filiformis—*The third widespread species, *S. vaginata*, is usually easily distinguishable. In addition, an apparently similar taxon, *S. subretusa* from the Arctic, is distinguished in Russian literature (e.g., Tzvelev, 1987; Kashina, 1988; Tolmachev, 1995). The key morphological character claimed for this taxon, the (sub)retuse apex, was found sometimes to occur in the ordinary specimens of *S. vaginata* and is unreliable for identifying this taxon. The same is true for some other characters used to distinguish *S. vaginata* and *S. subretusa*, such as the size of leaf laminas and sheaths and the number of branches growing from a node, because they show large overlaps (Kaplan, 2008). However, both forms differ in the branching pattern and the length of ligula, partly also in fruit size, and also in their Asian distribution ranges: *S. vaginata* occurs mainly in southern Siberia, whereas *S. subretusa* is found in the Arctic (Volkova et al., 2017; Bobrov, 2020). The only sequence of *S. subretusa* available in GenBank is identical to one of the variants of *S. vaginata* from Canada (**Supplementary Figure 1**) but differs from Eurasian samples of this species. Our specimens from the Russian Arctic with some leaves with a retuse apex were resolved either as *S. filiformis* (sample 2453) or as *S. filiformis × S. vaginata* s. l. (samples 2452, 2454, and 2455). Leaving misidentifications aside, *S. subretusa* appears to be an Arctic lineage derived from *S. vaginata*. The entire group deserves further study based on more representative material from both the Eurasian and North American parts of its range.

### Interspecific hybridization

Numerous interspecific hybrids are determined by analyses of the *ITS* region, and their maternal parents can, in most cases, be identified with plastid markers. Usually, particular combinations of genotypes of the respective species can be revealed. All hybrids originated multiple times, and both directions of the cross are realized.

Three interspecific *Stuckenia* hybrids were known prior to this study. All were discovered and described in northern Europe (Hagström, 1916), and most of the known occurrences are in the British Isles, Scandinavia, and northeastern Europe (Preston, 1995; Bobrov, 2007). Although many DNA-based studies are available for the closely related genus *Potamogeton* (e.g., Kaplan and Fehrer, 2007; Kaplan and Fehrer, 2009; Zalewska-Gałosz et al., 2009; Zalewska-Gałosz et al., 2010; Kaplan et al., 2011; Kaplan and Fehrer, 2013; Aykurt et al., 2017; Zalewska-Gałosz et al., 2018; Iida et al., 2018; Kaplan et al., 2019), the molecular evidence for hybridization in *Stuckenia* is rare and available for only a few countries: *S. filiformis × S. pectinata* was documented from Norway and the United Kingdom (McMullan et al., 2011), *S. filiformis × S. vaginata* from Denmark and Russia (McMullan et al., 2011), and *S. pectinata × S. vaginata* from the United Kingdom (King et al., 2001; McMullan et al., 2011), Denmark, and Ireland (McMullan et al., 2011). This study provides the first DNA proofs for the hybrid origins and the parentages of *S. filiformis × S. pectinata* from Sweden, Germany, and the USA, that of *S. filiformis × S. vaginata* from the Siberian part of Russia, Canada, and the USA, and that of *S. pectinata × S. vaginata* from Finland, Russia, and the USA. In addition, for the first time, we indicate probable hybridization between *S. filiformis* and *S. amblyphylla*. However, the delimitation between these two species is insufficiently understood, and their genetic divergence and gene flow require further study.

Our fieldwork and herbarium studies as well as the cultivation experiments confirm the previous observations (e.g., Hagström, 1916; Preston, 1995; Preston et al., 1998; Preston et al., 1999; Bobrov, 2007; Kaplan, 2008) that interspecific hybrids in *Stuckenia* are consistently sterile. They either produce flowers (usually only some ramets in a clone), which are, however, abortive, or they remain in a vegetative state. However, in contrast to many plant genera whose hybrids are sterile, short-lived F1 plants, a sterile hybrid in *Stuckenia* can persist vegetatively for a long time and even spread as stem fragments. Previous studies have demonstrated that hybrid pondweeds can persist in a locality for a considerable period, even hundreds or thousands of years, and sometimes even after one or both parents have disappeared (e.g., Kaplan and Wolff, 2004; Zalewska-Gałosz, 2010; Kaplan and Fehrer, 2011; Kaplan and Uotila, 2011; Kaplan and Fehrer, 2013; Zalewska-Gałosz et al., 2018; Kaplan et al., 2018), provided that the ecological conditions remain suitable. For example, the distribution of *S.* ×*suecica* in England, south of the present limit of distribution of *S. filiformis*, suggests that these clones are relicts from glacial periods (Hollingsworth et al., 1996). The occurrence of *S.* ×*bottnica* in Britain further indicates that this hybrid may have persisted in the islands for thousands of years, while one of its parents, *S. vaginata*, is now restricted in Europe to Scandinavia, and in Britain, it is documented only from deposits which date back to the first British glacial period (Preston et al., 1998; King et al., 2001). Our sampling and molecular analyses revealed the occurrence of relict hybrids outside the distribution of one of their parental species in several countries and regions. For example, stands of *S.* ×*fennica* in the northeastern USA and eastern Canada, and those of *S.* ×*bottnica* in Denmark and the northeastern USA, are found substantially outside the current range of *S. vaginata* (see Hultén and Fries, 1986; Haynes and Hellquist, 2000; Kaplan, 2008). This indicates that this boreal species used to be more widespread and had a more a southerly distribution in these areas during the last glacial period than it does today.

It should be pointed out that the absence of flowers and fruits in a population alone does not prove the true genetic sterility of these plants and, consequently, their hybrid origin. In particular, *S. pectinata*, but often also *S. filiformis* fails to fruit when growing in rivers with a fast current (Van Wijk, 1988; Preston, 1995; Kaplan, 2008). However, these vegetative clones growing in running water also produce normal, well-formed fruits in hot summers after the water level and flow are reduced or when experimentally transplanted to standing water (Kaplan, 2008).

Although we recorded many hybrid populations during our fieldwork and included more than five dozen of them in this study, the number of hybrids in our sampling is certainly overrepresented compared to the actual frequency of *Stuckenia* hybrids in the field. Firstly, it should be emphasized that our sampling was aimed to cover the existing diversity as far as possible, and we intentionally collected all samples that morphologically appeared to be something else than ordinary species. Second, hybrids are likely to occur only in areas where two or more species co-occur at present, or where they co-occurred in the past and where rivers and lakes that were more or less stable for hundreds or thousands of years are present, allowing long-term persistence of the sterile hybrid clones. On the other hand, in such areas and in these particular habitats, *Stuckenia* hybrids may be locally frequent and occasionally even dominant (Hagström, 1916; Preston, 1995; Bobrov, 2007). The correct morphological identification of *Stuckenia* hybrids is, however, extremely difficult (Preston, 1995; Preston et al., 1998; Preston et al., 1999), requires specific long-term experience, and therefore, molecular analysis of questionable plants is often necessary. Besides, some genotypic variation contained in hybrids exceeded that of their parents, indicating some unsampled genetic variation.

The origin of the hexaploid genus *Stuckenia* (*2n* = 6x = 78) is unknown; hybrids with other genera have never been found (Kaplan et al., 2013). However, its sister genus *Potamogeton* comprises many species with *2n* = 2x = 26, which is indicative of a common ancestor with this chromosome number (Kaplan et al., 2013).

### Intraspecific hybridization

Besides hybrids between recognized species, hybridization was also revealed between conspicuous genetic variants detected within the widespread variable species *S. pectinata* and *S. filiformis*. Within *S. filiformis*, three samples, all from the USA, are resolved as intraspecific hybrids between *ITS* genotypes 1 and 2 (**Supplementary Table 7**). Of these, two (1060 and 1703) are fertile; the third one has not been tested for fertility. The situation is more complex in *S. pectinata*. This species consists of two major *ITS* genotypes, each of which includes additional minor variants. Several intraspecific hybrid combinations are detected in our sampling. Most of these plants were not cultivated and thus were not available for fertility tests. Of the samples we were able to test, 1023 and 2589, which are intraspecific hybrids between the *ITS* genotypes 1a and 1b, are sterile, with abortive flowers. In contrast, sample 2538 of the same genotype combination, sample 2090, which is a hybrid between genotypes 1a and 2a, and 3210, a hybrid between genotypes 2c and 2d, are all fertile, flowering, and fruiting. Several other samples identified as intraspecific hybrids within *S. pectinata* (1711, 1869, 2689, 2690, and 3225) were flowering in the field, but the further development of these flowers is unknown. Well-developed fruits and well-formed pollen were observed in intraspecific hybrids between all four lineages of *S. pectinata* s. l. in southern Siberia (Volkova et al., 2017). It is clear, however, that the genetic divergence of *ITS* sequences contained within a sample is not indicative of their status as inter- or intraspecific hybrids. For example, *S. pectinata* plants combining the much more divergent genotypes 1a and 2a can obviously be fertile. However, those combining the much more similar genotypes 1a and 1b are sterile. The same is true for interspecific hybrids; for example, all hybrids between *S. filiformis* and *S. vaginata* are sterile despite the very low sequence divergence of *ITS* sequences, whereas intraspecific hybrids of *S. pectinata* are usually fertile even though their genotypic variation exceeds that of *S. filiformis* and *S. vaginata* combined.

For these reasons, our knowledge concerning the reproductive isolation of intraspecific hybrids is still insufficient. The present data suggest the interbreeding of different genotypes in the case of *S. filiformis*, which is in keeping with the biological species concept. In the case of *S. pectinata*, there are obvious differences in the fertility of particular genotypes, and some of them correspond to particular morphological forms and/or geographic areas. We assume that we are observing here some incipient speciation and that some genotypes may already represent cryptic species, as it was also found in the sister genus *Potamogeton* (Aykurt et al., 2020; Fehrer et al., 2022). However, without a deeper understanding of intraspecific processes—and given that all genotypes of *S. pectinata* together form monophyletic clades with all markers, which are genetically widely divergent from other species (except *S. striata*, with which it is paraphyletic)—it would be unwise to propose taxonomic consequences at the intraspecific level.

The most puzzling results from molecular analyses concern an apparent hybrid between *S. striata* and an unknown species or genotype (sample 855) and the monotypic outgroup *G. densa*. The particular sample of *S. striata* is fertile, which suggests that it is not a hybrid. This is despite the large genetic divergence of the second ‘parent’ from other genotypes of *S. striata*. Besides, no other species of *Stuckenia* in South America are known with which it could have hybridized. We, therefore, assume that this species may contain a large variety of genotypes, similar to the closely related *S. pectinata*, and that the aberrant lineage reflects unsampled variation. A broader sampling of *S. striata* in South America and detailed molecular analyses will be necessary for clarification. Concerning *G. densa*, there are no closely related species, and with all molecular markers, it is widely divergent from other Potamogetonaceae. However, its *5S-NTS* sequences show the presence of two highly divergent paralogs in three samples from Austria, Switzerland, and Slovakia, respectively (**Figure 4**). One sample from GenBank (DQ786446) falls into one of these groups. The sample is from a study by Lindqvist et al. (2006) and originates either from Spain (as indicated in Genbank) or from Denmark (as indicated in the paper). Polymorphisms in *5S-NTS* were allegedly negligible in this sample. We assume that either the second paralog is absent in that sample or that it was ignored like in several samples of *Potamogeton* (see Discussion in Kaplan et al., 2018). Our samples of *Groenlandia* are fertile. In its sister genera, *Potamogeton* and *Stuckenia*, *5S-NTS* maintain hybrid signatures longer than *ITS* as the latter homogenizes faster (Kaplan et al., 2018; this study). However, the divergence of the *5S-NTS* sequences of *Groenlandia* by far exceeds the variation found between the most divergent genotypes within any sample of the sister genera, even including hybrids between distantly related species. We, therefore, assume that *Groenlandia 5S-NTS* sequences consist of two paralogs that evolved independently for a long time. This is in keeping with a single origin of *Groenlandia*, suggested also by all other molecular markers and its unique morphology and chromosome number.

## Conclusion

We present a detailed study of the aquatic plant genus *Stuckenia* based on worldwide sampling. By applying various molecular markers, we were able to clarify taxonomic species delimitations, describe intraspecific variation and its geographic structuring, and identify a large amount of intra- and interspecific hybrids. We showed that genetic distance is not always correlated with the biological differentiation of the species. Phenotypic variation supported by cultivation experiments as well as assessments of fertility were integrated into an overall interpretation of the speciation patterns. This resulted in much more refined taxonomic circumscriptions. Due to the difficulties of morphological species determination in this group, many herbaria as well as public sequence databases contain a large number of misidentified samples. Our study emphasizes that reliable taxonomic conclusions should take a complementary approach that ideally combines data from morphology, distribution, cytology, reproductive biology, and genetics.

## Data availability statement

The datasets presented in this study can be found in online repositories. The names of the repository/repositories and accession number(s) can be found below: https://www.ncbi.nlm.nih.gov/genbank/, OP101176–OP101375, OP136177–OP136533.

## Author contributions

JF conceptualized the study. ZK, CBH, and AB collected the materials. ZK determined the plants and tested fertility. MN did labwork. JF and MN analyzed the data. JF and ZK wrote the manuscript. All authors contributed to the article and approved the submitted version.

## Funding

The Czech Science Foundation (grant number 17-06825S), the Czech Academy of Sciences (long-term research development project, grant number RVO 67985939), the Papanin Institute for Biology of Inland Waters RAS state assignment (theme 121051100099-5), and the Tyumen Oblast Government, as part of the West-Siberian Interregional Science and Education Center’s project No. 89-DON (2) are acknowledged for financial support.

## Acknowledgments

The authors thank V. Bambasová and A. Yadollahi for parts of the labwork, V. Chepinoga, O. Mochalova and G. Klinkova for collecting some Russian material, and the curators of the visited herbaria, who allowed us to study their collections.

## Conflict of interest

The authors declare that the research was conducted in the absence of any commercial or financial relationships that could be construed as a potential conflict of interest.

## Publisher’s note

All claims expressed in this article are solely those of the authors and do not necessarily represent those of their affiliated organizations, or those of the publisher, the editors and the reviewers. Any product that may be evaluated in this article, or claim that may be made by its manufacturer, is not guaranteed or endorsed by the publisher.
