# Supplementary material for: Evolutionary history and patterns of geographical variation, fertility, and hybridization in Stuckenia (Potamogetonaceae)
Source: Front Plant Sci. 2022 Nov 3;13:1042517. doi: 10.3389/fpls.2022.1042517 (PMC9670304; doi:10.3389/fpls.2022.1042517)
Supplement: Supplementary file 1 [file Image_1.pdf]

Supplementary Figure 1 |
Placement of *ITS* sequences from GenBank among species and genotypes of this study

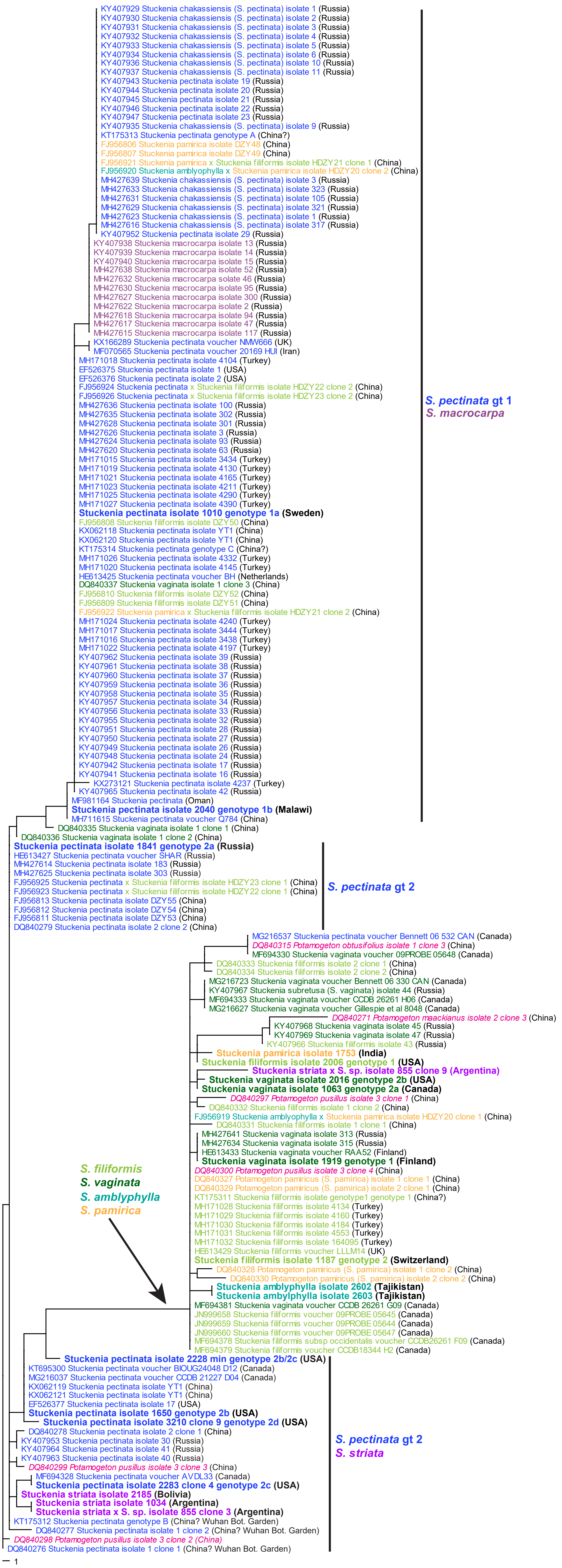

A Neighbor Joining tree shows 19 representative sequences of our collection (in bold) and 153 sequences from other sources; countries of origin are given for each sequence. For better overview, sequences of the same nominal species are shown in matching colors. *Stuckenia chakassiensis* is a synonym of *S. pectinata*, and *S. subretusa* is a synonym of *S. vaginata* (Kaplan 2008); the names are maintained as they occur in GenBank, but the synonyms are given in brackets, and the color matches the correctly named species. Samples erroneously attributed to genus *Potamogeton* are in pink and italics. Obviously misidentified samples are summarized in Table 1.
