# Supplementary material for: Evolutionary history and patterns of geographical variation, fertility, and hybridization in Stuckenia (Potamogetonaceae)
Source: Front Plant Sci. 2022 Nov 3;13:1042517. doi: 10.3389/fpls.2022.1042517 (PMC9670304; doi:10.3389/fpls.2022.1042517)
Supplement: Supplementary file 2 [file Image_2.pdf]

Supplementary Figure 2 |
Placement of *5S-NTS* sequences from GenBank among species and genotypes of this study

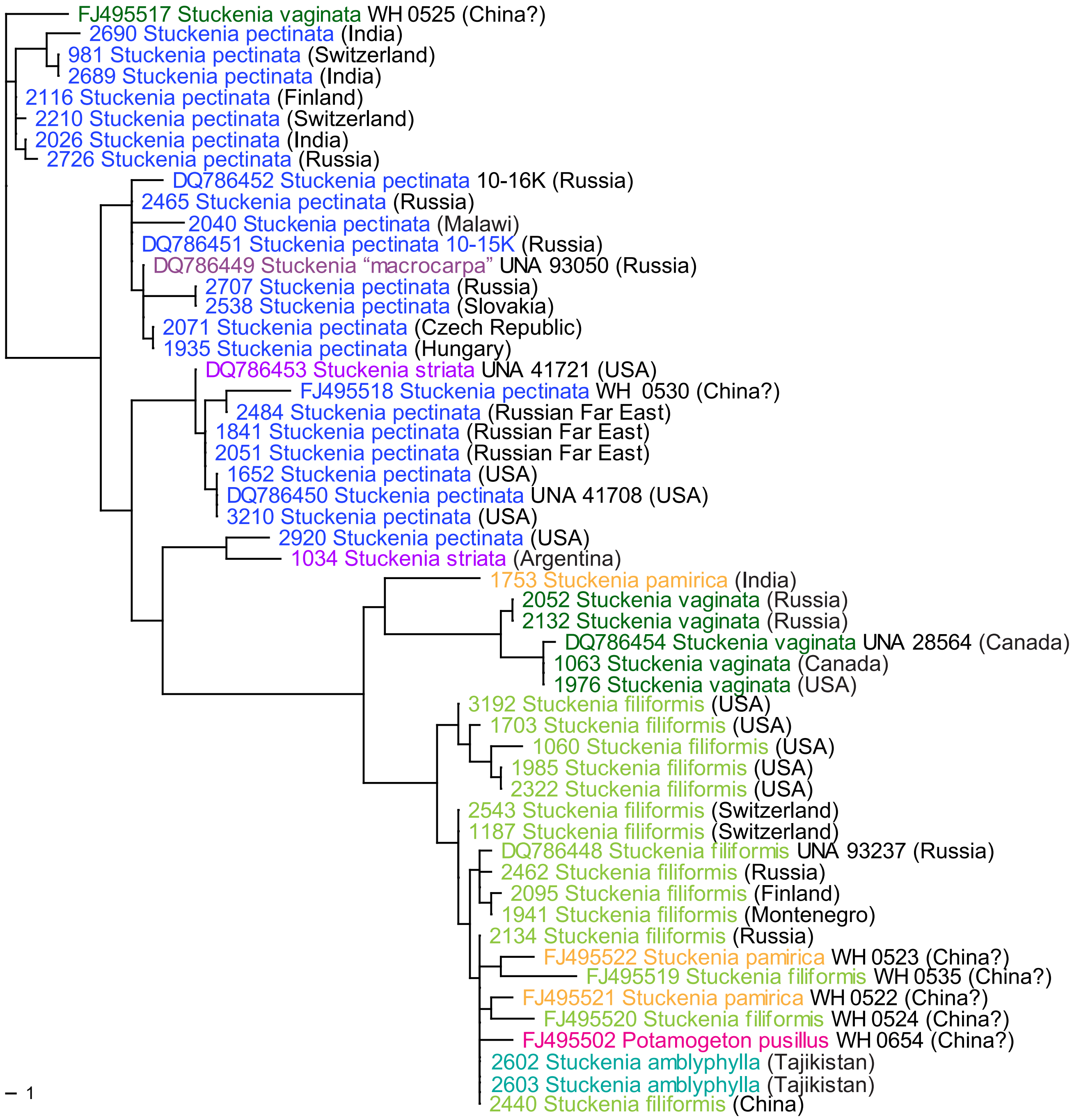

A Neighbor Joining tree shows the same dataset as in Figure 3 except that hybrids and some identical sequences were omitted. Additional sequences retrieved from GenBank with accession numbers starting with DQ are from Lindqvist et al. (2006), those starting with FJ are from Zhang and Wang (unpubl.). Sequences of the same nominal species are shown in matching colors; countries of origin are given for each sequence. The sample attributed to *Potamogeton pusillus* is different from that in Supplementary Figure 1 (*ITS*). Obviously or most likely misidentified samples are summarized in Table 1.
