# Supplementary material for: Evolutionary history and patterns of geographical variation, fertility, and hybridization in Stuckenia (Potamogetonaceae)
Source: Front Plant Sci. 2022 Nov 3;13:1042517. doi: 10.3389/fpls.2022.1042517 (PMC9670304; doi:10.3389/fpls.2022.1042517)
Supplement: Supplementary file 3 [file Image_3.pdf]

Supplementary Figure 3 |
Placement of *rpl*20-5'*rps*12 sequences from GenBank among species and genotypes of this study

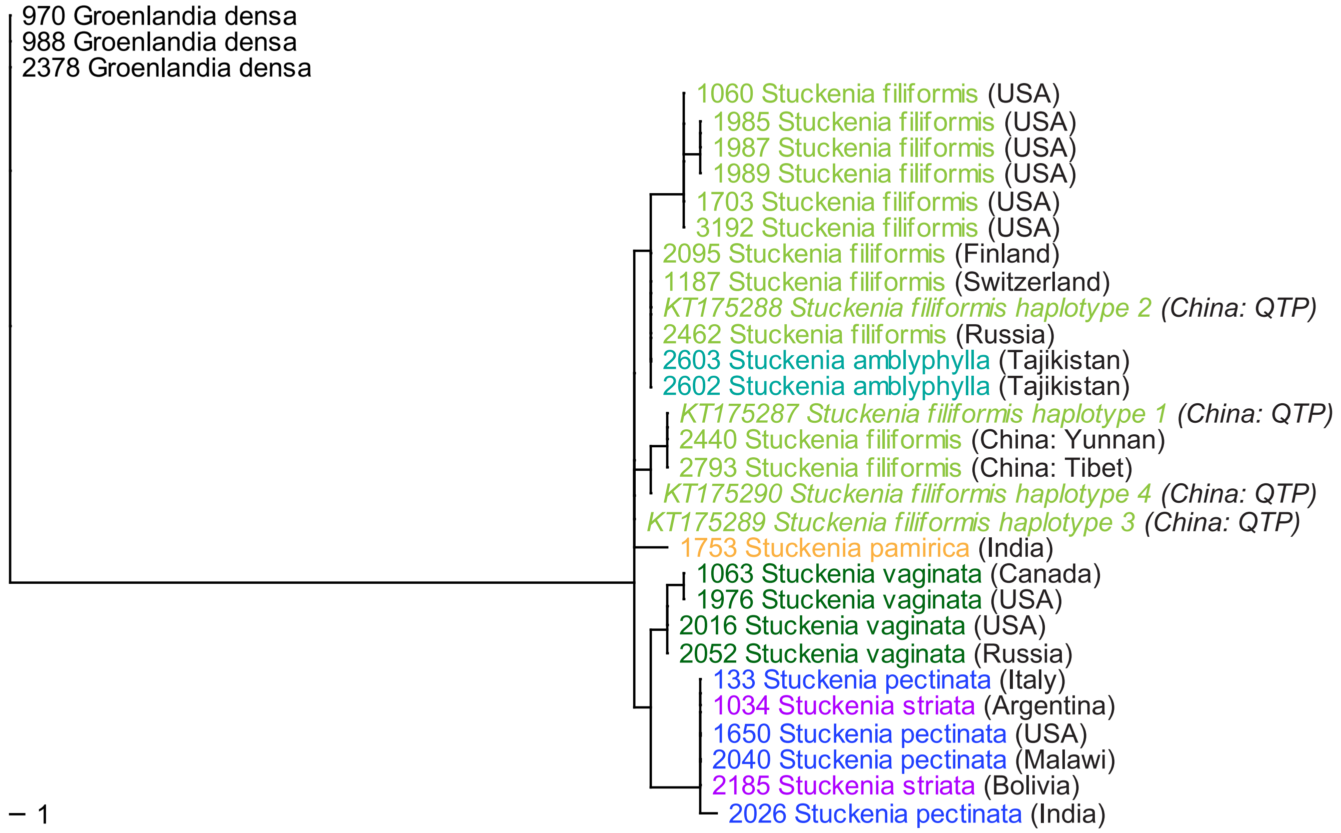

A Neighbor Joining tree shows the same dataset as in Figure 5 except that hybrids were omitted. Four haplotypes of *Stuckenia filiformis* from the Qinghai–Tibetan Plateau (QTP, Du and Wang 2016) are shown in italics. Colors of species correspond to Supplementary Figure 1; countries of origin are given for each sequence.
