# Supplementary material for: Evolutionary history and patterns of geographical variation, fertility, and hybridization in Stuckenia (Potamogetonaceae)
Source: Front Plant Sci. 2022 Nov 3;13:1042517. doi: 10.3389/fpls.2022.1042517 (PMC9670304; doi:10.3389/fpls.2022.1042517)
Supplement: Supplementary file 4 [file Image_4.pdf]

Supplementary Figure 4 |
Placement of *trn*T-*trn*L sequences from GenBank among species and genotypes of this study and origin of a misidentified sample of *Stuckenia* among species of *Potamogeton*

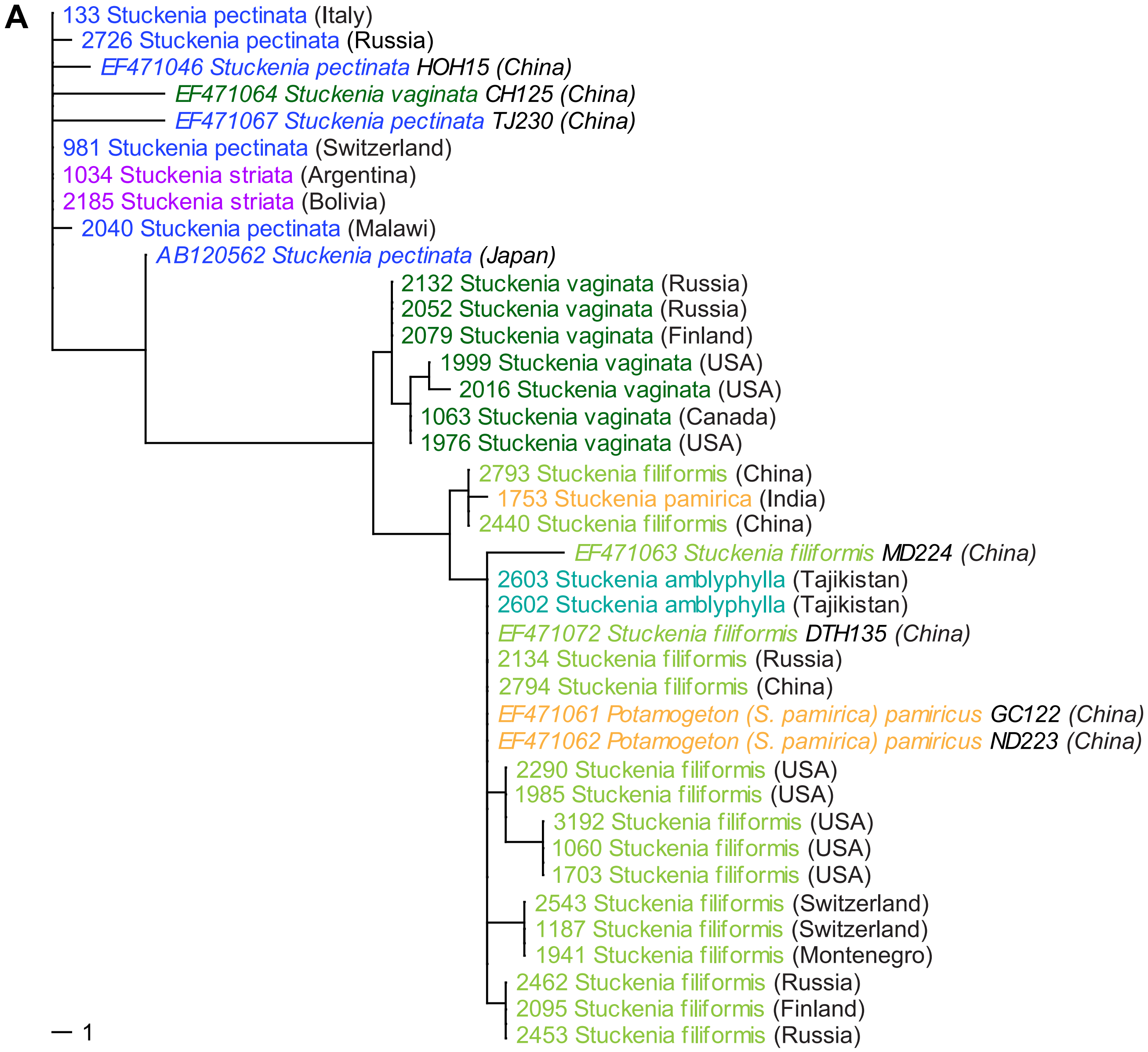

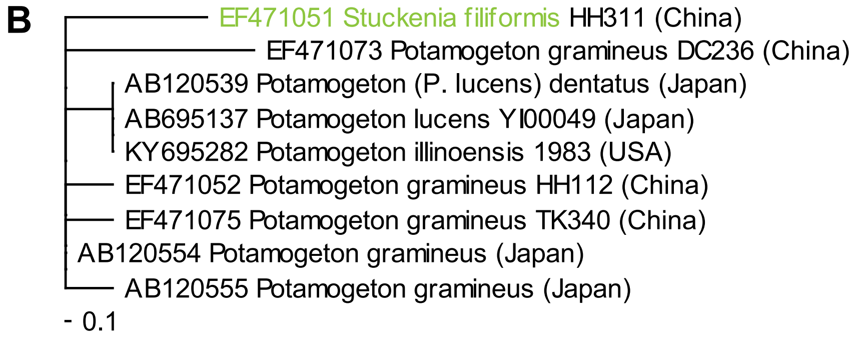

A) A Neighbor joining tree shows the same dataset as in Figure 6 except that hybrids were omitted. Seven haplotypes of *Stuckenia* from Zhang et al. (2008) and one from Iida et al. (2004) are shown in italics. Colors of species correspond to Supplementary Figures 1 and 3; countries of origin are given for each sequence. The correct name for *Potamogeton pamiricus* is *Stuckenia pamirica*. B) A Neighbor Joining tree shows one sample from Zhang et al. (2008), identified as *S. filiformis*, along with the most similar sequences of genus *Potamogeton*. *Potamogeton dentatus* is a synonym of *P. lucens.*
