# Supplementary material for: Evolutionary history and patterns of geographical variation, fertility, and hybridization in Stuckenia (Potamogetonaceae)
Source: Front Plant Sci. 2022 Nov 3;13:1042517. doi: 10.3389/fpls.2022.1042517 (PMC9670304; doi:10.3389/fpls.2022.1042517)
Supplement: Supplementary file 5 [file Table_1.pdf]

Supplementary Table 1 | List of samples, vouchers, chromosome numbers and GenBank accession numbers

Species / Hybrid	Sample / Identifier	Origin / Voucher	$2n$[1]	GenBank accession numbers			
				ITS	*rpl20-5'rps12*	*trnT-trnL*	*5S-NTS*
Groenlandia densa	970	Switzerland: canton Graubünden, Silvaplana, Champfèrer See, coll. *Z. Kaplan 98/70* (PRA)	30	KF270900	OP136177	OP136300	OP136383 –OP136389
	988	Austria: Vorarlberg, Bregenz, Hard, Lake Constance, coll. *Z. Kaplan 98/136* (PRA)	30	KF270901	OP136178	OP136301	OP136390 –OP136399
	2378	Slovakia: distr. Trnava, Dechtice, Blava stream, coll. *Z. Kaplan 11/301* (PRA)		OP101176	OP136179	OP136302	OP136400 –OP136408
Stuckenia amblyphylla	2602	Tajikistan: region Sughd, Zeravshan Mts, Allowodi valley, Allowdinske Lakes, coll. *A. S. Nowak*, pressed as *Z. Kaplan 2602* (PRA)		KF270922	OP136180	OP136303	OP136409 –OP136410
	2603	Tajikistan: region Sughd, Zeravshan Mts, Iskander-kul Lake, coll. *A. S. Nowak*, pressed as *Z. Kaplan 2603* (PRA)		KF270923	OP136181	OP136304	OP136411 –OP136412
S. filiformis	1060	USA: Maine, Aroostook Co., Presque Isle, coll. *C. B. Hellquist*, cult. & coll. *Z. Kaplan 1060* (PRA)		OP101177	OP136182	OP136305	OP136413 –OP136414
	1187	Switzerland: canton Sankt Gallen, Rorschach, Lake Constance, cult. & coll. *Z. Kaplan 1187* (PRA)	78	KF270924	OP136183	OP136306	OP136415 –OP136416
	1703	USA: Maine, Aroostook Co., New Limerick, Nickerson Lake, coll. *Z. Kaplan & C. B. Hellquist 05/426* (PRA)	78	KF270925	OP136184	OP136307	OP136417 –OP136418
	1941	Montenegro: distr. Plav, Plav, Lake Plav, coll. *Z. Kaplan 08/560* (PRA)		OP101178	OP136185	OP136308	OP136419 –OP136420
	1985	USA: Wyoming, Park Co., Mammoth, Joffie Lake, coll. *C. E. Hellquist et al. 73-08* (PRA)		OP101179	OP136186	OP136309	OP136421 –OP136422
	1987	USA: Wyoming, Park Co., Norris, Gibbon River, coll. *C. E. Hellquist 92-08 & C. B. Hellquist* (PRA)		OP101180	OP136187	OP136310	OP136423 –OP136424
	1989	USA: Wyoming, Park Co., stream feeding Obsidian Creek, coll. *C. E. Hellquist 137-08 & C. B. Hellquist* (PRA)		OP101181	OP136188		
	1992	USA: Wyoming, Park Co., Obsidian Creek, coll. *C. E. Hellquist 191-08 & C. B. Hellquist* (PRA)		OP101182	OP136189	OP136311	
	2006	USA: Wyoming, Teton Co., Firehole River, coll. *C. E. Hellquist 406-08 & C. B. Hellquist* (PRA)		OP101183	OP136190		
	2095	Finland: North Ostrobothnia, Raahe, Aunolanperä, Gulf of Bothnia, Mitti island, coll. *Z. Kaplan et al. 09/306* (PRA)		OP101184	OP136191	OP136312	OP136425 –OP136431
	2108	Finland: South Häme, Hollola, Lake Vesijärvi, coll. *Z. Kaplan & P. Uotila 09/317* (PRA)		OP101185	OP136192		
	2134	Russia: Siberia, prov. Irkutsk, distr. Cheremkhovskii, Russkaya Alar', Verkhnaya Iret' River, coll. *Z. Kaplan & V. Chepinoga 09/360* (PRA)	78	OP101186	OP136193	OP136313	OP136432 –OP136433

2288	USA: Wyoming, Park Co., Mammoth – Norris, Winter Creek, coll. *C. E. Hellquist 619-10 & C. B. Hellquist* (PRA)		OP101187	OP136194		
2290	USA: Wyoming, Park Co., Obsidian Creek, coll. *C. E. Hellquist 626-10 & C. B. Hellquist* (PRA)		OP101188	OP136195	OP136314	OP136434 –OP136435
2291	USA: Wyoming, Teton Co., Fairy Creek, coll. *C. E. Hellquist 642-10 & C. B. Hellquist* (PRA)		OP101189	OP136196	OP136315	
2296	USA: Wyoming, Teton Co., Sentinel Creek, coll. *C. E. Hellquist 735-10 & C. B. Hellquist* (PRA)		OP101190			
2297	USA: Wyoming, Teton Co., stream feeding Sentinel Creek, coll. *C. E. Hellquist 747-10 & C. B. Hellquist* (PRA)		OP101191			
2298	USA: Wyoming, Teton Co., Firehole River, coll. *C. E. Hellquist 748-10 & C. B. Hellquist* (PRA)		OP101192			
2322	USA: Wyoming, Park Co., Norris – Madison, Gibbon River, coll. *C. E. Hellquist 1087-10 & C. B. Hellquist* (PRA)		OP101193	OP136197	OP136316	OP136436 –OP136437
2440	China: prov. Yunnan, Diqing Pref., Zhongdian Co., Zhongdian, coll. *B. Aldén et al. 1407* (E)		OP101194	OP136198	OP136317	OP136438 –OP136439
2453	Russia: European part, Komi Rep., Vorkuta, Severnyi, Ngayats'yakha River, coll. *A. A. Bobrov & D. A. Philippov 09-120* (PRA, IBIW)		OP101195	OP136199	OP136318	
2461	Russia: European part, prov. Arkhangelsk, distr. Kholmogorskii, Novaya Il'ma, Vaimuga River, coll. *A. A. Bobrov et al. 09-89* (PRA)		OP101196			
2462	Russia: European part, prov. Arkhangelsk, distr. Ust'yanskii, Schapinskaya, Volyuga River, coll. *A. A. Bobrov et al. 10-37* (PRA)		OP101197	OP136200	OP136319	OP136440 –OP136444
2463	Russia: European part, prov. Arkhangelsk, distr. Pinezhskii, Shirokoe, Yozhuga River, coll. *A. A. Bobrov et al. 10-87* (PRA)		OP101198			
2464	Russia: European part, prov. Arkhangelsk, distr. Pinezhskii, Nyukhcha, Nyukhcha River, coll. *A. A. Bobrov et al. 10-104* (PRA)		OP101199			
2543	Switzerland: canton Sankt Gallen, Altstätten, Kriessern, coll. *Z. Kaplan 12/195* (PRA)	78	OP101200	OP136201	OP136320	OP136445 –OP136446
2793	China: prov. Tibet, Deqin, Ninzhong, coll. *J.-M. Chen 4* (PRA)		OP101201	OP136202	OP136321	
2794	China: prov. Sichuan, Ganzi, Litang, coll. *Z.-Y. Du 5* (PRA)		OP101202	OP136203	OP136322	OP136447 –OP136448
3192	USA: Colorado, Summit Co., Copper Mountain, Curtain Ponds, coll. *M. Majack*, pressed as *Z. Kaplan 3192* (PRA)		OP101203	OP136204	OP136323	OP136449 –OP136455
3216	USA: Wyoming, Teton Co., Snake River, coll. *C. E. Hellquist 1184-14 & C. B. Hellquist* (PRA)		OP101204			
3217	USA: Wyoming, Teton Co., Blacktail Ponds, coll. *C. E. Hellquist 1187-14 & C. B. Hellquist* (PRA)		OP101205			

	3218	USA: Wyoming, Teton Co., Northern Springs, coll. *C. E. Hellquist 1196-14 & C. B. Hellquist* (PRA)		OP101206			
	3229	USA: Wyoming, Teton Co., Schwabackers Landing, coll. *C. E. Hellquist 1366-14 & C. B. Hellquist* (PRA)		OP101207			
	3248	USA: Wyoming, Teton Co., Elk Ranch Reservoir, coll. *C. E. Hellquist 1430-14 & C. B. Hellquist* (PRA)		OP101208			
	3252	USA: Wyoming, Teton Co., Christian Pond, coll. *C. E. Hellquist 1493-14 & C. B. Hellquist* (PRA)		OP101209			
S. pamirica	1753	India: Jammu & Kashmir, Ladakh, Rupshu, Samad Rokchen, Startsa Puk Tso, coll. *L. Klimeš 6276* (PRA)		OP101210	OP136205	OP136324	OP136456 –OP136457
S. pectinata	133	Italy: Friuli-Venezia Giulia, prov. Udine, Cervignano del Friuli, Ausa River, coll. *Z. Kaplan 96/21* (PRA)		OP101211	OP136206	OP136325	OP136458 –OP136459
	981	Switzerland: canton Sankt Gallen, Rorschach, Altenrhein, coll. *Z. Kaplan 98/127* (PRA)	78	OP101212	OP136207	OP136326	OP136460 –OP136461
	989	Switzerland: canton Thurgau, Ermatingen, Untersee, coll. *Z. Kaplan 98/140* (PRA)		OP101213	OP136208		
	1010	Sweden: prov. Södermanland, Dalarö, coll. *Z. Kaplan*, cult. & coll. *Z. Kaplan 1010* (PRA)		OP101214	OP136209		
	1023	Denmark: Jutland, region Midtjylland, Langå, Gudenå River, coll. *Z. Kaplan 98/373* (PRA)		OP101215	OP136210	OP136327	
	1650	USA: Vermont, Addison Co., Weybridge, Otter Creek, coll. *Z. Kaplan & C. B. Hellquist 05/383* (PRA)	78	KF270926	OP136211	OP136328	
	1652	USA: Vermont, Addison Co., Weybridge, Otter Creek, coll. *Z. Kaplan & C. B. Hellquist 05/385* (PRA)		OP101216	OP136212	OP136329	OP136462 –OP136463
	1711	USA: Maine, Aroostook Co., Mars Hill, pond on Prestile Stream, coll. *Z. Kaplan & C. B. Hellquist 05/436* (PRA)		OP101217	OP136213		
	1837	Russia: Siberia, prov. Irkutsk, distr. Alarskii/Cheremkhovskii, Alar', Nygda, Golumet' River, coll. *V. Chepinoga*, cult. & coll. *Z. Kaplan 1837* (PRA)	78	OP101218	OP136214	OP136330	
	1841	Russia: Siberia, prov. Irkutsk, distr. Alarskii, Kapsal, Kuda River, coll. *V. Chepinoga*, cult. & coll. *Z. Kaplan 1841* (PRA)	78	OP101219	OP136215	OP136331	OP136464 –OP136465
	1869	USA: Michigan, Presque Isle Co., Cheboygan, Qcqueoc River, coll. *C. B. Hellquist 16976* (PRA)		OP101220	OP136216	OP136332	OP136466 –OP136467
	1935	Hungary: Somogy county, Balatonboglár, Szabadság-telep, coll. *Z. Dočkalová*, cult. & coll. *Z. Kaplan 1935* (PRA)		OP101221	OP136217	OP136333	OP136468 –OP136469
	2026	India: Kashmir, distr. Bandipora, Naninara, Manasbal Lake, coll. *A. H. Ganie*, cult. & coll. *Z. Kaplan 2026* (PRA)	78	KF270927	OP136218	OP136334	OP136470 –OP136471

2040	Malawi: Lake Malawi, Likoma Island, coll. *C. Kasselmann 135* (B)	OP101222	OP136219	OP136335	OP136472 –OP136473
2051	Russia: Siberia, prov. Irkutsk, distr. Ziminskii, Batama, coll. *S. Rosbakh & K. Fleckenstein*, cult. & coll. *Z. Kaplan 2051* (PRA)	OP101223	OP136220	OP136336	OP136474 –OP136475
2071	Czech Republic: distr. Rokycany, Ejpovice, coll. *Z. Kaplan 09/87* (PRA)	OP101224	OP136221	OP136337	OP136476 –OP136477
2090	Finland: North Ostrobothnia, Raahe, Lake Aittolahti, coll. *Z. Kaplan et al. 09/301* (PRA)	OP101225	OP136222		
2116	Finland: Uusimaa, Espoo, Tanskarla, Långsvik, coll. *Z. Kaplan & P. Uotila 09/324* (PRA)	OP101226	OP136223	OP136338	OP136478 –OP136479
2210	Switzerland: canton Thurgau, Ermatingen, Untersee, coll. *E. Gross*, cult. & coll. *Z. Kaplan 2210* (PRA)	OP101227	OP136224	OP136339	OP136480 –OP136481
2211	Switzerland: canton Thurgau, Ermatingen, Untersee, coll. *E. Gross*, cult. & coll. *Z. Kaplan 2211* (PRA)	OP101228			
2212	Switzerland: canton Thurgau, Ermatingen, Untersee, coll. *E. Gross*, cult. & coll. *Z. Kaplan 2212* (PRA)	OP101229			
2213	Switzerland: canton Thurgau, Ermatingen, Untersee, coll. *E. Gross*, cult. & coll. *Z. Kaplan 2213* (PRA)	OP101230			
2228	USA: New York, Seneca Co., Canoga, Cayuga Lake, Canoga Marsh, coll. *C. B. Hellquist et al. 17124* (PRA)	OP101231 –OP101232	OP136225		
2283	USA: Texas, Reeves Co., Balorhea, coll. *C. B. Hellquist 17189* (PRA)	OP101233 –OP101234	OP136226	OP136340	OP136482 –OP136483
2448	Russia: European part, prov. Arkhangelsk, distr. Verkhnetoemskii, Uschazh, Yorga River, coll. *A. A. Bobrov et al. 09-39* (PRA)	OP101235	OP136227	OP136341	OP136484 –OP136485
2465	Russia: European part, prov. Arkhangelsk, distr. Kholmogorii, Vakhnovo, Pin'gisha River, coll. *A. A. Bobrov et al. 10-80* (PRA)	OP101236	OP136228	OP136342	OP136486 –OP136487
2484	Russia: Far East, prov. Magadan, distr. Srednekanskii, Karasyovoe Lake, coll. *O. Mochalova 10-149* (PRA)	OP101237	OP136229	OP136343	OP136488 –OP136489
2485	Russia: Far East, prov. Magadan, distr. Srednekanskii, Karasyovoe Lake, coll. *O. Mochalova 10-150* (PRA)	OP101238			
2486	Russia: Far East, prov. Magadan, distr. Srednekanskii, Karasyovoe Lake, coll. *O. Mochalova 10-151* (PRA)	OP101239			
2487	Russia: Far East, prov. Magadan, distr. Srednekanskii, Kolyma River, coll. *O. Mochalova 10-152* (PRA)	OP101240			
2488	Russia: Far East, prov. Magadan, distr. Srednekanskii, Kolyma River, coll. *O. Mochalova 10-153* (PRA)	OP101241			
2538	Slovakia: distr. Nitra, Malý Cetín, coll. *Z. Kaplan 12/188* (PRA)	OP101242	OP136230	OP136344	OP136490 –OP136491

2541	Slovakia: distr. Trnava, Zavar, coll. *Z. Kaplan 12/193* (PRA)	OP101243			
2545	Switzerland: canton Schwyz, Tuggen, Schloss Grynau, coll. *Z. Kaplan 12/197* (PRA)	OP101244			
2561	Denmark: Jutland, region Syddanmark, Hajstrup, Grønå stream, coll. *Z. Kaplan & J. Prančl 12/211* (PRA)	OP101245	OP136231	OP136345	OP136492 –OP136493
2586	Denmark: Jutland, region Midtjylland, Langå, Gudenå River, coll. *Z. Kaplan et al. 12/235* (PRA)	OP101246			
2587	Denmark: Jutland, region Midtjylland, Langå, Gudenå River, coll. *Z. Kaplan et al. 12/236* (PRA)	OP101247			
2589	Denmark: Jutland, region Midtjylland, Langå, Gudenå River, coll. *Z. Kaplan et al. 12/238* (PRA)	OP101248			
2644	USA: Michigan, Cheboygan Co., Douglas Lake, Marl Bay, mouth of Bessey Creek, coll. *C. E. Hellquist 3028* (PRA)	OP101249			
2689	India: Kashmir, distr. Srinagar, Srinagar, Dal Lake, coll. *A. H. Ganie 10077* (PRA)	OP101250	OP136232	OP136346	OP136494 –OP136495
2690	India: Kashmir, distr. Anantnag, Brakpora, Nambal rivulet, coll. *A. H. Ganie 10078* (PRA)	OP101251	OP136233	OP136347	OP136496 –OP136497
2694	Kazakhstan: Uralsk, distr. Urdinsky, Sayhin, coll. *G. U. Klinkova & I. A. Schanzer 1990-01-02* (PRA)	OP101252			
2695	Russia: European part, prov. Volgograd, distr. Chernyshkovskii, Morskoi, coll. *G. U. Klinkova 1991-01-01* (PRA)	OP101253			
2696	Russia: European part, prov. Volgograd, distr. Chernyshkovskii, Morskoi, coll. *G. U. Klinkova 1991-02-01* (PRA)	OP101254			
2697	Russia: European part, prov. Volgograd, distr. Chernyshkovskii, Morskoi, coll. *G. U. Klinkova 1991-02-02* (PRA)	OP101255			
2698	Russia: European part, prov. Volgograd, distr. Chernyshkovskii, Morskoii, coll. *G. U. Klinkova 1991-01-02* (PRA)	OP101256			
2699	Russia: European part, prov. Volgograd, distr. Chernyshkovskii, Morskoii, coll. *G. U. Klinkova 1991-01-03* (herb. Univ. Volgograd)	OP101257			
2705	Russia: European part, prov. Volgograd, distr. Pallasovskii, Lake Elton, Smorogda River, coll. *G. U. Klinkova 2003-03-01* (PRA)	OP101258		OP136348	OP136498 –OP136499
2706	Russia: European part, prov. Volgograd, distr. Pallasovskii, Lake Elton, Smorogda River, coll. *G. U. Klinkova 2003-03-02* (PRA)	OP101259			
2707	Russia: European part, prov. Volgograd, distr. Pallasovskii, Lake Elton, Smorogda River, coll. *G. U. Klinkova 2003-03-03* (PRA)	OP101260	OP136234	OP136349	OP136500 –OP136503

	2708	Russia: European part, prov. Volgograd, distr. Pallasovskii, Lake Elton, Smorogda River, coll. *G. U. Klinkova 2003-03-04* (PRA)		OP101261			
	2724	Russia: European part, prov. Volgograd, distr. Svetloyarskii, Lake Sarpa, coll. *G. U. Klinkova 2006-08-01* (PRA)		OP101262			
	2725	Russia: European part, prov. Volgograd, distr. Svetloyarskii, Lake Sarpa, coll. *G. U. Klinkova 2006-08-02* (PRA)		OP101263			
	2726	Russia: European part, prov. Volgograd, distr. Svetloyarskii, Lake Sarpa, coll. *G. U. Klinkova 2006-08-03* (PRA)		OP101264	OP136235	OP136350	OP136504 –OP136505
	2795	China: prov. Xinjiang, Yanqi, Xiangsihu, coll. *J.-M. Chen 6* (PRA)		OP101265			
	2796	China: prov. Sichuan, Ganzi, Luhuo, Kashahu, coll. *Z.-Y. Du 7* (PRA)		OP101266	OP136236	OP136351	
	2797	China: prov. Inner Mongolia, Manzhouli, Hulun Lake, coll. *Z.-Y. Du 8* (PRA)		OP101267	OP136237	OP136352	OP136506 –OP136507
	2920	USA: Vermont, Windsor Co., Windsor, Lake Runnemede, coll. *Z. Kaplan & C. B. Hellquist 13/271* (PRA)		OP101268	OP136238	OP136353	OP136508 –OP136509
	3201	Turkey: prov. Antalya, Düden, coll. *C. Aykurt 4038* (PRA)		OP101269	OP136239	OP136354	OP136510 –OP136511
	3210	USA: Utah, Box Elder Co., Bear River Migratory Bird Refuge, coll. *C. B. Hellquist 17384 & C. E. Hellquist* (PRA)		OP101270 –OP101271	OP136240	OP136355	OP136512 –OP136513
	3225	USA: Wyoming, Teton Co., Polecat Creek, coll. *C. E. Hellquist 1268-14 & C. B. Hellquist* (PRA)		OP101272 –OP101273		OP136356	OP136514 –OP136515
S. striata	1034	Argentina: prov. Buenos Aires, San Miguel del Monte, coll. *M. Zaleski*, cult. & coll. *Z. Kaplan 1034* (PRA)		OP101274	OP136241	OP136357	OP136516 –OP136517
	2185	Bolivia: dept. Cochabamba, prov. Arani, Lake Juntutuyu, coll. *N. Ritter et al. 2116* (W)		OP101275	OP136242	OP136358	
	3029	Peru: Mollendo, Mejia Lagoons, coll. *E. T. W. Haig 23.1* (K)		OP101276			
S. vaginata	1063	Canada: Manitoba, town of Snow Lake, Tramping Lake, coll. *C. B. Hellquist & C. E. Hellquist*, cult. & coll. *Z. Kaplan 1063* (PRA)	78	OP101277	OP136243	OP136359	OP136518 –OP136519
	1919	Finland: Ostrobothnia, Mustasaari, Koivulahti, Värlax, Önskäret, coll. *J. Suominen 15677* (H)		OP101278			
	1976	USA: Montana, Park Co., Emigrant, Dailey Lake, coll. *C. B. Hellquist 17094 & C. E. Hellquist* (PRA)		KF270928	OP136244	OP136360	OP136520 –OP136521
	1999	USA: Montana, Park Co., Slide Lake, coll. *C. E. Hellquist 285-08 & C. B. Hellquist* (PRA)		OP101279	OP136245	OP136361	OP136522 –OP136523
	2016	USA: Wyoming, Wyoming, Park Co.,Soda Butte, Foster Lake, coll. *C. E. Hellquist 579-08 & C. B. Hellquist* (PRA)		OP101280	OP136246	OP136362	OP136524 –OP136525

	2052	Russia: Siberia, prov. Zabaikalskii, distr. Uletovskii, Tanga, coll. *S. Rosbakh & K. Fleckenstein*, cult. & coll. *Z. Kaplan 2052* (PRA)	78	OP101281	OP136247	OP136363	OP136526 –OP136527
	2097	Finland: North Ostrobothnia, Raahe, Aunolanperä, Gulf of Bothnia, Mitti island, coll. *Z. Kaplan et al. 09/308* (PRA)		OP101282	OP136248	OP136364	OP136528 –OP136529
	2132	Russia: Siberia, prov. Irkutsk, distr. Usol'skii, Usol'e-Sibirskoe, coll. *Z. Kaplan & V. Chepinoga 09/358* (PRA)		OP101283	OP136249	OP136365	OP136530 –OP136531
S. amblyphylla × *S. filiformis*	2183	India: Kashmir, distr. Anantnag, Sandoo, Nagrad stream, coll. *A. H. Ganie 100122* (PRA)		OP101284	OP136250	OP136366	
	2666	India: Kashmir, distr. Anantnag, Sandoo, Nagrad stream, coll. *A. H. Ganie 100122* (PRA)		OP101285	OP136251	OP136367	
	2789	Kazakhstan: valley of Tekes River, Kakpak, coll. *J. Štěpánek 438* (PRA)		OP101286	OP136252	OP136368	
	3258	India: Jammu & Kashmir, Shey, Thikse, Indus River, coll. *P. Koutecký s. n.* (PRA)		OP101287	OP136253	OP136369	
S. pectinata × *S. filiformis* (*S.* × *suecica*)	1009	Sweden: prov. Södermanland, Dalarö, coll. *Z. Kaplan*, cult. & coll. *Z. Kaplan 1009* (PRA)	78	OP101288	OP136254		
	1993	USA: Wyoming, Park Co., Norris – Mammoth Jct., coll. *C. E. Hellquist 198-08 & C. B. Hellquist* (PRA)		OP101292 –OP101293	OP136257		
	1995	USA: Wyoming, Teton Co., Snake River, coll. *C. E. Hellquist 260-08 & C. B. Hellquist* (PRA)		OP101294 –OP101295	OP136258		
	1996	USA: Wyoming, Teton Co., Snake River, coll. *C. E. Hellquist 263-08 & C. B. Hellquist* (PRA)		OP101296	OP136259		
	1998	USA: Wyoming, Teton Co., Snake River, coll. *C. E. Hellquist 272-08 & C. B. Hellquist* (PRA)		OP101297	OP136260		
	2002	USA: Wyoming, Wyoming, Teton Co., Firehole River, coll. *C. E. Hellquist 344-08 & C. B. Hellquist* (PRA)		OP101298 –OP101299	OP136261		
	2003	USA: Wyoming, Park Co., Norris – Madison, Gibbon River, coll. *C. E. Hellquist 360-08 & C. B. Hellquist* (PRA)		OP101300 –OP101301	OP136262		
	2004	USA: Wyoming, Teton Co., Firehole River, coll. *C. E. Hellquist 402-08 & C. B. Hellquist* (PRA)		OP101302 –OP101306	OP136263		
	2010	USA: Wyoming, Teton Co., Hayden Valley, Alum Creek, coll. *C. E. Hellquist 477-08 & C. B. Hellquist* (PRA)		OP101307 –OP101308	OP136264		
	2168	Germany: Mecklenburg-Vorpommern, Feldberg, Lake Breiter Luzin, coll. *Z. Kaplan 09/341* (PRA)		OP101289	OP136255		
	2253	Germany: Rheinland-Pfalz, Glees, Laacher Lake, coll. *K. van de Weyer*, cult. & coll. *Z. Kaplan 2253* (PRA)		OP101290 –OP101291	OP136256		
	2287	USA: Wyoming, Park Co., Mammoth – Norris, coll. *C. E. Hellquist 614-10 & C. B. Hellquist* (PRA)		OP101309 –OP101310	OP136265		
	2293	USA: Wyoming, Teton Co., Fountain Flats, coll. *C. E. Hellquist 648-10 & C. B. Hellquist* (PRA)		OP101311 –OP101312	OP136266		

	2294	USA: Wyoming, Teton Co., Lower Geyser Basin,, coll. *C. E. Hellquist 657-10 & C. B. Hellquist* (PRA)		OP101313 –OP101314	OP136267	
	2303	USA: Wyoming, Park Co., Norris, Nymph Lake, coll. *C. E. Hellquist 866-10 & C. B. Hellquist* (PRA)		OP101315 –OP101316	OP136268	
	2314	USA: Wyoming, Teton Co., Lewis Lake, coll. *C. E. Hellquist 1002-10 & C. B. Hellquist* (PRA)		OP101317 –OP101319	OP136269	
	2321	USA: Wyoming, Park Co., Hayden Valley, Yellowstone River, coll. *C. E. Hellquist 1077-10 & C. B. Hellquist* (PRA)		OP101320 –OP101321	OP136270	
	2327	USA: Wyoming, Park Co., Pelican Creek, coll. *C. E. Hellquist 1104-10 & V. Xuan* (PRA)		OP101322 –OP101323	OP136271	
	3223	USA: Wyoming, Teton Co., Polecat Creek, coll. *C. E. Hellquist 1256-14 & C. B. Hellquist* (PRA)		OP101324 –OP101325	OP136272	
	3226	USA: Wyoming, Teton Co., Polecat Creek, coll. *C. E. Hellquist 1280-14 & C. B. Hellquist* (PRA)		OP101326 –OP101327	OP136273	
S. filiformis × *S. vaginata* (*S.* × *fennica*)	1651	USA: Vermont, Addison Co., Weybridge, Otter Creek, coll. *Z. Kaplan & C. B. Hellquist 05/384* (PRA)	78	OP101331	OP136275	
	1710	USA: Maine, Aroostook Co., Blaine, Prestile Stream, coll. *Z. Kaplan & C. B. Hellquist 05/435* (PRA)	78	OP101332	OP136276	
	1877	USA: Michigan, Emmet Co., Maple River, coll. *C. B. Hellquist 16984* (PRA)		OP101333	OP136277	
	1878	USA: Michigan, Cheboygan Co., Sturgeon River, coll. *C. B. Hellquist 16985* (PRA)		OP101334	OP136278	
	1879	USA: Michigan, Cheboygan Co., Cheboygan, Elliot Creek, coll. *C. B. Hellquist 16986* (PRA)		OP101335	OP136279	
	1980	USA: Michigan, Cheboygan Co., Cheboygan, Elliot Creek, coll. *C. B. Hellquist 17099* (PRA)		OP101336	OP136280	
	1991	USA: Wyoming, Park Co., Obsidian Creek, coll. *C. E. Hellquist 186-08 & C. B. Hellquist* (PRA)		OP101337	OP136281	
	2141	Russia: Siberia, prov. Irkutsk, distr. Ziminskii, Ignai, Zima River, coll. *Z. Kaplan & V. Chepinoga 09/367* (PRA)	78	KF270929	OP136285	OP136375
	2446	Russia: European part, Komi Rep., Sovetskii, Usa River, coll. *A. A. Bobrov & D. A. Philippov 09-127* (PRA)		OP101339	OP136283	OP136373
	2452	Russia: European part, Komi Rep., Tsementnozavodskii, Vorkuta River, coll. *A. A. Bobrov & D. A. Philippov 09-117* (PRA)		OP101340 –OP101342	OP136284	OP136374
	2454	Russia: European part, Komi Rep., Vorkuta, Vorkuta River, coll. *A. A. Bobrov & D. A. Philippov 09-124 p. p.* (PRA)		OP101328 –OP101329		OP136370
	2455	Russia: European part, Komi Rep., Vorkuta, Vorkuta River, coll. *A. A. Bobrov & D. A. Philippov 09-130* (IBIW)		OP101330	OP136274	OP136371

	2456	Russia: European part, prov. Arkhangelsk, distr. Vinogradovskii, Kvakhtyuga, Vaen'ga River, coll. *A. A. Bobrov et al. 09-52* (PRA)		OP101343		OP136376
	2457	Russia: European part, prov. Arkhangelsk, distr. Plesetskii, Savinskii, Emtsa River, coll. *A. A. Bobrov et al. 09-91* (PRA)		OP101344		OP136377
	2458	Russia: European part, prov. Vologda, distr. Totemskii, Tot'ma, Eden'ga River, coll. *A. A. Bobrov et al. 09-8* (PRA)		OP101345		OP136378
	2459	Russia: European part, prov. Vologda, distr. Tarnogskii, Krotovskaya, Uftyuga River, coll. *A. A. Bobrov et al. 09-15* (PRA)		OP101346		OP136379
	2460	Russia: European part, prov. Vologda, distr. Nyuksenskii, Dvorische, Gorodischna River, coll. *A. A. Bobrov et al. 09-19* (IBIW)		OP101347		OP136380
	2467	Russia: European part, prov. Arkhangelsk, distr. Pinezhskii, Shirokoe, Yozhuga River, coll. *A. A. Bobrov et al. 10-90* (PRA)		OP101348		OP136381
	2652	Canada: Newfoundland, Northern Peninsula, Hawkes Bay, coll. *C. B. Hellquist 17251* (PRA)		OP101338	OP136282	OP136372
S. pectinata × *S. vaginata* (*S.* × *bottnica*)	1027	Denmark: Jutland, region Syddanmark, Rens, Sonderå stream, coll. *Z. Kaplan 98/379* (PRA)	78	OP101349 –OP101350	OP136286	
	1840	Russia: Siberia, prov. Irkutsk, distr. Alarskiy, Alar', Nygda, Golumet' River, coll. *V. Chepinoga*, cult. & coll. *Z. Kaplan 1840* (PRA)	78	OP101351 –OP101352	OP136287	
	1868	USA: Michigan, Cheboygan Co., Myers Creek, coll. *C. B. Hellquist 16975* (PRA)		OP101364 –OP101365	OP136294	
	1870	USA: Michigan, Presque Isle Co., Cheboygan, Qcqueoc River, coll. *C. B. Hellquist 16977* (PRA)		OP101366 –OP101367	OP136295	
	1875	USA: Michigan, Cheboygan Co., Burt Lake, Maple Bay, coll. *C. B. Hellquist 16982* (PRA)		OP101368 –OP101369	OP136296	
	1978	USA: Michigan, Cheboygan Co., Myers Creek, coll. *C. B. Hellquist 17098 & C. E. Hellquist* (PRA)		OP101370 –OP101371	OP136297	
	2087	Finland: North Ostrobothnia, Raahe, Lapaluoto, coll. *Z. Kaplan et al. 09/298* (PRA)		OP101353 –OP101354	OP136288	
	2088	Finland: North Ostrobothnia, Raahe, Lapaluoto, coll. *Z. Kaplan et al. 09/299* (PRA)		OP101355 –OP101356	OP136289	
	2136	Russia: Siberia, prov. Irkutsk, distr. Alarskii/Cheremkhovskii, Alar', Nygda, Golumet' River, coll. *Z. Kaplan & V. Chepinoga 09/362* (PRA)		OP101357 –OP101358	OP136290	
	2466	Russia: European part, prov. Arkhangelsk, distr. Leshukonskii, Uschel'e, Yozhuga River, coll. *A. A. Bobrov et al. 10-117* (PRA)		OP101359 –OP101360	OP136291	
	2555	Denmark: Jutland, region Syddanmark, Rens, Sonderå stream, coll. *Z. Kaplan & J. Prančl 12/205* (PRA)		OP101361 – OP101362	OP136292	

	2556	Denmark: Jutland, region Syddanmark, Saksborg, Grønå stream, coll. *Z. Kaplan & J. Prančl 12/206* (PRA)		OP101363	OP136293		
	3075	USA: Maine, Penobscot Co., Bangor, Kenduskeag River, coll. *C. B. Hellquist 17308* (PRA)		OP101372 –OP101373	OP136298		
S. striata × *S.* sp.	855	Argentina: prov. Buenos Aires, Bahía Blanca, cult. at Delft University, Netherlands, cult. & coll. *Z. Kaplan 855* (PRA)	78	OP101374 –OP101375	OP136299	OP136382	OP136532 –OP136533

[1] chromosome numbers from Kaplan et al. (2013)
