# Supplementary material for: Evolutionary history and patterns of geographical variation, fertility, and hybridization in Stuckenia (Potamogetonaceae)
Source: Front Plant Sci. 2022 Nov 3;13:1042517. doi: 10.3389/fpls.2022.1042517 (PMC9670304; doi:10.3389/fpls.2022.1042517)
Supplement: Supplementary file 6 [file Table_2.pdf]

Supplementary Table 2 | Intra- and interspecific variation of closely related species, number of intra-individual polymorphic sites (*ITS*)

A) ***Stuckenia pectinata*** (genotypes 1a–b, 2a–e, and intraspecific hybrids), ***S. striata*, and *S. striata* × *S.* sp.**

Species (isolate)	Position in alignment (ITS 1 | ITS 2)																													polym. sites (accessions)
	62	75-6	107	111	131	139-140	149	155	163-4	177	233	236	423	458	492-3	505	552	559	561	562	584	597	601	646-9	660	662	676	680-1	729	
S. pectinata genotype 1a (**133**, **981**)	C	AC	G	C	G	GC	C	T	TG	T	G	C	T	C	AA	C	C	A	C	G	A	T	T	AACC	T	A	C	GC	A	0 (2)
S. pectinata genotype 1a (**1010**, **1935**)	C	AC	G	C	G	GC	C	T	TG	T	G	C	T	C	AA	C	C	A	C	G	A	T	T	AACC	T	A	C	GC	A	0 (2)
S. pectinata genotype 1a (**2071**, **2116**)	C	AC	G	C	G	GC	C	T	TG	T	G	C	T	C	AA	C	C	A	C	G	A	T	T	AACC	T	A	C	GC	A	0 (2)
S. pectinata genotype 1a (**2448**, **2465**)	C	AC	G	C	G	GC	C	T	TG	T	G	C	T	C	AA	C	C	A	C	G	A	T	T	AACC	T	A	C	GC	A	0 (2)
S. pectinata genotype 1a (**2541**, **2545**, **2561**)	C	AC	G	C	G	GC	C	T	TG	T	G	C	T	C	AA	C	C	A	C	G	A	T	T	AACC	T	A	C	GC	A	0 (3)
S. pectinata genotype 1a (**2705-2708**)	C	AC	G	C	G	GC	C	T	TG	T	G	C	T	C	AA	C	C	A	C	G	A	T	T	AACC	T	A	C	GC	A	0 (4)
S. pectinata genotype 1a (2026)	m	AC	G	C	G	GC	C	T	TG	T	k	C	T	C	AA	C	C	A	C	G	A	T	T	AACC	T	A	C	GC	A	2
S. pectinata genotype 1a (2724-2726)	C	AC	G	C	G	GC	C	T	TG	T	G	C	T	C	AA	C	c/-	A	C	G	A	T	T	AACC	T	A	C	GC	R	1 + indel (3)
S. pectinata genotype 1a (989)	C	AC	G	C	G	GC	C	T	TG	T	G	C	T	C	AA	y	C	A	C	G	A	T	T	AACC	T	A	C	GC	A	1
S. pectinata genotype 1a (2694)	C	AC	G	C	G	GC	C	T	TG	T	G	C	T	C	AA	C	N	N	N	N	N	N	N	NNNN	N	N	N	NN	N	(0)
S. pectinata genotype 1a (2795)	C	AC	G	C	G	GC	C	T	TG	T	G	C	T	C	AA	C	C	A	y	G	A	T	T	AACC	T	M	y	GC	A	3
S. pectinata genotype 1a (3201)	C	AC	G	C	G	GC	C	T	TG	T	G	C	T	C	AA	C	C	R	C	G	A	T	T	AACC	T	A	C	GC	A	1
S. pectinata genotype 1a (2695-2699)	C	AC	G	C	G	km	C	T	TG	T	G	C	T	C	AA	C	C	A	y	G	A	T	T	AACC	T	A	C	GC	A	3 (5)
S. pectinata gt 1a × gt 1b (2689)	C	AC	G	C	G	GC	C	T	TG	T	k	C	Y	C	AA	C	C	A	C	G	A	T	T	AACC	T	A	C	GC	A	2
S. pectinata gt 1a × gt 1b (1023, 2589)	C	AC	G	C	G	GC	C	T	TG	T	G	C	y	C	AA	C	C	A	C	G	A	T	T	AACC	T	A	C	GC	A	1 (2)
S. pectinata gt 1a × gt 1b (2538)	C	AC	G	C	G	GC	C	T	TG	T	G	C	y	C	AA	C	C	r	C	G	A	T	T	AACC	T	A	C	GC	A	2
S. pectinata gt 1a × gt 1b (2210-2212)	C	AC	G	C	G	GC	C	T	TG	T	G	C	y	C	AA	C	c/-	A	C	G	A	T	T	AACC	T	A	C	GC	R	2 + indel (3)
S. pectinata gt 1a × gt 1b (2213)	C	AC	G	C	G	GC	C	T	TG	T	G	C	y	C	AA	C	c/-	A	C	G	A	W	T	AACC	T	A	C	GC	R	3 + indel
S. pectinata gt 1a × gt 1b (2586)	C	AC	G	C	G	GC	C	T	TG	T	G	C	y	C	AA	C	c/-	A	C	G	A	T	T	AACC	T	A	C	GC	r	2 + indel
S. pectinata gt 1a × gt 1b (2587)	C	AC	G	C	G	GC	C	T	TG	T	G	C	y	C	AA	C	c/-	A	C	G	A	T	T	AACC	T	A	C	GC	r	2 + indel
S. pectinata genotype 1b (**2040**)	C	AC	G	C	G	GC	C	T	TG	T	G	C	C	C	AA	C	C	A	C	G	A	T	T	AACC	T	A	C	GC	A	0
S. pectinata gt 1b × gt 2b (**2228**) major	C	AC	G	C	G	GC	C	T	TG	T	G	C	C	C	AA	C	C	A	C	G	A	T	T	AACC	T	A	N	NN	N	0 (allele)
S. pectinata gt 1a × gt 2a (2090)[1]	C	AC	s	s	G	KM	C	w	TG	T	G	C	y	C	AA	C	C	A	C	G	A	T	T	AACC	T	A	C	GC	A	6
S. pectinata gt 1a × gt 2a (2796)	C	AC	s	s	G	GC	C	T	TG	T	G	C	y	C	AA	C	C	A	C	G	r	T	T	wmCs	T	A	C	GC	A	7
S. pectinata gt 1b × gt 2a (2690)	C	AC	S	S	G	GC	C	T	G	T	G	C	C	S	AA	C	C	A	C	G	R	T	T	wmCs	T	A	C	GC	A	7
S. pectinata gt 1a × gt 2b (1711)	C	AC	S	S	R	GC	C	T	tg/--	T	G	C	Y	C	AA	C	C	A	C	G	R	T	T	wmCs	T	A	C	GC	A	8 + indel
S. pectinata gt 1a × gt 2e (**2228**) minor	C	TT	C	G	G	GC	C	T	--	T	G	C	C	C	AA	C	C	A	C	G	G	T	T	TCCG	T	A	N	NN	N	0 (allele)
S. pectinata gt 2c × gt 2d (**2283**) c4	C	TT	C	G	G	GC	C	T	TG	T	G	C	C	C	--	C	C	A	C	G	G	T	T	TCCG	T	A	C	GC	A	0 (clone)
S. pectinata gt 2c × gt 2d (**3210**) c4	C	TT	C	G	G	GC	C	T	TG	T	G	C	C	C	--	C	C	A	C	G	G	T	T	TCCG	T	A	C	GC	A	0 (clone)
S. pectinata gt 2b × gt 2c (**3225**) minor	C	TT	C	G	G	GC	C	T	TG	T	G	C	C	C	--	C	C	A	C	R	G	T	T	TCCG	T	A	C	GC	A	1 (allele)
S. pectinata genotype 2a (2797)	C	AC	C	G	G	GC	C	T	TG	T	G	C	C	s	AA	C	C	A	C	G	G	T	T	TCCG	T	A	C	GC	A	1
S. pectinata genotype 2a (**1837**)	C	AC	C	G	G	GC	C	T	TG	T	G	C	C	C	AA	C	C	A	C	G	G	T	T	TCCG	T	A	C	GC	A	0
S. pectinata genotype 2a (**1841**)	C	AC	C	G	G	GC	C	T	TG	T	G	C	C	C	AA	C	C	A	C	G	G	T	T	TCCG	T	A	C	GC	A	0
S. pectinata genotype 2a (**2051**)	C	AC	C	G	G	GC	C	T	TG	T	G	C	C	C	AA	C	C	A	C	G	G	T	T	TCCG	T	A	C	GC	A	0
S. pectinata genotype 2a (**2484-2488**)	C	AC	C	G	G	GC	C	T	TG	T	G	C	C	C	AA	C	C	A	C	G	G	T	T	TCCG	T	A	C	GC	A	0 (5)
S. pectinata gt 2a × gt 2b (1869)	C	AC	C	G	r	GC	C	T	tg/--	T	G	C	C	C	AA	C	C	A	C	G	G	T	T	TCCG	T	A	C	GC	A	1 + indel
S. pectinata genotype 2b (2644)	C	AC	C	G	r	GC	C	T	--	T	G	C	C	C	AA	C	C	A	C	r	G	T	T	TCCG	T	A	C	GC	A	2
S. pectinata genotype 2b (2920)	C	AC	C	G	r	GC	C	T	--	T	G	C	C	C	AA	C	C	A	C	G	G	T	T	TCCG	T	A	C	GC	A	1
S. pectinata genotype 2b (**1650**)	C	AC	C	G	G	GC	C	T	--	T	G	C	C	C	AA	C	C	A	C	G	G	T	T	TCCG	T	A	C	GC	A	0
S. pectinata genotype 2b (**1652**)	C	AC	C	G	G	GC	C	T	--	T	G	C	C	C	AA	C	C	A	C	G	G	T	T	TCCG	T	A	C	GC	A	0
S. pectinata gt 2c × gt 2d (**3210**) c9	C	AC	C	G	G	GC	C	T	--	T	G	C	C	C	AA	C	C	A	C	G	G	T	T	TCCG	T	A	C	TT	A	0 (clone)
S. pectinata gt 2c × gt 2d (**2283**) c2	C	AC	C	G	G	GC	C	T	--	T	G	C	C	C	AA	C	C	A	C	G	G	T	T	TCCG	T	A	C	TT	A	0 (clone)
S. pectinata gt 2b × gt 2c (**3225**) major	C	AC	C	G	G	GC	C	T	--	T	G	C	C	C	AA	C	C	A	C	R	G	T	T	TCCG	T	A	C	GC	A	1 (allele)
S. striata (**2185**)	C	AC	C	G	G	GC	y	T	TG	t/-	G	C	C	C	--	C	C	A	C	G	G	T	k	TCCG	y	A	C	GC	A	3 + indel
S. striata (**3029**)	C	AC	C	G	G	GC	y	T	TG	t/-	G	C	C	C	--	C	C	A	C	G	G	T	k	TCCG	y	A	C	GC	A	3 + indel
S. striata (**1034**)	C	AC	C	G	G	GC	Y	T	TG	–	G	s	C	C	--	C	C	A	C	G	G	T	G	TCCG	T	A	C	GC	A	2
S. striata × *S.* sp. (855) c3	C	AC	C	G	G	GC	C	T	TG	–	G	C	C	C	--	C	C	A	C	G	G	T	G	TCCG	T	A	C	GC	A	0 (clone)

[1] In the second half of this sequence, the additive character states of genotype 2 are missing (confirmed by several independent amplifications and sequencing reactions), probably as a result of gene conversion.

Legend: Variable positions are shown. Colors are for better distinction of the variation. Samples in bold cover the variation and were used for tree construction.
c: cloned sequences, major/minor: dominant ribotype/underrepresented ribotype in sequence electropherogram.

Supplementary Table 2 | Intra- and interspecific variation of closely related species, number of intra-individual polymorphic sites (*ITS*)

B) *Stuckenia amblyphylla*, *S. pamirica*, *S. filiformis* (genotypes 1 and 2 and intraspecific hybrids)**, *S. vaginata*** (genotypes 1, 2a and 2b)**, and a hybrid involving *S. filiformis* genotype 1** (second parent probably *S. vaginata*)

**Species (isolate)**	**Position in alignment (ITS 1 | ITS 2)**														**polym. sites (accessions)**
	**52**	**116**	**146-147**	**226**	**488**	**568**	**612**	**615**	**620**	**648**	**649**	**658**	**664**	**713-716**	
S. amblyphylla (**2602**)	G	A	–	T	A	C	T	C	G	T	C	T	A	–	0
S. amblyphylla (**2603**)	G	A	–	T	A	C	T	C	G	T	C	T	A	–	0
S. pamirica (**1753**)	A	A	AC	T	C	T	T	C	G	T	C	W	C	TATG/–	1 + indel
S. filiformis genotype 1 (**1987**, **1989**, **1992**, **2006**, **2288**, **2290**, **2291**, **2296**, **2297**, **2298**, **2322**, **3216**, **3217**, **3218**, **3229**, **3248**, **3252**)	A	A	–	T	C	T	T	C	G	T	C	T	C	–	0 (17)
S. filiformis genotype 1 × genotype 2 (1060)	R	A	–	T	C	T	T	C	G	T	C	T	C	–	1
S. filiformis genotype 1 × genotype 2 (1703)	R	A	–	T	C	T	T	C	G	T	C	T	C	–	1
S. filiformis genotype 1 × genotype 2 (1985)	R	A	–	T	C	T	T	C	G	T	C	T	C	–	1
S. filiformis genotype 1 × *S. vaginata* (2446)*	R	A	–	Y	C	T	T	S	G	T	C	T	C	–	3*
S. filiformis genotype 1 × *S. vaginata* (2452)*	r	A	–	Y	C	T	T	S	G	T	C	T	C	–	3*
[second parent of 2446 and 2452, predicted sequence]	G	A	–	C	C	T	T	G	G	T	C	T	C	–	-
S. filiformis genotype 2 (**1187**, **1941**, **2108**, **2134**, **2440**, **2453**, **2462**, **2463**, **2464**, **2543**, **2793**, **2794**, **3192**)	G	A	–	T	C	T	T	C	G	T	C	T	C	–	0 (13)
S. filiformis genotype 2 (2095)	G	A	–	T	C	T	T	C	G	Y	C	T	C	–	1
S. filiformis genotype 2 (2461)	G	A	–	T	C	T	T	C	G	T	S	T	C	–	1
S. vaginata genotype 1 (**1919**)	G	A	–	T	C	T	T	C	T	T	C	T	C	–	0
S. vaginata genotype 1 (**2052**)	G	A	–	T	C	T	T	C	T	T	C	T	C	–	0
S. vaginata genotype 1 (**2097**)	G	A	–	T	C	T	T	C	T	T	C	T	C	–	0
S. vaginata genotype 1 (**2132**)	G	A	–	T	C	T	T	C	T	T	C	T	C	–	0
S. vaginata genotype 2a (**1063**)	G	G	–	T	C	T	T	C	G	T	C	T	C	–	0
S. vaginata genotype 2a (**1976**)	G	G	–	T	C	T	T	C	G	T	C	T	C	–	0
S. vaginata genotype 2a (**1999**)	G	G	–	T	C	T	T	C	G	T	C	T	C	–	0
S. vaginata genotype 2b (**2016**)	G	G	–	T	C	T	A	C	G	T	C	T	C	–	0

* hybrids with an unknown genotype of another species, according to cpDNA similar to *S. vaginata*

Groenlandia densa: three identical sequences, no polymorphisms, no close relatives

Legend: Variable positions are shown. Colors are for better distinction of the variation. Samples in bold cover the variation and were used for tree construction.
c: cloned sequences, major/minor: dominant ribotype/underrepresented ribotype in sequence electropherogram.
