# Supplementary material for: Evolutionary history and patterns of geographical variation, fertility, and hybridization in Stuckenia (Potamogetonaceae)
Source: Front Plant Sci. 2022 Nov 3;13:1042517. doi: 10.3389/fpls.2022.1042517 (PMC9670304; doi:10.3389/fpls.2022.1042517)
Supplement: Supplementary file 7 [file Table_3.pdf]

Supplementary Table 3 | Intra- and interspecific variation of closely related species, number of intra-individual polymorphic sites (*5S-NTS*)

A) *Stuckenia pectinata* and *S. striata*

Species, isolate,	Position in alignment																																											
differential editing	10	11	13	14	17	18	19	23	29	30	31	32	35	37	40	43	45	47	54	55	60	61	63	70	73	74	80	81	82	83	86	87	88	91	92	93	95	96	97	98	105	106	107	110
S. pectinata 2026 poly	T	C	T	C	A	C	A	M	A	G	G	C	T	K	G	A	T	C	v	C	C	G	C	C	C	G	A	A	G	T	-	C	T	T	G	C	C	G	G	C	C	C	G	T
S. pectinata **2026** major	T	C	T	C	A	C	A	M	A	G	G	C	T	K	G	A	T	C	C	C	C	G	C	C	C	G	A	A	G	T	-	C	T	T	G	C	C	G	G	C	C	C	G	T
S. pectinata 2726 poly	T	C	T	v	A	s	A	m	A	G	G	C	T	G	G	A	T	C	C	C	C	G	C	C	C	G	A	A	G	T	-	C	T	T	G	C	C	G	r	C	C	C	G	T
S. pectinata **2726** major	T	C	T	C	A	C	A	C	A	G	G	C	T	G	G	A	T	C	C	C	C	G	C	C	C	G	A	A	G	T	-	C	T	T	G	C	C	G	G	C	C	C	G	T
S. pectinata 2705 poly	T	C	T	C	A	T	G	C	A	G	G	C	T	G	G	T	T	C	C	C	C	G	C	C	C	G	G	A	G	T	-	G	T	T	G	A	C	G	G	C	C	C	G	T
S. pectinata **2705** major	T	C	T	C	A	T	G	C	A	G	G	C	T	G	G	T	T	C	C	C	C	G	C	C	C	G	G	A	G	T	-	G	T	T	G	A	C	G	G	C	C	C	G	T
S. pectinata 2707 poly	T	C	T	C	A	T	G	C	A	G	G	C	T	G	G	T	T	C	C	C	C	G	C	C	C	G	G	A	G	T	-	G	T	T	G	A	C	G	G	C	C	C	G	T
S. pectinata **2707** major	T	C	T	C	A	T	G	C	A	G	G	C	T	G	G	T	T	C	C	C	C	G	C	C	C	G	G	A	G	T	-	G	T	T	G	A	C	G	G	C	C	C	G	T
S. pectinata 2538 poly	T	C	T	C	A	T	G	C	A	G	G	C	T	G	G	T	T	C	C	C	C	G	C	C	C	G	G	A	G	T	-	G	T	T	G	A	C	G	G	C	C	C	G	T
S. pectinata **2538** major	T	C	T	C	A	T	G	C	A	G	G	C	T	G	G	T	T	C	C	C	C	G	C	C	C	G	G	A	G	T	-	G	T	T	G	A	C	G	G	C	C	C	G	T
S. pectinata 133 poly	T	C	T	C	A	T	G	C	A	G	G	C	T	G	G	T	T	C	C	C	C	G	C	y	C	G	G	A	G	T	-	r	T	T	G	A	C	r	G	y	m	C	G	T
S. pectinata **133** major	T	C	T	C	A	T	G	C	A	G	G	C	T	G	G	T	T	C	C	C	C	G	C	C	C	G	G	A	G	T	-	A	T	T	G	A	C	G	G	C	C	C	G	T
S. pectinata 2448 poly	N	N	N	N	N	N	G	C	A	G	G	C	T	G	G	T	v	C	C	C	C	r	C	C	C	G	G	A	G	T	-	A	T	T	G	A	m	r	G	C	C	m	G	k
S. pectinata **2448** major	N	N	N	N	N	N	G	C	A	G	G	C	T	G	G	T	T	C	C	C	C	G	C	C	C	G	G	A	G	T	-	A	T	T	G	A	C	G	G	C	C	C	G	T
S. pectinata 2040 poly	T	C	T	C	A	T	G	C	A	G	G	C	T	G	G	C	T	C	C	C	C	G	C	C	C	G	G	A	G	A	-	A	T	T	G	A	C	G	G	C	C	C	G	T
S. pectinata **2040** major	T	C	T	C	A	T	G	C	A	G	G	C	T	G	G	C	T	C	C	C	C	G	C	C	C	G	G	A	G	A	-	A	T	T	G	A	C	G	G	C	C	C	G	T
S. pectinata 2561 poly	T	C	T	C	A	T	G	C	A	G	G	C	T	G	G	T	v	C	C	C	C	R	C	C	C	G	G	A	G	T	-	A	T	T	G	A	C	r	G	C	C	C	G	T
S. pectinata **2561** major	T	C	T	C	A	T	G	C	A	G	G	C	T	G	G	T	T	C	C	C	C	R	C	C	C	G	G	A	G	T	-	A	T	T	G	A	C	G	G	C	C	C	G	T
S. pectinata 2071 poly	T	C	T	C	A	T	G	C	A	G	G	C	T	G	G	T	T	C	y	C	C	r	C	C	C	G	G	A	G	T	-	A	T	T	G	A	C	G	G	C	C	C	G	T
S. pectinata **2071** major	T	C	T	C	A	T	G	C	A	G	G	C	T	G	G	T	T	C	C	C	C	G	C	C	C	G	G	A	G	T	-	A	T	T	G	A	C	G	G	C	C	C	G	T
S. pectinata 1935 poly	T	C	T	C	A	T	G	C	A	G	G	C	T	G	G	T	v	C	y	C	C	r	y	C	y	G	G	A	G	T	-	A	T	T	G	A	m	R	G	C	C	C	G	K
S. pectinata **1935** major	T	C	T	C	A	T	G	C	A	G	G	C	T	G	G	T	T	C	C	C	C	G	C	C	C	G	G	A	G	T	-	A	T	T	G	A	C	G	G	C	C	C	G	T
S. pectinata 2116 poly	T	C	T	C	r	C	A	M	A	G	G	y	T	G	G	A	T	C	C	C	C	r	C	C	C	G	R	R	G	T	-	m	Y	T	G	m	C	G	G	y	y	C	r	T
S. pectinata **2116** major	T	C	T	C	A	C	A	M	A	G	G	C	T	G	G	A	T	C	C	C	C	G	C	C	C	G	R	R	G	T	-	C	T	T	G	C	C	G	G	C	C	C	G	T
S. pectinata 2210 poly	T	C	T	C	r	C	A	m	A	G	G	Y	T	G	r	A	T	C	C	C	C	r	C	C	C	G	R	r	G	T	-	m	T	T	r	C	C	G	G	Y	C	C	G	T
S. pectinata **2210** major	T	C	T	C	A	C	A	A	A	G	G	C	T	G	G	A	T	C	C	C	C	G	C	C	C	G	A	A	G	T	-	C	T	T	G	C	C	G	G	C	C	C	G	T
S. pectinata 981 poly	T	C	T	C	G	C	A	C	A	G	G	C	T	G	G	A	T	C	C	C	C	G	C	C	C	G	G	G	G	T	-	C	v	T	G	m	C	G	r	C	C	C	G	T
S. pectinata **981** major	T	C	T	C	G	C	A	C	A	G	G	C	T	G	G	A	T	C	C	C	C	G	C	C	C	G	G	G	G	T	-	C	T	T	G	C	C	G	G	C	C	C	G	T
S. pectinata 2689 poly	T	C	T	C	r	C	A	m	A	G	G	C	T	G	G	A	T	C	C	C	v	G	C	C	C	G	r	r	G	T	-	m	T	T	G	v	C	G	r	v	C	v	G	v
S. pectinata **2689** major	T	C	T	C	G	C	A	C	A	G	G	C	T	G	G	A	T	C	C	C	C	G	C	C	C	G	G	G	G	T	-	C	T	T	G	C	C	G	G	C	C	C	G	T
S. pectinata 2690 poly	T	C	T	C	r	y	r	C	A	s	G	C	T	G	G	w	T	C	C	C	C	G	C	C	C	G	G	r	G	T	-	m	y	T	G	A	C	G	r	C	y	C	G	T
S. pectinata **2690** major	T	C	T	C	G	C	A	C	A	G	G	C	T	G	G	A	T	C	C	C	C	G	C	C	C	G	G	G	G	T	-	C	C	T	G	A	C	G	G	C	C	C	G	T
S. pectinata 3201 poly	T	C	T	C	R	Y	R	C	A	G	G	C	T	G	G	W	T	C	C	C	C	G	C	C	C	G	G	R	G	T	-	S	Y	T	G	A	C	G	G	C	y	C	r	T
S. pectinata **3201** major	T	C	T	C	G	C	A	C	A	G	G	C	T	G	G	W	T	C	C	C	C	G	C	C	C	G	G	G	G	T	-	S	Y	T	G	A	C	G	G	C	C	C	G	T
S. pectinata 2465 poly	T	C	T	C	A	Y	R	m	A	G	G	C	T	G	G	w	T	C	C	C	C	r	C	C	C	G	r	r	G	T	-	m	T	T	G	m	C	r	G	C	C	C	G	T
S. pectinata **2465** major	T	C	T	C	A	Y	R	C	A	G	G	C	T	G	G	T	T	C	C	C	C	G	C	C	C	G	G	A	G	T	-	A	T	T	G	A	C	G	G	C	C	C	G	T
S. pectinata 2797 poly	T	C	Y	C	A	T	R	C	A	G	s	C	y	G	r	T	T	C	C	C	v	G	C	C	C	r	G	R	G	T	-	A	T	T	G	m	C	G	r	C	C	C	G	T
S. pectinata 2797 major	T	C	Y	C	A	T	R	C	A	G	G	C	T	G	G	T	T	C	C	C	C	G	C	C	C	G	G	G	G	T	-	A	T	T	G	C	C	G	G	C	C	C	G	T
S. pectinata 3225 poly	T	C	m	C	A	T	A	C	A	G	G	C	v	G	G	T	T	C	C	C	C	G	C	C	C	G	G	G	G	T	t/-	A	T	T	G	C	C	G	r	C	C	C	G	T
S. pectinata **3225** major	T	C	C	C	A	T	A	C	A	G	G	C	C	G	G	T	T	C	C	C	C	G	C	C	C	G	G	G	G	T	-	A	T	T	G	C	C	G	G	C	C	C	G	T
S. pectinata 3210 poly	T	C	m	C	A	T	A	C	A	G	G	C	v	G	G	T	T	C	C	C	C	G	C	C	C	G	G	G	G	T	t/-	A	T	T	G	C	C	G	r	C	C	C	G	T
S. pectinata **3210** major	T	C	C	C	A	T	A	C	A	G	G	C	C	G	G	T	T	C	C	C	C	G	C	C	C	G	G	G	G	T	-	A	T	T	G	C	C	G	G	C	C	C	G	T
S. pectinata 1841 poly	T	C	m	C	A	T	A	C	A	G	s	C	Y	G	G	T	T	C	C	v	v	G	C	C	C	r	G	G	G	T	-	A	T	T	G	C	C	G	R	C	C	C	G	T
S. pectinata **1841** major	T	C	C	C	A	T	A	C	A	G	G	C	Y	G	G	T	T	C	C	C	C	G	C	C	C	G	G	G	G	T	-	A	T	T	G	C	C	G	G	C	C	C	G	T
S. pectinata 1869 poly	T	C	C	C	A	T	A	C	A	G	G	C	C	G	G	T	T	C	C	C	C	G	C	C	C	G	G	k	G	T	t/-	A	T	T	G	C	C	G	r	C	C	C	G	T
S. pectinata **1869** major	T	C	C	C	A	T	A	C	A	G	G	C	C	G	G	T	T	C	C	C	C	G	C	C	C	G	G	G	G	T	-	A	T	T	G	C	C	G	G	C	C	C	G	T
S. pectinata 2051 poly	T	C	C	C	A	T	A	C	A	G	G	C	Y	K	G	T	T	C	C	C	C	G	C	C	C	G	G	G	G	T	-	A	T	T	G	C	C	G	r	C	C	C	G	T
S. pectinata **2051** major	T	C	C	C	A	T	A	C	A	G	G	C	T	G	G	T	T	C	C	C	C	G	C	C	C	G	G	G	G	T	-	A	T	T	G	C	C	G	G	C	C	C	G	T
S. pectinata 2283 poly	T	c/-	C	C	A	T	A	C	A	G	G	C	Y	G	G	T	T	C	C	v	v	G	C	C	C	r	G	G	G	T	t/-	A	T	T	G	C	C	G	r	C	C	C	G	T
S. pectinata **2283** major	T	C	C	C	A	T	A	C	A	G	G	C	C	G	G	T	T	C	C	C	C	G	C	C	C	G	G	G	G	T	-	A	T	T	G	C	C	G	G	C	C	C	G	T
S. pectinata 2484 poly	T	c/-	C	C	A	T	A	C	A	G	s	C	v	G	G	T	T	C	C	C	C	r	C	C	v	r	G	G	G	T	-	A	T	T	G	C	C	G	R	C	C	C	G	T
S. pectinata **2484** major	T	C	C	C	A	T	A	C	A	G	C	C	T	G	G	T	T	C	C	C	C	G	C	C	C	G	G	G	G	T	-	A	T	T	G	C	C	G	G	C	C	C	G	T
S. pectinata 1652 poly	T	C	C	C	A	T	A	C	A	G	G	C	v	G	G	T	T	s	C	v	v	G	C	C	C	G	G	G	G	T	a/-	A	T	k	s	C	C	G	r	C	C	C	G	T
S. pectinata **1652** major	T	C	C	C	A	T	A	C	A	G	G	C	C	G	G	T	T	C	C	C	C	G	C	C	C	G	G	G	G	T	-	A	T	T	G	C	C	G	G	C	C	C	G	T
S. pectinata 2920 poly	G	C	T	C	A	T	A	C	T	G	G	C	T	k	G	T	T	C	C	v	C	r	C	C	C	G	G	G	r	T	-	A	T	T	G	C	C	G	G	C	C	C	G	T
S. pectinata **2920** major	G	C	T	C	A	T	A	C	T	G	G	C	T	G	G	T	T	C	C	C	C	G	C	C	C	G	G	G	G	T	-	A	T	T	G	C	C	G	G	C	C	C	G	T
S. striata 1034 poly	T	C	w	C	A	T	A	C	A	A	G	C	T	G	G	T	T	C	C	C	C	G	C	C	C	r	G	G	G	T	-	A	T	T	G	C	C	G	r	C	C	C	A	T
S. striata **1034** major	T	C	T	C	A	T	A	C	A	A	G	C	T	G	G	T	T	C	C	C	C	G	C	C	C	G	G	G	G	T	-	A	T	T	G	C	C	G	G	C	C	C	A	T

Species, isolate,	Position in alignment																																									
differential editing	112	113	115	117	119	124	125	126	127	128	129	130	131	136	137	139	140	141	142	143	147	151	152	153	155	157	158	160	162	168	169	170	171	175	176-184	185	187	188	189	190	191	192	196
S. pectinata 2026 poly	T	G	T	C	G	A	C	C	A	r	G	C	R	T	T	C	G	C	G	A	A	T	C	A	G	G	C	w	K	T	T	s	r	G	GGCGGTTCG	T	C	C	T	T	C	T	A
S. pectinata **2026** major	T	G	T	C	G	A	C	C	A	A	G	C	A	T	T	C	G	C	G	A	A	T	C	A	G	G	C	A	G	T	T	G	A	G	GGCGGTTCG	T	C	C	T	T	C	T	A
S. pectinata 2726 poly	T	G	T	C	G	A	C	C	A	r	G	C	R	T	T	C	G	C	G	A	A	T	C	A	r	G	C	A	G	T	T	G	A	G	GGCGGTTCG	T	C	v	T	T	C	T	A
S. pectinata **2726** major	T	G	T	C	G	A	C	C	A	A	G	C	G	T	T	C	G	C	G	A	A	T	C	A	G	G	C	A	G	T	T	G	A	G	GGCGGTTCG	T	C	C	T	T	C	T	A
S. pectinata 2705 poly	C	G	T	C	s	C	s	C	A	G	G	C	A	W	T	C	G	C	G	r	T	T	S	A	A	G	s	A	G	T	T	G	A	G	ggcggttcg/----	T	C	C	T	T	C	T	A
S. pectinata **2705** major	C	G	T	C	G	C	G	C	A	G	G	C	A	A	T	C	G	C	G	A	T	T	G	A	A	G	C	A	G	T	T	G	A	G	GGCGGTTCG	T	C	C	T	T	C	T	A
S. pectinata 2707 poly	C	G	T	C	s	C	S	C	A	G	G	C	A	W	T	C	G	C	G	R	T	T	S	A	A	G	S	A	G	T	T	G	A	G	ggcggttcg/----	T	C	C	T	T	C	T	A
S. pectinata **2707** major	C	G	T	C	G	C	G	C	A	G	G	C	A	W	T	C	G	C	G	A	T	T	S	A	A	G	C	A	G	T	T	G	A	G	GGCGGTTCG	T	C	C	T	T	C	T	A
S. pectinata 2538 poly	C	r	T	C	s	C	s	C	A	G	G	C	A	w	T	C	G	C	G	r	T	T	s	A	A	G	s	A	G	T	T	G	A	G	ggcggttcg/----	T	C	C	T	T	C	T	A
S. pectinata **2538** major	C	G	T	C	G	C	G	C	A	G	G	C	A	A	T	C	G	C	G	A	T	T	G	A	A	G	C	A	G	T	T	G	A	G	GGCGGTTCG	T	C	C	T	T	C	T	A
S. pectinata 133 poly	Y	s	T	C	G	C	C	C	A	G	G	C	A	W	T	C	G	C	G	A	T	T	C	A	A	G	C	A	G	T	T	G	A	G	GGCGGTTCG	T	C	C	Y	T	C	T	A
S. pectinata **133** major	Y	G	T	C	G	C	C	C	A	G	G	C	A	W	T	C	G	C	G	A	T	T	C	A	A	G	C	A	G	T	T	G	A	G	GGCGGTTCG	T	C	C	Y	T	C	T	A
S. pectinata 2448 poly	T	G	T	C	G	C	C	C	A	s	G	C	A	T	T	C	G	C	G	A	T	T	C	A	A	G	C	A	G	T	T	G	A	G	GGCGGTTCG	T	C	C	v	T	C	T	A
S. pectinata **2448** major	T	G	T	C	G	C	C	C	A	G	G	C	A	T	T	C	G	C	G	A	T	T	C	A	A	G	C	A	G	T	T	G	A	G	GGCGGTTCG	T	C	C	C	T	C	T	A
S. pectinata 2040 poly	T	G	T	C	G	C	C	C	A	G	G	v	A	T	T	C	G	m	G	A	T	C	C	A	A	G	C	A	G	T	T	G	A	G	GGCGGTTCG	T	C	s	T	T	C	T	A
S. pectinata **2040** major	T	G	T	C	G	C	C	C	A	G	G	C	A	T	T	C	G	C	G	A	T	C	C	A	A	G	C	A	G	T	T	G	A	G	GGCGGTTCG	T	C	C	T	T	C	T	A
S. pectinata 2561 poly	T	G	T	C	G	C	s	C	A	G	G	C	A	T	T	C	G	C	G	A	T	T	C	A	A	G	C	A	G	T	T	G	A	G	GGCGGTTCG	v	C	C	v	T	C	T	A
S. pectinata **2561** major	T	G	T	C	G	C	C	C	A	G	G	C	A	T	T	C	G	C	G	A	T	T	C	A	A	G	C	A	G	T	T	G	A	G	GGCGGTTCG	T	C	C	C	T	C	T	A
S. pectinata 2071 poly	T	G	T	C	G	y	C	C	A	s	G	C	A	T	T	C	G	C	G	A	T	T	C	A	A	G	C	A	G	T	T	G	A	G	GGCGGTTCG	T	C	C	y	T	C	T	A
S. pectinata **2071** major	T	G	T	C	G	C	C	C	A	G	G	C	A	T	T	C	G	C	G	A	T	T	C	A	A	G	C	A	G	T	T	G	A	G	GGCGGTTCG	T	C	C	C	T	C	T	A
S. pectinata 1935 poly	T	G	T	C	G	C	C	C	A	s	G	C	A	T	T	C	G	C	G	A	T	T	C	A	A	G	C	A	G	T	T	G	A	G	GGCGGTTCG	T	C	C	y	T	C	T	A
S. pectinata **1935** major	T	G	T	C	G	C	C	C	A	G	G	C	A	T	T	C	G	C	G	A	T	T	C	A	A	G	C	A	G	T	T	G	A	G	GGCGGTTCG	T	C	C	C	T	C	T	A
S. pectinata 2116 poly	w	G	T	C	G	A	C	C	A	G	G	C	A	T	T	C	r	C	G	A	A	T	C	A	G	G	C	w	G	T	T	G	A	G	GGCGGTTCG	T	C	C	T	T	C	T	A
S. pectinata **2116** major	T	G	T	C	G	A	C	C	A	G	G	C	A	T	T	C	G	C	G	A	A	T	C	A	G	G	C	A	G	T	T	G	A	G	GGCGGTTCG	T	C	C	T	T	C	T	A
S. pectinata 2210 poly	T	G	T	C	G	A	C	C	A	G	G	m	A	T	T	C	G	C	r	A	A	T	C	A	G	r	C	W	G	v	T	G	A	G	GGCGGTTCG	v	C	C	T	T	C	T	A
S. pectinata **2210** major	T	G	T	C	G	A	C	C	A	G	G	C	A	T	T	C	G	C	G	A	A	T	C	A	G	G	C	A	G	T	T	G	A	G	GGCGGTTCG	T	C	C	T	T	C	T	A
S. pectinata 981 poly	w	G	T	C	G	A	C	C	A	G	G	C	A	T	T	C	G	C	G	A	A	T	C	A	G	G	C	A	G	T	T	G	A	G	GGCGGTTCG	Y	C	C	T	T	C	T	A
S. pectinata **981** major	T	G	T	C	G	A	C	C	A	G	G	C	A	T	T	C	G	C	G	A	A	T	C	A	G	G	C	A	G	T	T	G	A	G	GGCGGTTCG	C	C	C	T	T	C	T	A
S. pectinata 2689 poly	T	G	T	C	G	A	C	C	A	r	G	C	A	k	w	C	r	C	G	A	A	T	C	A	G	r	C	w	G	T	T	G	w	G	GGyGGTTCG	v	C	v	T	T	C	T	A
S. pectinata **2689** major	T	G	T	C	G	A	C	C	A	G	G	C	A	T	T	C	G	C	G	A	A	T	C	A	G	G	C	A	G	T	T	G	A	G	GGCGGTTCG	C	C	C	T	T	C	T	A
S. pectinata 2690 poly	w	G	T	C	G	m	C	s	A	G	G	C	A	T	y	C	G	C	G	A	w	T	C	A	r	G	C	A	G	T	T	G	A	G	GGCGGTTCr	T	C	C	T	T	C	T	r
S. pectinata **2690** major	A	G	T	C	G	A	C	C	A	G	G	C	A	T	T	C	G	C	G	A	A	T	C	A	G	G	C	A	G	T	T	G	A	G	GGCGGTTCG	T	C	C	T	T	C	T	A
S. pectinata 3201 poly	M	G	T	C	G	M	s	C	A	G	G	C	A	w	T	C	G	C	G	A	w	T	s	A	R	G	C	A	G	T	T	G	A	G	GGCGGTTCG	T	C	C	T	T	C	T	A
S. pectinata **3201** major	M	G	T	C	G	A	C	C	A	G	G	C	A	T	T	C	G	C	G	A	A	T	C	A	G	G	C	A	G	T	T	G	A	G	GGCGGTTCG	T	C	C	T	T	C	T	A
S. pectinata 2465 poly	T	G	T	C	G	M	C	C	A	r	G	C	A	k	w	C	G	C	G	A	W	T	C	A	R	G	C	w	G	T	T	G	A	r	GGCGGTTCG	T	C	C	y	T	C	T	A
S. pectinata **2465** major	T	G	T	C	G	C	C	C	A	G	G	C	A	T	T	C	G	C	G	A	W	T	C	A	R	G	C	A	G	T	T	G	A	G	GGCGGTTCG	T	C	C	T	T	C	T	A
S. pectinata 2797 poly	T	G	T	y	G	y	C	R	M	G	G	C	A	W	T	C	G	C	G	A	T	T	C	A	A	G	C	A	G	T	T	G	A	G	GGCGGTTCG	T	Y	C	T	Y	C	k	R
S. pectinata 2797 major	T	G	T	C	G	C	C	R	m	G	G	C	A	A	T	C	G	C	G	A	T	T	C	A	A	G	C	A	G	T	T	G	A	G	GGCGGTTCG	T	Y	C	T	C	C	T	A
S. pectinata 3225 poly	T	G	T	C	G	C	C	A	C	G	G	C	w	A	T	C	G	C	G	A	T	T	C	r	A	G	C	A	G	T	T	G	A	G	GGCGGTTCG	T	T	C	T	C	C	T	A
S. pectinata **3225** major	T	G	T	C	G	C	C	A	C	G	G	C	A	A	T	C	G	C	G	A	T	T	C	A	A	G	C	A	G	T	T	G	A	G	GGCGGTTCG	T	T	C	T	C	C	T	A
S. pectinata 3210 poly	T	G	T	C	G	C	C	A	C	G	G	C	A	A	T	C	G	C	G	A	T	T	C	R	A	G	C	A	G	T	T	G	A	G	GGCGGTTCG	T	T	C	T	C	C	T	A
S. pectinata **3210** major	T	G	T	C	G	C	C	A	C	G	G	C	A	A	T	C	G	C	G	A	T	T	C	A	A	G	C	A	G	T	T	G	A	G	GGCGGTTCG	T	T	C	T	C	C	T	A
S. pectinata 1841 poly	T	G	T	v	k	C	C	A	C	G	G	C	A	A	T	C	G	C	G	A	T	T	C	A	A	G	C	A	G	T	T	G	A	G	GGCGGTTCr	T	T	C	T	C	C	T	A
S. pectinata **1841** major	T	G	T	C	G	C	C	A	C	G	G	C	A	A	T	C	G	C	G	A	T	T	C	A	A	G	C	A	G	T	T	G	A	G	GGCGGTTCG	T	T	C	T	C	C	T	A
S. pectinata 1869 poly	T	G	T	C	G	C	C	A	C	G	G	C	A	A	T	C	G	C	G	A	T	T	C	A	A	G	C	A	G	T	T	G	A	G	GGCGGTwCG	T	T	C	T	C	C	T	A
S. pectinata **1869** major	T	G	T	C	G	C	C	A	C	G	G	C	A	A	T	C	G	C	G	A	T	T	C	A	A	G	C	A	G	T	T	G	A	G	GGCGGTTCG	T	T	C	T	C	C	T	A
S. pectinata 2051 poly	T	G	T	C	G	C	C	A	C	G	G	C	A	A	T	C	G	C	G	A	T	T	C	A	A	G	C	A	G	T	T	G	w	G	GGCGGTTCG	T	T	C	T	C	s	T	A
S. pectinata **2051** major	T	G	T	C	G	C	C	A	C	G	G	C	A	A	T	C	G	C	G	A	T	T	C	A	A	G	C	A	G	T	T	G	A	G	GGCGGTTCG	T	T	C	T	C	C	T	A
S. pectinata 2283 poly	T	r	T	y	G	C	C	A	C	G	G	C	w	A	T	C	G	C	G	A	T	T	C	R	A	G	C	A	G	T	T	G	A	G	GGCGGTTCG	T	T	C	T	C	C	T	A
S. pectinata **2283** major	T	G	T	C	G	C	C	A	C	G	G	C	A	A	T	C	G	C	G	A	T	T	C	R	A	G	C	A	G	T	T	G	A	G	GGCGGTTCG	T	T	C	T	C	C	T	A
S. pectinata 2484 poly	T	G	T	Y	G	C	C	A	C	G	k	C	A	A	T	C	G	C	G	A	T	T	C	A	A	G	C	A	G	w	T	G	A	G	GGCGGTTCr	T	T	C	T	C	C	T	A
S. pectinata **2484** major	T	G	T	Y	G	C	C	A	C	G	G	C	A	A	T	C	G	C	G	A	T	T	C	A	A	G	C	A	G	T	T	G	A	G	GGCGGTTCG	T	T	C	T	C	C	T	A
S. pectinata 1652 poly	T	G	T	C	G	C	C	A	C	G	G	C	w	A	T	C	G	C	G	A	T	T	C	A	A	G	C	A	G	T	T	G	A	G	GGCGGTTCG	T	T	C	T	C	C	T	A
S. pectinata **1652** major	T	G	T	C	G	C	C	A	C	G	G	C	A	A	T	C	G	C	G	A	T	T	C	A	A	G	C	A	G	T	T	G	A	G	GGCGGTTCG	T	T	C	T	C	C	T	A
S. pectinata 2920 poly	C	G	A	C	G	C	C	C	C	G	G	C	A	T	T	T	G	C	G	A	T	T	A	A	A	G	C	A	G	C	G	T	A	G	GGCGGTTCG	T	T	C	T	T	C	T	A
S. pectinata **2920** major	C	G	A	C	G	C	C	C	C	G	G	C	A	T	T	T	G	C	G	A	T	T	A	A	A	G	C	A	G	C	G	T	A	G	GGCGGTTCG	T	T	C	T	T	C	T	A
S. striata 1034 poly	T	G	A	C	G	C	C	C	C	G	G	C	A	T	T	T	G	C	G	A	T	T	A	A	A	G	C	A	G	C	G	T	A	G	GGCGGTyCG	T	T	C	T	T	C	T	A
S. striata **1034** major	T	G	A	C	G	C	C	C	C	G	G	C	A	T	T	T	G	C	G	A	T	T	A	A	A	G	C	A	G	C	G	T	A	G	GGCGGTTCG	T	T	C	T	T	C	T	A

Species, isolate,	Position in alignment																																								no. of poly-	no. of
differential editing	197	201	203	204	205	206	207	209	210	211	214	215	217	218	219	220	223	224	225	226	227	228	234	237	238	239	244	245	247	249	250	252	253	254	258	259	260	261	263	264	270	morphisms	indels
S. pectinata 2026 poly	G	C	A	G	C	T	A	s	C	T	A	-	G	A	G	G	A	C	C	C	G	A	C	A	A	C	C	T	C	T	G	A	G	A	C	A	T	G	A	T	G	10	0
S. pectinata **2026** major	G	C	A	G	C	T	A	G	C	T	A	-	G	A	G	G	A	C	C	C	G	A	C	A	A	C	C	T	C	T	G	A	G	A	C	A	T	G	A	T	G	2	0
S. pectinata 2726 poly	G	C	A	G	C	T	r	G	C	T	A	-	G	A	G	k	A	C	C	C	G	A	C	A	A	C	Y	T	C	T	G	A	G	A	C	A	T	G	A	T	G	11	0
S. pectinata **2726** major	G	C	A	G	C	T	A	G	C	T	A	-	G	A	G	G	A	C	C	C	G	A	C	A	A	C	Y	T	C	T	G	A	G	A	C	A	T	G	A	T	G	1	0
S. pectinata 2705 poly	G	C	A	A	C	C	A	G	C	T	A	-	k	A	s	G	A	C	C	C	G	A	M	A	A	C	C	T	C	T	G	A	R	A	C	A	G	G	A	G	G	10	1
S. pectinata **2705** major	G	C	A	A	C	C	A	G	C	T	A	-	G	A	C	G	A	C	C	C	G	A	C	A	A	C	C	T	C	T	G	A	R	A	C	A	G	G	A	G	G	1	0
S. pectinata 2707 poly	G	C	A	A	C	C	A	G	C	T	A	-	G	A	S	G	A	C	C	C	G	A	M	A	A	C	C	T	C	T	G	A	R	A	C	A	G	G	A	G	G	9	1
S. pectinata **2707** major	G	C	A	A	C	C	A	G	C	T	A	-	G	A	C	G	A	C	C	C	G	A	C	A	A	C	C	T	C	T	G	A	R	A	C	A	G	G	A	G	G	3	0
S. pectinata 2538 poly	G	C	A	A	C	C	A	G	C	T	A	-	k	A	s	G	A	C	C	C	G	A	m	A	A	C	C	T	C	T	G	A	G	A	C	A	G	G	A	G	G	10	1
S. pectinata **2538** major	G	C	A	A	C	C	A	G	C	T	A	-	G	A	C	G	A	C	C	C	G	A	C	A	A	C	C	T	C	T	G	A	G	A	C	A	G	G	A	G	G	0	0
S. pectinata 133 poly	G	C	A	A	C	C	A	G	C	T	A	-	G	A	C	G	A	C	C	C	G	A	C	A	A	C	C	T	C	T	G	A	G	A	C	A	G	G	A	G	G	9	0
S. pectinata **133** major	G	C	A	A	C	C	A	G	C	T	A	-	G	A	C	G	A	C	C	C	G	A	C	A	A	C	C	T	C	T	G	A	G	A	C	A	G	G	A	G	G	3	0
S. pectinata 2448 poly	G	C	A	A	C	C	A	G	C	T	A	-	G	A	C	G	A	C	C	C	G	A	C	A	A	C	C	T	C	T	G	A	G	A	C	A	r	G	A	G	G	9	0
S. pectinata **2448** major	G	C	A	A	C	C	A	G	C	T	A	-	G	A	C	G	A	C	C	C	G	A	C	A	A	C	C	T	C	T	G	A	G	A	C	A	G	G	A	G	G	0	0
S. pectinata 2040 poly	G	C	A	A	C	C	A	G	C	T	A	-	G	w	G	G	A	C	C	C	G	A	C	r	A	C	C	T	C	v	G	A	G	G	C	A	G	k	w	G	G	8	0
S. pectinata **2040** major	G	C	A	A	C	C	A	G	C	T	A	-	G	A	G	G	A	C	C	C	G	A	C	G	A	C	C	T	C	T	G	A	G	G	C	A	G	G	A	G	G	0	0
S. pectinata 2561 poly	G	m	A	A	C	C	A	G	C	T	A	-	G	A	v	G	A	C	C	v	k	A	C	A	A	C	C	T	C	T	G	A	G	A	C	A	r	G	A	G	k	12	0
S. pectinata **2561** major	G	C	A	A	C	C	A	G	C	T	A	-	G	A	C	G	A	C	C	C	G	A	C	A	A	C	C	T	C	T	G	A	G	A	C	A	G	G	A	G	G	1	0
S. pectinata 2071 poly	G	C	A	A	C	C	A	G	C	T	A	-	G	A	C	G	A	C	C	C	G	A	C	A	A	C	C	T	C	T	G	A	G	A	C	A	G	G	A	G	G	5	0
S. pectinata **2071** major	G	C	A	A	C	C	A	G	C	T	A	-	G	A	C	G	A	C	C	C	G	A	C	A	A	C	C	T	C	T	G	A	G	A	C	A	G	G	A	G	G	0	0
S. pectinata 1935 poly	G	C	A	A	C	C	A	G	m	T	A	-	G	A	C	G	r	C	C	C	G	A	C	A	A	C	C	T	C	T	G	A	G	A	C	A	R	G	A	G	G	13	0
S. pectinata **1935** major	G	C	A	A	C	C	A	G	C	T	A	-	G	A	C	G	A	C	C	C	G	A	C	A	A	C	C	T	C	T	G	A	G	A	C	A	G	G	A	G	G	0	0
S. pectinata 2116 poly	k	m	A	G	C	T	A	G	y	T	A	-	r	A	G	G	A	C	C	C	G	A	C	A	A	C	C	T	C	T	G	A	G	A	C	A	y	G	A	T	G	20	0
S. pectinata **2116** major	G	C	A	G	C	T	A	G	C	T	A	-	G	A	G	G	A	C	C	C	G	A	C	A	A	C	C	T	C	T	G	A	G	A	C	A	T	G	A	T	G	3	0
S. pectinata 2210 poly	k	M	A	G	s	T	A	G	v	T	A	-	r	A	G	G	A	C	C	C	G	A	C	A	A	C	C	T	C	T	G	A	G	A	C	A	v	G	A	T	G	22	0
S. pectinata **2210** major	G	C	A	G	C	T	A	G	C	T	A	-	G	A	G	G	A	C	C	C	G	A	C	A	A	C	C	T	C	T	G	A	G	A	C	A	T	G	A	T	G	0	0
S. pectinata 981 poly	G	C	A	G	C	T	A	G	C	T	A	-	G	A	G	G	A	C	C	C	G	A	C	A	A	C	C	T	S	T	G	A	G	A	C	A	T	G	A	T	G	6	0
S. pectinata **981** major	G	C	A	G	C	T	A	G	C	T	A	-	G	A	G	G	A	C	C	C	G	A	C	A	A	C	C	T	S	T	G	A	G	A	C	A	T	G	A	T	G	1	0
S. pectinata 2689 poly	G	m	A	G	C	T	A	s	C	T	A	-	G	A	G	G	A	C	C	C	G	A	C	A	A	C	C	T	b	T	G	w	G	A	C	A	T	G	A	T	G	25	0
S. pectinata **2689** major	G	C	A	G	C	T	A	G	C	T	A	-	G	A	G	G	A	C	C	C	G	A	C	A	A	C	C	T	S	T	G	A	G	A	C	A	T	G	A	T	G	1	0
S. pectinata 2690 poly	G	C	A	r	C	Y	A	G	C	T	A	-	G	A	G	G	A	C	C	C	G	A	C	A	A	C	C	T	C	T	G	A	G	A	C	A	k	G	A	k	G	22	0
S. pectinata **2690** major	G	C	A	G	C	T	A	G	C	T	A	-	G	A	G	G	A	C	C	C	G	A	C	A	A	C	C	T	C	T	G	A	G	A	C	A	T	G	A	T	G	0	0
S. pectinata 3201 poly	G	C	A	R	C	Y	A	G	C	T	A	-	G	A	s	G	A	C	C	C	G	A	C	A	A	C	C	T	C	T	G	A	G	A	C	A	T	G	A	T	G	19	0
S. pectinata **3201** major	G	C	A	R	C	Y	A	G	C	T	A	-	G	A	G	G	A	C	C	C	G	A	C	A	A	C	C	T	C	T	G	A	G	A	C	A	T	G	A	T	G	6	0
S. pectinata 2465 poly	G	C	A	r	C	Y	A	G	C	T	A	-	G	A	S	G	A	C	C	C	G	A	C	A	A	C	C	T	C	T	G	A	G	A	C	A	K	G	A	K	G	24	0
S. pectinata **2465** major	G	C	A	A	C	C	A	G	C	T	A	-	G	A	S	G	A	C	C	C	G	A	C	A	A	C	C	T	C	T	G	A	G	A	C	A	K	G	A	K	G	7	0
S. pectinata 2797 poly	G	C	A	A	C	Y	A	K	C	T	A	-	G	A	G	G	A	C	m	C	G	A	C	A	R	C	C	T	C	T	G	A	G	A	S	A	G	G	A	G	G	24	0
S. pectinata 2797 major	G	C	A	A	C	Y	A	K	C	T	A	-	G	A	G	G	A	C	C	C	G	A	C	A	R	C	C	T	C	T	G	A	G	A	S	A	G	G	A	G	G	9	0
S. pectinata 3225 poly	G	C	r	A	C	T	A	T	C	v	A	-	G	A	G	G	A	C	C	C	G	A	C	A	G	C	C	T	C	T	G	A	G	A	G	A	G	G	A	G	G	8	1
S. pectinata **3225** major	G	C	A	A	C	T	A	T	C	T	A	-	G	A	G	G	A	C	C	C	G	A	C	A	G	C	C	T	C	T	G	A	G	A	G	A	G	G	A	G	G	0	0
S. pectinata 3210 poly	G	C	r	A	C	T	A	T	C	Y	A	-	G	A	G	G	A	C	C	C	G	A	C	A	G	s	C	T	C	T	G	A	G	A	G	A	G	G	A	G	G	7	1
S. pectinata **3210** major	G	C	A	A	C	T	A	T	C	T	A	-	G	A	G	G	A	C	C	C	G	A	C	A	G	C	C	T	C	T	G	A	G	A	G	A	G	G	A	G	G	0	0
S. pectinata 1841 poly	r	C	A	A	C	T	A	T	C	T	A	-	r	A	G	G	A	C	M	C	G	A	C	A	G	C	C	T	C	T	G	A	G	A	G	A	G	G	A	G	G	13	0
S. pectinata **1841** major	G	C	A	A	C	T	A	T	C	T	A	-	G	A	G	G	A	C	C	C	G	A	C	A	G	C	C	T	C	T	G	A	G	A	G	A	G	G	A	G	G	1	0
S. pectinata 1869 poly	G	C	A	A	C	T	A	T	C	T	A	-	G	A	G	G	A	C	C	C	G	A	C	A	G	S	C	T	C	T	G	A	G	A	G	A	G	G	A	G	G	4	1
S. pectinata **1869** major	G	C	A	A	C	T	A	T	C	T	A	-	G	A	G	G	A	C	C	C	G	A	C	A	G	S	C	T	C	T	G	A	G	A	G	A	G	G	A	G	G	1	0
S. pectinata 2051 poly	G	C	A	A	C	T	r	T	C	T	A	a/-	G	A	G	G	A	C	m	C	G	A	C	A	G	C	C	T	C	T	G	A	G	A	G	A	G	G	A	G	G	7	1
S. pectinata **2051** major	G	C	A	A	C	T	A	T	C	T	A	-	G	A	G	G	A	C	C	C	G	A	C	A	G	C	C	T	C	T	G	A	G	A	G	A	G	G	A	G	G	0	0
S. pectinata 2283 poly	G	C	A	A	C	T	A	T	C	T	A	-	G	A	G	G	A	C	C	C	G	A	C	A	G	C	C	T	C	T	G	A	G	A	G	A	G	G	A	G	G	9	2
S. pectinata **2283** major	G	C	A	A	C	T	A	T	C	T	A	-	G	A	G	G	A	C	C	C	G	A	C	A	G	C	C	T	C	T	G	A	G	A	G	A	G	G	A	G	G	1	0
S. pectinata 2484 poly	G	C	A	A	C	T	A	T	C	T	r	-	G	A	G	G	A	C	m	C	G	A	C	A	G	C	C	T	C	T	G	A	G	A	G	A	G	G	A	G	G	12	1
S. pectinata **2484** major	G	C	A	A	C	T	A	T	C	T	A	-	G	A	G	G	A	C	C	C	G	A	C	A	G	C	C	T	C	T	G	A	G	A	G	A	G	G	A	G	G	1	0
S. pectinata 1652 poly	G	C	A	A	C	T	A	T	C	T	A	-	G	A	G	G	A	C	C	C	G	A	C	A	G	C	C	T	C	T	G	A	G	A	G	A	G	G	A	G	G	8	1
S. pectinata **1652** major	G	C	A	A	C	T	A	T	C	T	A	-	G	A	G	G	A	C	C	C	G	A	C	A	G	C	C	T	C	T	G	A	G	A	G	A	G	G	A	G	G	0	0
S. pectinata 2920 poly	G	C	A	A	S	A	A	G	M	T	A	-	G	A	G	G	A	G	C	C	G	A	C	A	A	C	C	w	C	T	C	A	G	A	C	A	G	G	A	G	G	7	0
S. pectinata **2920** major	G	C	A	A	S	A	A	G	M	T	A	-	G	A	G	G	A	G	C	C	G	A	C	A	A	C	C	W	C	T	C	A	G	A	C	A	G	G	A	G	G	3	0
S. striata 1034 poly	G	C	A	A	S	A	w	G	C	T	A	-	G	A	G	G	A	C	C	C	G	M	C	A	A	C	C	T	C	T	s	A	C	A	C	C	G	A	A	G	G	9	0
S. striata **1034** major	G	C	A	A	G	A	A	G	C	T	A	-	G	A	G	G	A	C	C	C	G	A	C	A	A	C	C	T	C	T	C	A	C	A	C	C	G	A	A	G	G	0	0

Legend: Intraspecific and intra-individual (polymorphisms) variation is summarized; variable positions are shown. Positions in the alignment start with the first base after the forward primer. Colors are for better distinction of the variation. Samples in bold cover the variation and were used for tree construction. poly: all polymorphisms included, major: dominant ribotype (excluding small additional peaks).
130 positions with variation (including variation in longer indel) out of 272 characters = 47.8%

B) *Stuckenia amblyphylla* and *S. filiformis*

Species, isolate,	Position in alignment																																						
differential editing, clone	14	17	19	37	41	42	54	55	60	76	80	82	84	85	86	89	99	101	106	111	113	114	115	116	122	126	134	137	138	139	143	145	151	152	157	161	162	169	170
S. amblyphylla 2602 poly	C	A	A	G	G	A	C	C	y	T	G	G	T	T	A	T	C	C	G	T	T	T	T	C	G	C	G	r	y	G	T	K	C	A	m	k	T	T	A
S. amblyphylla **2602** major	C	A	A	G	G	A	C	C	C	T	G	G	T	T	A	T	C	C	G	T	T	T	T	C	G	C	G	A	C	G	T	G	C	A	A	G	T	T	A
S. amblyphylla 2603 poly	C	A	A	G	G	A	C	C	y	T	G	G	T	T	A	T	C	C	G	T	T	T	T	C	G	C	G	r	y	G	T	K	C	A	m	k	T	T	A
S. amblyphylla **2603** major	C	A	A	G	G	A	C	C	C	T	G	G	T	T	A	T	C	C	G	T	T	T	T	C	G	C	G	A	C	G	T	G	C	A	A	G	T	T	A
S. filiformis 1985 poly	C	G	A	G	G	A	C	C	C	T	G	s	w	w	A	T	C	C	G	T	y	T	K	C	G	y	G	G	T	s	T	G	A	w	C	G	T	T	A
S. filiformis **1985** major	C	G	A	G	G	A	C	C	C	T	G	G	T	T	A	T	C	C	G	T	T	T	K	C	G	C	G	G	T	G	T	G	A	A	C	G	T	T	A
S. filiformis 1987 poly	C	G	A	G	G	A	C	C	C	w	G	s	w	w	A	T	C	C	G	T	T	T	K	C	G	C	G	G	T	G	T	G	A	A	C	G	T	T	A
S. filiformis **1987** major	C	G	A	G	G	A	C	C	C	T	G	G	T	T	A	T	C	C	G	T	T	T	K	C	G	C	G	G	T	G	T	G	A	A	C	G	T	T	A
S. filiformis 2290 poly	C	G	A	G	G	A	C	C	C	w	G	s	w	T	A	T	C	C	G	T	T	T	k	C	G	C	G	G	T	s	T	G	A	A	C	G	T	T	A
S. filiformis **2290** major	C	G	A	G	G	A	C	C	C	T	G	G	T	T	A	T	C	C	G	T	T	T	G	C	G	C	G	G	T	G	T	G	A	A	C	G	T	T	A
S. filiformis 2322 poly	C	G	A	G	G	A	C	y	C	w	G	s	w	w	A	T	C	C	G	T	T	T	K	C	G	C	G	G	T	G	T	G	A	A	C	G	T	T	A
S. filiformis **2322** major	C	G	A	G	G	A	C	C	C	T	G	G	T	T	A	T	C	C	G	T	T	T	K	C	G	C	G	G	T	G	T	G	A	A	C	G	T	T	A
S. filiformis 3192 poly	C	R	A	R	R	A	B	C	C	T	r	G	T	T	A	k	m	C	r	T	T	Y	T	C	G	C	G	R	T	G	T	G	m	A	C	G	T	y	A
S. filiformis **3192** major	C	R	A	G	G	A	C	C	C	T	G	G	T	T	A	T	C	C	G	T	T	T	T	C	G	C	G	R	T	G	T	G	C	A	C	G	T	T	A
S. filiformis 3192 clone 1	C	A	A	A	A	A	C	C	C	T	G	G	T	T	A	T	C	C	G	T	T	T	T	C	G	C	G	G	T	G	T	G	A	A	C	G	T	T	A
S. filiformis 3192 clone 2	C	A	A	G	A	A	C	C	C	T	G	G	T	T	A	T	C	C	G	T	T	T	T	C	G	C	G	A	T	G	T	G	C	A	C	G	T	C	A
S. filiformis 3192 clone 3	C	G	A	G	G	A	C	C	C	T	G	G	T	T	A	G	C	C	A	T	T	T	T	C	G	C	G	A	T	G	T	G	C	A	C	G	T	C	A
S. filiformis 3192 clone 4	C	G	A	G	G	A	C	C	C	T	G	G	T	T	A	T	C	C	G	T	T	C	T	C	G	C	G	G	T	G	T	G	A	A	C	G	T	T	A
S. filiformis 3192 clone 5	C	G	A	G	G	A	C	C	C	T	G	G	T	T	A	T	A	C	G	T	T	C	T	C	G	C	G	G	T	G	T	G	A	A	C	G	T	T	A
S. filiformis 1060 poly	N	N	N	R	R	W	C	C	C	T	G	G	T	T	A	T	m	m	G	T	T	Y	T	C	G	C	G	r	T	G	T	G	M	A	C	G	T	T	A
S. filiformis **1060** major	N	N	N	G	G	A	C	C	C	T	G	G	T	T	A	T	C	C	G	T	T	T	T	C	G	C	G	G	T	G	T	G	M	A	C	G	T	T	A
S. filiformis 1703 poly	m	R	A	R	R	A	C	C	C	T	G	G	T	T	A	T	C	C	G	T	T	T	T	C	G	C	G	R	T	G	y	G	M	A	C	G	T	y	A
S. filiformis **1703** major	C	R	A	G	R	A	C	C	C	T	G	G	T	T	A	T	C	C	G	T	T	T	T	C	G	C	G	R	T	G	T	G	C	A	C	G	T	T	A
S. filiformis 2095 poly	C	A	A	G	G	A	C	C	C	T	G	G	T	T	A	T	C	C	G	T	T	T	T	C	k	C	k	A	C	G	T	G	M	A	C	G	T	T	A
S. filiformis **2095** major	C	A	A	G	G	A	C	C	C	T	G	G	T	T	A	T	C	C	G	T	T	T	T	C	T	C	G	A	C	G	T	G	C	A	C	G	T	T	A
S. filiformis 2095 clone 1	C	A	A	G	G	A	C	C	C	T	G	G	T	T	A	T	C	C	G	T	T	T	T	C	G	C	T	G	T	G	T	G	A	A	C	G	A	T	A
S. filiformis 2095 clone 2	C	G	A	G	G	A	C	C	C	T	G	G	T	T	A	T	C	C	G	T	T	T	T	C	G	C	T	G	T	G	T	G	A	A	C	G	A	T	A
S. filiformis 2095 clone 3	C	A	A	G	G	A	C	C	C	T	G	G	T	T	A	T	C	C	G	T	T	T	T	C	G	C	T	G	T	G	T	G	A	A	C	G	A	T	A
S. filiformis 2095 clone 4	C	A	A	A	G	A	C	C	C	T	G	G	T	T	A	T	C	C	G	T	T	T	T	C	T	C	G	A	C	G	T	G	C	A	C	G	T	T	A
S. filiformis 2095 clone 5	C	A	A	G	G	A	C	C	C	T	G	G	T	T	A	T	C	C	G	T	T	T	T	C	T	C	G	A	C	G	T	G	C	A	C	G	T	T	A
S. filiformis 1941 poly	C	A	A	G	G	A	C	C	C	T	G	G	T	T	A	T	C	C	G	T	T	T	T	C	T	C	G	A	C	G	T	G	C	A	C	G	w	T	A
S. filiformis **1941** major	C	A	A	G	G	A	C	C	C	T	G	G	T	T	A	T	C	C	G	T	T	T	T	C	T	C	G	A	C	G	T	G	C	A	C	G	T	T	A
S. filiformis 2440 poly	C	A	A	G	G	A	C	C	y	T	G	G	T	T	r	T	C	C	G	T	T	T	T	C	G	C	G	r	y	G	T	k	m	A	m	G	T	T	A
S. filiformis **2440** major	C	A	A	G	G	A	C	C	C	T	G	G	T	T	A	T	C	C	G	T	T	T	T	C	G	C	G	A	C	G	T	G	C	A	A	G	T	T	A
S. filiformis 2462 poly	C	A	A	G	G	A	C	C	C	T	G	G	T	T	A	T	C	C	G	T	T	T	T	C	T	C	G	A	C	G	T	G	C	A	C	G	T	T	w
S. filiformis **2462** major	C	A	A	G	G	A	C	C	C	T	G	G	T	T	A	T	C	C	G	T	T	T	T	C	T	C	G	A	C	G	T	G	C	A	C	G	T	T	A
S. filiformis 2462 clone 2	C	A	A	G	G	A	C	C	T	T	G	G	T	T	A	T	C	C	G	T	T	T	T	C	G	C	G	A	C	G	T	G	C	A	C	G	T	T	A
S. filiformis 2462 clone 3	C	A	A	G	G	A	C	C	C	T	G	G	T	T	A	T	C	C	G	T	T	T	T	C	T	C	G	A	C	G	T	G	C	A	C	G	T	T	A
S. filiformis 2462 clone 5	C	A	A	G	G	A	C	C	C	T	G	G	T	T	A	T	C	C	G	T	T	T	T	C	T	C	G	A	C	G	T	G	C	A	C	G	T	T	A
S. filiformis 2543 poly	C	A	m	G	R	A	C	C	C	T	G	G	T	T	A	T	C	C	G	w	T	T	T	C	G	C	s	A	Y	G	T	G	C	m	M	G	T	T	A
S. filiformis **2543** major	C	A	A	G	R	A	C	C	C	T	G	G	T	T	A	T	C	C	G	T	T	T	T	C	G	C	G	A	Y	G	T	G	C	A	M	G	T	T	A
S. filiformis 1187 poly	C	A	m	G	R	A	C	C	C	T	G	G	T	T	A	T	C	C	G	T	T	T	T	C	G	C	s	A	Y	G	T	G	C	m	M	G	T	T	A
S. filiformis **1187** major	C	A	A	G	R	A	C	C	C	T	G	G	T	T	A	T	C	C	G	T	T	T	T	C	G	C	G	A	Y	G	T	G	C	A	M	G	T	T	A
S. filiformis 2794 poly	C	A	A	G	G	A	C	C	C	T	G	G	T	T	A	T	C	C	G	T	T	T	T	C	G	C	G	A	C	G	T	k	C	A	m	G	T	T	A
S. filiformis **2794** major	C	A	A	G	G	A	C	C	C	T	G	G	T	T	A	T	C	C	G	T	T	T	T	C	G	C	G	A	C	G	T	G	C	A	A	G	T	T	A
S. filiformis 2134 poly	C	r	M	G	G	A	C	C	C	T	G	G	T	T	A	T	C	C	G	T	T	T	T	y	G	C	G	A	y	G	T	G	C	A	m	G	T	T	A
S. filiformis **2134** major	C	A	A	G	G	A	C	C	C	T	G	G	T	T	A	T	C	C	G	T	T	T	T	C	G	C	G	A	C	G	T	G	C	A	A	G	T	T	A

Species, isolate,	Position in alignment																										no. of poly-	no. of
differential editing, clone	171	177	182	183	184	185	187-194	196	198	203	204	213	214	216	218	219	220	238	239	243	245	250	257	259	261	263	264	morphisms	indels
S. amblyphylla 2602 poly	G	C	C	k	T	s	CTTCTCGA	G	y	C	T	s	G	G	G	A	a/-	T	A	T	G	G	k	A	k	G	C	12	1
S. amblyphylla **2602** major	G	C	C	G	T	G	CTTCTCGA	G	C	C	T	G	G	G	G	A	A	T	A	T	G	G	G	A	G	G	C	0	0
S. amblyphylla 2603 poly	G	m	C	k	T	s	CTTCTCGA	G	y	C	T	s	G	G	G	A	a/-	T	A	T	G	G	k	A	k	G	C	13	1
S. amblyphylla **2603** major	G	C	C	G	T	G	CTTCTCGA	G	C	C	T	G	G	G	G	A	A	T	A	T	G	G	G	A	G	G	C	0	0
S. filiformis 1985 poly	G	C	C	K	T	S	CTTCTCGA	G	y	C	T	G	G	G	G	A	a/-	T	R	W	G	G	G	A	T	G	C	13	1
S. filiformis **1985** major	G	C	C	K	T	S	CTTCTCGA	G	C	C	T	G	G	G	G	A	A	T	R	W	G	G	G	A	T	G	C	5	0
S. filiformis 1987 poly	G	C	C	K	T	S	CTTCTCGA	G	y	C	T	G	G	G	s	A	a/-	T	A	A	G	G	G	A	T	G	C	9	1
S. filiformis **1987** major	G	C	C	K	T	G	CTTCTCGA	G	C	C	T	G	G	G	G	A	A	T	A	A	G	G	G	A	T	G	C	2	0
S. filiformis 2290 poly	G	C	C	k	T	s	CTTCTCGA	G	y	C	T	G	G	G	G	A	a/-	T	r	A	G	G	G	A	T	G	C	9	1
S. filiformis **2290** major	G	C	C	K	T	G	CTTCTCGA	G	C	C	T	G	G	G	G	A	A	T	A	A	G	G	G	A	T	G	C	1	0
S. filiformis 2322 poly	G	C	C	K	T	S	CTTCTCGA	G	C	C	T	G	G	G	s	A	a/-	T	A	A	G	G	G	A	T	G	C	9	1
S. filiformis **2322** major	G	C	C	K	T	G	CTTCTCGA	G	C	C	T	G	G	G	G	A	A	T	A	A	G	G	G	A	T	G	C	2	0
S. filiformis 3192 poly	G	C	C	K	T	S	CTTCTCRA	G	Y	y	T	G	G	G	G	A	a/-	T	A	w	G	G	G	A	k	G	C	19	1
S. filiformis **3192** major	G	C	C	G	T	G	CTTCTCGA	G	C	C	T	G	G	G	G	A	A	T	A	T	G	G	G	A	G	G	C	2	0
S. filiformis 3192 clone 1	G	C	C	G	T	G	CTTCTCGA	G	C	C	T	G	G	G	G	A	A	T	A	A	G	G	G	A	G	G	C	0	0
S. filiformis 3192 clone 2	G	C	C	G	T	G	CTTCTCGA	G	C	C	T	G	G	G	G	A	A	T	A	T	G	G	G	A	G	G	C	0	0
S. filiformis 3192 clone 3	G	C	C	G	T	G	CTTCTCGA	G	C	C	T	G	G	G	G	A	A	T	A	T	G	G	G	A	G	G	C	0	0
S. filiformis 3192 clone 4	G	C	C	T	T	C	CTTCTCGA	G	T	C	T	G	G	G	G	A	-	T	A	T	G	G	G	A	T	G	C	0	0
S. filiformis 3192 clone 5	G	C	C	T	T	C	CTTCTCGA	G	T	C	T	G	G	G	G	A	-	T	A	T	G	G	G	A	T	G	C	0	0
S. filiformis 1060 poly	G	C	C	k	T	S	CTTCTCRA	G	y	C	T	G	G	G	r	A	a/-	w	A	w	G	G	G	A	k	G	C	16	1
S. filiformis **1060** major	G	C	C	T	T	C	CTTCTCGA	G	T	C	T	G	G	G	G	A	-	T	A	T	G	G	G	A	T	G	C	1	0
S. filiformis 1703 poly	G	C	C	K	T	S	CTTCTCGA	G	Y	C	T	G	G	G	G	A	a/-	T	A	W	G	G	G	A	k	s	C	14	1
S. filiformis **1703** major	G	C	C	K	T	G	CTTCTCGA	G	Y	C	T	G	G	G	G	A	A	T	A	W	G	G	G	A	G	C	C	6	0
S. filiformis 2095 poly	G	C	C	G	T	G	cttctcga/--	K	C	C	T	G	G	G	G	A	a/-	T	A	T	K	K	G	A	G	G	A	6	2
S. filiformis **2095** major	G	C	C	G	T	G	--------	G	C	C	T	G	G	G	G	A	A	T	A	T	K	K	G	A	G	G	A	2	0
S. filiformis 2095 clone 1	G	C	C	T	T	C	CTTCTCGA	G	T	C	T	G	G	G	G	A	-	T	A	T	G	G	G	G	G	G	C	0	0
S. filiformis 2095 clone 2	G	C	C	T	T	C	CTTCTCGA	G	T	C	T	G	G	G	G	A	-	T	A	T	G	G	G	A	T	G	C	0	0
S. filiformis 2095 clone 3	G	C	C	T	T	C	CTTCTCGA	G	C	C	T	G	G	G	G	A	A	T	A	T	G	G	G	A	G	G	A	0	0
S. filiformis 2095 clone 4	G	C	C	G	T	G	--------	G	C	C	T	G	G	G	G	A	A	T	A	T	G	G	G	G	G	G	C	0	0
S. filiformis 2095 clone 5	G	C	C	G	T	G	CTTCTCGA	G	C	C	T	G	G	G	G	A	A	T	A	T	G	G	G	A	G	G	A	0	0
S. filiformis 1941 poly	G	C	C	k	T	s	cttctcga/--	G	C	C	T	G	G	G	G	A	A	T	A	T	G	G	G	A	G	G	C	3	1
S. filiformis **1941** major	G	C	C	G	T	G	--------	G	C	C	T	G	G	G	G	A	A	T	A	T	G	G	G	A	G	G	C	0	0
S. filiformis 2440 poly	G	C	C	k	T	s	CTTCTCGA	G	y	C	T	s	G	G	g/-	r	A	T	A	w	G	r	G	A	G	G	C	14	1
S. filiformis **2440** major	G	C	C	G	T	G	CTTCTCGA	G	C	C	T	G	G	G	G	A	A	T	A	T	G	G	G	A	G	G	C	0	0
S. filiformis 2462 poly	G	C	C	G	T	G	cttctcga/--	k	C	C	T	G	G	G	G	A	A	T	A	T	G	G	G	G	G	G	C	2	1
S. filiformis **2462** major	G	C	C	G	T	G	CTTCTCGA	G	C	C	T	G	G	G	G	A	A	T	A	T	G	G	G	G	G	G	C	0	0
S. filiformis 2462 clone 2	G	C	C	G	T	G	CTTCTCGA	G	C	C	T	G	G	G	G	A	A	T	A	T	G	G	G	A	G	G	C	0	0
S. filiformis 2462 clone 3	G	C	C	G	T	G	CTTCTCGA	G	C	C	T	G	G	G	G	A	A	T	A	T	G	G	G	G	G	G	C	0	0
S. filiformis 2462 clone 5	G	C	C	G	T	G	CTTCTCGA	G	C	C	T	G	G	G	G	A	A	T	A	T	G	G	G	G	G	G	C	0	0
S. filiformis 2543 poly	S	C	C	G	T	G	CyTCTCGR	G	C	C	w	G	s	G	G	A	A	T	A	T	G	G	G	A	k	G	C	13	0
S. filiformis **2543** major	S	C	C	G	T	G	CTTCTCGG	G	C	C	T	G	G	G	G	A	A	T	A	T	G	G	G	A	G	G	C	4	0
S. filiformis 1187 poly	S	C	C	G	T	G	CyTCTCGR	G	C	C	w	G	s	G	G	A	A	T	A	T	G	G	G	A	k	G	C	12	0
S. filiformis **1187** major	S	C	C	G	T	G	CTTCTCGG	G	C	C	T	G	G	G	G	A	A	T	A	T	G	G	G	A	G	G	C	4	0
S. filiformis 2794 poly	G	C	C	G	T	G	CTTCTCGA	G	C	C	T	G	G	G	g/-	A	A	T	A	T	G	G	G	A	G	G	C	2	1
S. filiformis **2794** major	G	C	C	G	T	G	CTTCTCGA	G	C	C	T	G	G	G	G	A	A	T	A	T	G	G	G	A	G	G	C	0	0
S. filiformis 2134 poly	G	C	y	k	w	s	CTTCTCGR	G	C	C	w	G	G	k	g/-	A	A	T	A	T	G	G	G	A	G	G	C	12	1
S. filiformis **2134** major	G	C	C	G	T	G	CTTCTCGR	G	C	C	T	G	G	G	G	A	A	T	A	T	G	G	G	A	G	G	C	1	0

Legend: Intraspecific and intra-individual (polymorphisms) variation is summarized; variable positions are shown. Positions in the alignment start with the first base after the forward primer. Colors are for better distinction of the variation. Samples in bold were used for tree construction. poly: all polymorphisms included, major: dominant ribotype (excluding small additional peaks).
Cloned sequences of the same sample are on gray background; clones were submitted to GenBank, but used only to unequivocally determine the position of indels.
69 positions with variation (including variation in longer indel) out of 269 characters = 25.6%
