# Supplementary material for: Evolutionary history and patterns of geographical variation, fertility, and hybridization in Stuckenia (Potamogetonaceae)
Source: Front Plant Sci. 2022 Nov 3;13:1042517. doi: 10.3389/fpls.2022.1042517 (PMC9670304; doi:10.3389/fpls.2022.1042517)
Supplement: Supplementary file 8 [file Table_4.pdf]

Supplementary Table 4 | Intra- and interspecific variation of *Stuckenia* and origin of hybrids (*rpl20–5'rps12*)

Species or hybrid (isolate)	Position in alignment																
	60	140	206-213	219	230	240	243-262	264	271	367	467	584	672	699	700	723	771
S. amblyphylla (**2602**, **2603**)	T	T	ATTTCATC	A	C	A	–	A	A	A	C	G	A	T	A	A	T
S. amblyphylla × *S. filiformis* (**2183**, 2666, **2789**)[1]	T	T	ATTTCATC	A	C	A	–	A	A	A	C	G	A	T	A	A	T
S. filiformis (**1187**, 1941, 2095, **2108**, 2134, 2453, **2462**, 2543)	T	T	ATTTCATC	A	C	A	–	A	A	A	C	G	A	T	A	A	T
S. filiformis (2794)[2]	w	T	ATTTCATC	A	C	A	–	A	A	A	C	G	A	T	A	A	T
S. filiformis × *S. pectinata* (**1009**)	T	T	ATTTCATC	A	C	A	–	A	A	A	C	G	A	T	A	A	T
S. filiformis × *S. amblyphylla* (**3258**)	T	T	ATTTCATC	A	C	A	–	A	A	A	C	G	A	T	A	C	T
S. filiformis (**2440**, **2793**)	T	T	- 8 bp	A	C	A	–	A	C	A	C	G	A	T	A	C	T
S. filiformis (**1060**, **1703**, **3192**)	T	T	ATTTCATC	A	T	A	–	A	A	A	C	T	A	T	A	A	T
S. filiformis × *S. pectinata* (**2327**)	T	T	ATTTCATC	A	T	A	–	A	A	A	C	T	A	T	A	A	T
S. filiformis (**1985**, **1987**, **1989**, 1992, 2006, 2288, 2290, 2291, 2322)	T	T	ATTTCATC	A	T	A	–	A	A	A	C	T	G	T	A	A	T
S. filiformis × *S. vaginata* (**1651**, 1710, 1877, 1878, 1879, 1980, 2652)	T	T	ATTTCATC	A	T	A	–	A	A	A	C	T	G	T	A	A	T
S. filiformis × *S. pectinata* (**1993**, 1995, 1996, 1998, 2002, 2003, 2004, 2010, 2287, 2293, 2294, 2303, 2314, 2321, 3223, 3226)	T	T	ATTTCATC	A	T	A	–	A	A	A	C	T	G	T	A	A	T
S. pamirica (**1753**)	T	T	ATTTCATC	–	C	A	–	A	A	A	C	G	G	T	A	A	C
S. vaginata (**1999**, 2016, **2052**, 2097, 2132)	T	G	ATTTCATC	A	C	A	–	A	A	C	C	G	G	T	A	A	T
S. vaginata × *S. pectinata* (**1027**, 1840, 1868, 1870, 1875, 1978, 2087, 2088, 2136, 2555, 2556, 3075)	T	G	ATTTCATC	A	C	A	–	A	A	C	C	G	G	T	A	A	T
S. vaginata × *S. filiformis* (**1991**, 2141, 2455)	T	G	ATTTCATC	A	C	A	–	A	A	C	C	G	G	T	A	A	T
S. vaginata (**1063**, **1976**)	T	G	ATTTCATC	A	C	A	–	C	A	C	C	G	G	T	A	A	T
S. vaginata × *S. filiformis* (**2446**, 2452)	T	G	ATTTCATC	A	C	A	–	A	A	C	C	G	G	T	C	A	T
S. striata × *S.* sp. (**855**)	T	G	ATTTCATC	A	C	A	–	A	A	C	T	G	G	–	A	A	T
S. striata (**1034**, **2185**)	T	T	ATTTCATC	A	C	A	–	A	A	C	T	G	G	–	C	A	T
S. pectinata (**133**, 981, 989, 1010, 1023, 1650, 1652, 1711, 1837, 1841, 1869, 1935, **2040**, 2051, 2071, 2090, 2116, 2210, 2283, 2448, 2465, 2484, 2538, 2561, **2689**, 2690, 2707, 2726, 2796, 2797, 2920, 3201, 3210)	T	T	ATTTCATC	A	C	A	–	A	A	C	T	G	G	–	C	A	T
S. pectinata × *S. filiformis* (**2168**, 2253)	T	T	ATTTCATC	A	C	A	–	A	A	C	T	G	G	–	C	A	T
S. pectinata × *S. vaginata* (**2466**)	T	T	ATTTCATC	A	C	A	–	A	A	C	T	G	G	–	C	A	T
S. pectinata (**2026**)	T	T	ATTTCATC	A	C	A	+ 20 bp	A	A	C	T	G	G	–	C	A	T
S. pectinata (**2228**)	T	T	ATTTCATC	A	C	C	–	A	A	C	T	G	A	–	C	A	T

[1] These hybrids' maternal parent cannot be distinguished with this marker.

[2] This sample shows a polymorphic position in chloroplast DNA, a rare incidence of heteroplasmy.

Groenlandia densa: three identical sequences, no close relatives

Legend: Intra- and interspecific variation is summarized; variable positions are shown. Positions in the alignment start with the first base after the forward primer. Colors are for better distinction of the variation. Samples in bold cover the variation and were used for tree construction.
