# Supplementary material for: Evolutionary history and patterns of geographical variation, fertility, and hybridization in Stuckenia (Potamogetonaceae)
Source: Front Plant Sci. 2022 Nov 3;13:1042517. doi: 10.3389/fpls.2022.1042517 (PMC9670304; doi:10.3389/fpls.2022.1042517)
Supplement: Supplementary file 9 [file Table_5.pdf]

Supplementary Table 5 | Intra- and interspecific variation of closely related taxa of *Stuckenia* and maternal origins of hybrids (*trnT–trnL*)

A) *Stuckenia pectinata*, *S. striata*

Species (isolate)	Position in alignment													
	**15**	**61**	**72-73**	**138**	**222-223**	**245-266**	**310-314**	**363-364**	**395**	**417-422**	**625**	**756**	**760**	**810**
S. pectinata (**133**, 981, 1023, 1650, 1652, 1837, 1841, 1869, 1935, 2026, **2051**, 2071, 2116, 2210, 2283, 2448, 2465, 2484, 2538, 2561, **2689**, 2690, 2705, 2707, 2796, 2797, 2920, 3201, 3210, 3225)	G	A	TT	T	TC	+ 22 bp	–	CT	C	AATTTT	T	A	T	G
S. striata (**1034**, **2185**)	G	A	TT	T	TC	+ 22 bp	–	CT	C	AATTTT	T	A	T	G
S. pectinata (**2040**)	G	A	TT	T	TC	+ 22 bp	+ 5 bp	CT	C	AATTTT	T	A	T	G
S. pectinata (**2726**)	G	A	TT	T	TC	+ 22 bp	–	CT	C	AATTTT	T	A	G	G
S. striata × *S.* sp. (**855***)	T	G	AC	G	–A	–	–	TG	T	- 6 bp	C	T	T	T

* maternal parent *S.* sp., probably a highly divergent genotype of *S. striata*, see Discussion

B) *Stuckenia pamirica*, *S. amblyphylla*, *S. filiformis*, *S. vaginata*, and their hybrids

Species (isolate)	Position in alignment																
	**99**	**121**	**132**	**145**	**212**	**285-305**	**338-344**	**345-346**	**367**	**393**	**422**	**431**	**458**	**543**	**686**	**780**	**809**
S. pamirica (**1753**)	C	A	T	C	C	–	–	TC	C	C	G	–	C	A	G	G	T
S. amblyphylla (**2602**, **2603**)	C	A	T	C	C	–	–	TC	C	T	T	–	A	A	A	G	T
S. amblyphylla × *S. filiformis* (**2183***, 2666*, 2789*)	C	A	T	C	C	–	–	TC	C	T	T	–	A	A	A	G	T
S. filiformis (**1187**, **1941**, **2543**)	C	A	T	C	C	–	–	TC	C	T	T	T	A	A	A	A	T
S. filiformis (**2095**, **2453**, **2462**)	C	A	T	C	C	–	–	TC	A	T	T	–	A	A	A	G	T
S. filiformis × *S. vaginata* (**2460**)	C	A	T	C	C	–	–	TC	A	T	T	–	A	A	A	G	T
S. filiformis (**1985**, 1987, 1992, **2290**, 2291, 2322)	C	C	T	C	C	–	–	TC	C	T	T	–	A	A	A	G	T
S. filiformis × *S. vaginata* (**2652**)	C	C	T	C	C	–	–	TC	C	T	T	–	A	A	A	G	T
S. filiformis (**1060**, **1703**, **3192**)	C	C	T	C	C	–	–	AA	C	T	T	–	A	A	A	G	T
S. filiformis (**2134**, **2794**)	C	A	T	C	C	–	–	TC	C	T	T	–	A	A	A	G	T
S. filiformis (**2440**, **2793**)	C	A	T	C	C	–	–	TC	C	C	G	–	C	A	A	G	T
S. filiformis × *S. amblyphylla* (**3258**)	C	A	T	C	C	–	–	TC	C	C	G	–	C	A	A	G	T
S. vaginata (**1999**)	C	A	T	T	T	+ 21 bp	+ 7 bp	TC	C	C	T	–	C	A	A	G	G
S. vaginata (**2016**)	C	A	G	T	T	+ 21 bp	+ 7 bp	TC	C	C	T	–	C	A	A	G	G
S. vaginata (**1063**, **1976**)	A	A	T	T	T	+ 21 bp	+ 7 bp	TC	C	C	T	–	C	A	A	G	G
S. vaginata × *S. filiformis* (**2446**, 2452, 2454, 2455)	A	A	T	T	T	–	+ 7 bp	TC	C	C	T	–	A	A	A	G	G
S. vaginata (**2052**, **2097**, **2132**)	A	A	T	T	T	–	+ 7 bp	TC	C	C	T	–	C	A	A	G	G
S. vaginata × *S. filiformis* (**2141**, 2456, 2457, 2459)	A	A	T	T	T	–	+ 7 bp	TC	C	C	T	–	C	A	A	G	G
S. vaginata × *S. filiformis* (2467)	A	A	T	T	T	–	+ 7 bp	TC	C	C	T	–	C	N	N	N	N
S. vaginata × *S. filiformis* (**2458**)	A	A	T	T	T	–	+ 7 bp	TC	C	C	T	–	C	C	A	G	G

* maternal parent equivocal

Groenlandia densa: three identical sequences, no close relatives

Legend: Intra- and interspecific variation is summarized; variable positions are shown. For deletions or insertions, sometimes only the length is given to save space. Positions in the alignment start with the first base after the forward primer and are specific for each table. Colors are for better distinction of the variation. Samples in bold cover the variation and were used for tree construction. Hybrids are included to show their sequences compared to the respective parents.
