# Supplementary material for: Evolutionary history and patterns of geographical variation, fertility, and hybridization in Stuckenia (Potamogetonaceae)
Source: Front Plant Sci. 2022 Nov 3;13:1042517. doi: 10.3389/fpls.2022.1042517 (PMC9670304; doi:10.3389/fpls.2022.1042517)
Supplement: Supplementary file 11 [file Table_7.pdf]

Supplementary Table 7 | Genotypes of *Stuckenia* species, hybrid identification and maternal origins. Additions -x, -y, -a, -b, -c, -d, -e, -f to genotypes 1 and 2 indicate subsets of the genotypes based on *ITS* that were found with the other markers. Genotypes gt-x and gt-y are unique and cannot be attributed to *ITS* genotypes 1 or 2. (!) indicates conflict between markers. * indicates samples with multiple or novel variants of *S. pectinata ITS* genotypes 1 or 2. ~ indicates similarity with other genotypes. Subtypes of *S. vaginata* genotype 2 involved in the hybrid with *S. pectinata* (sample 1870) cannot be distinguished because the end of the sequence is missing. Concerning the 'hybrid' of *S. striata*, see Discussion. For details, see Figures 1–3, 5–6, and Supplementary Tables 2–6. Combinations of genotypes in interspecific hybrids are listed in the same order as the parental species. For details about population origins, see Supplementary Table 1.

**Species / Hybrid**	**Sample**	**Country**	**Genotype (gt) and Similarity**	**Genotype (gt) and Similarity corresponding to *ITS* (all) Maternal Parent of Hybrids (*rpl*20-5'*rps*12, *trn*T-*trn*L)**		
			ITS	***rpl*20-5'*rps*12**	***trn*T-*trn*L**	***5S-NTS***
S. amblyphylla	2602	Tajikistan	unique	*S. filiformis* gt2	*S. filiformis* gt2	*S. filiformis* gt2
	2603	Tajikistan	unique	*S. filiformis* gt2	*S. filiformis* gt2	*S. filiformis* gt2
S. filiformis	1060	USA	gt1 × gt2	gt1-x	gt1-x	gt1-x
	1187	Switzerland	gt2	gt2	gt2-x	gt2-c
	1703	USA	gt1 × gt2	gt1-x	gt1-x	gt2-a
	1941	Montenegro	gt2	gt2	gt2-x	gt2-e
	1985	USA	gt1 × gt2	gt1	gt1	gt1
	1987	USA	gt1	gt1	gt1	gt1
	1989	USA	gt1	gt1		
	1992	USA	gt1	gt1	gt1	
	2006	USA	gt1	gt1		
	2095	Finland	gt2	gt2	gt2-y	gt2-f
	2108	Finland	gt2	gt2		
	2134	Russia	gt2	gt2	gt2	gt2
	2288	USA	gt1	gt1		
	2290	USA	gt1	gt1	gt1	gt1
	2291	USA	gt1	gt1	gt1	
	2296	USA	gt1			
	2297	USA	gt1			
	2298	USA	gt1			
	2322	USA	gt1	gt1	gt1	gt1
	2440	China	gt2	gt-x	gt-x	gt2
	2453	Russia	gt2	gt2	gt2-y	
	2461	Russia	gt2			
	2462	Russia	gt2	gt2	gt2-y	gt2-d
	2463	Russia	gt2			
	2464	Russia	gt2			
	2543	Switzerland	gt2	gt2	gt2-x	gt2-c
	2793	China	gt2	gt-x	gt-x	
	2794	China	gt2	gt2	gt2	gt2
	3192	USA	gt2	gt1(!)	gt1-x(!)	gt2-b
	3216	USA	gt1			
	3217	USA	gt1			
	3218	USA	gt1			
	3229	USA	gt1			
	3248	USA	gt1			
	3252	USA	gt1			
S. pamirica	1753	India	~*S. filiformis*	~*S. filiformis*	~*S. filiformis*	unique
S. pectinata	133	Italy	gt1a	equivocal	equivocal	gt1a
	981	Switzerland	gt1a	equivocal	equivocal	gt1a-e
	989	Switzerland	gt1a	equivocal		
	1010	Sweden	gt1a	equivocal		
	1023	Denmark	gt1a × 1b	equivocal	equivocal	

	1650	USA	gt2b	equivocal	equivocal	
	1652	USA	gt2b	equivocal	equivocal	gt2b
	1711	USA	gt1a × 2b	equivocal		
	1837	Russia	gt2a	equivocal	equivocal	
	1841	Russia	gt2a	equivocal	equivocal	gt2a
	1869	USA	gt2a × 2b	equivocal	equivocal	gt2b
	1935	Hungary	gt1a	equivocal	equivocal	gt1a
	2026	India	gt1a	gt-x	equivocal	gt1a-a
	2040	Malawi	gt1b	equivocal	gt-x	gt1b
	2051	Russia	gt2a	equivocal	equivocal	gt2a
	2071	Czech Rep.	gt1a	equivocal	equivocal	gt1a
	2090	Finland	gt1a × 2a	equivocal		
	2116	Finland	gt1	equivocal	equivocal	gt1a-c
	2210	Switzerland	gt1a × 1b	equivocal	equivocal	gt1a-d
	2211	Switzerland	gt1a × 1b			
	2212	Switzerland	gt1a × 1b			
	2213	Switzerland	gt1a × 1b			
	2228	USA	gt1b × 2e	gt-y		
	2283	USA	gt2c × 2d	equivocal	equivocal	gt2b(!)
	2448	Russia	gt1a	equivocal	equivocal	gt1a
	2465	Russia	gt1a	equivocal	equivocal	gt1a-x
	2484	Russia	gt2a	equivocal	equivocal	gt2a-x
	2485	Russia	gt2a			
	2486	Russia	gt2a			
	2487	Russia	gt2a			
	2488	Russia	gt2a			
	2538	Slovakia	gt1a × 1b	equivocal	equivocal	gt1a-y
	2541	Slovakia	gt1a			
	2545	Switzerland	gt1a			
	2561	Denmark	gt1a	equivocal	equivocal	gt1a
	2586	Denmark	gt1a × 1b			
	2587	Denmark	gt1a × 1b			
	2589	Denmark	gt1a × 1b			
	2644	USA	gt2b			
	2689	India	gt1a × 1b	equivocal	equivocal	gt1a-e
	2690	India	gt1b × 2a	equivocal	equivocal	gt1a-f(!)
	2694	Kazakhstan	gt1a			
	2695	Russia	gt1a			
	2696	Russia	gt1a			
	2697	Russia	gt1a			
	2698	Russia	gt1a			
	2699	Russia	gt1a			
	2705	Russia	gt1a		equivocal	gt1a-y
	2706	Russia	gt1a			
	2707	Russia	gt1a	equivocal	equivocal	gt1a-y
	2708	Russia	gt1a			
	2724	Russia	gt1a			
	2725	Russia	gt1a			
	2726	Russia	gt1a	equivocal	gt-y	gt1a-b
	2795	China	gt1a			
	2796	China	gt1a × 2a	equivocal	equivocal	
	2797	China	gt2a	equivocal	equivocal	gt1a × 2a
	2920	USA	gt2b	equivocal	equivocal	gt2b-x
	3201	Turkey	gt1a	equivocal	equivocal	gt1a-f
	3210	USA	gt2c × 2d	equivocal	equivocal	gt2b(!)
	3225	USA	gt2c × 2b	equivocal	equivocal	gt2b
S. striata	1034	Argentina	~*S. pectinata* gt2	*S. pectinata*	*S. pectinata*	~*S. pectinata* gt2

	2185	Bolivia	~*S. pectinata* gt2	*S. pectinata*	*S. pectinata*	
	3029	Peru	~*S. pectinata* gt2			
S. vaginata	1063	Canada	gt2a	gt-x	gt2a	gt2
	1919	Finland	gt1			
	1976	USA	gt2a	gt-x	gt2a	gt2
	1999	USA	gt2a	equivocal	gt2a-x	gt2
	2016	USA	gt2b	equivocal	gt2b	gt2
	2052	Russia	gt1	equivocal	gt1	gt1
	2097	Finland	gt1	equivocal	gt1	gt1
	2132	Russia	gt1	equivocal	gt1	gt1
S. amblyphylla × *S. filiformis*	2183	India	*S.a.* × *S.f.* gt2	equivocal	equivocal	
	2666	India	*S.a.* × *S.f.* gt2	equivocal	equivocal	
	2789	Kazakhstan	*S.a.* × *S.f.* gt2	equivocal	equivocal	
	3258	India	*S.a.* × *S.f.* gt2	equivocal	*S. filiformis* gt-x	
S. pectinata × *S. filiformis*	1009	Sweden	gt1a × gt2	*S. filiformis*		
	2168	Germany	gt1a × gt2	*S. pectinata*		
	2253	Germany	gt1a × gt2	*S. pectinata*		
	1993	USA	gt2* × gt1	*S. filiformis*		
	1995	USA	gt2b × gt1	*S. filiformis*		
	1996	USA	gt2b × gt1	*S. filiformis*		
	1998	USA	gt2b × gt1	*S. filiformis*		
	2002	USA	gt2c × gt1	*S. filiformis*		
	2003	USA	gt2d × gt1	*S. filiformis*		
	2004	USA	gt2* × gt1	*S. filiformis*		
	2010	USA	gt2b × gt1	*S. filiformis*		
	2287	USA	gt2c × gt1	*S. filiformis*		
	2293	USA	gt2c × gt1	*S. filiformis*		
	2294	USA	gt2c × gt1	*S. filiformis*		
	2303	USA	gt2c × gt1	*S. filiformis*		
	2314	USA	gt2* × gt1	*S. filiformis*		
	2321	USA	gt2b × gt1	*S. filiformis*		
	2327	USA	gt2b × gt1	*S. filiformis*		
	3223	USA	gt2b × gt1	*S. filiformis*		
	3226	USA	gt2b × gt1	*S. filiformis*		
S. filiformis × *S. vaginata*	2141	Russia	gt1 × gt1	*S. vaginata*	*S. vaginata*	
	2454	Russia	gt1 × gt1-x		*S. vaginata*	
	2455	Russia	gt1 × gt1-x	*S. vaginata*	*S. vaginata*	
	1651	USA	gt2 × gt2a-x	*S. filiformis*		
	1710	USA	gt2 × gt2a-x	*S. filiformis*		
	1877	USA	gt2 × gt2a	*S. filiformis*		
	1878	USA	gt1 × gt2a	*S. filiformis*		
	1879	USA	gt1 × gt2a	*S. filiformis*		
	1980	USA	gt1 × gt2a	*S. filiformis*		
	1991	USA	gt1 × gt2a	*S. vaginata*		
	2652	Canada	gt1 × gt2a	*S. filiformis*	*S. filiformis*	
	2446	Russia	gt1 × gt2a-y	*S. vaginata* gt-x	*S. vaginata*	
	2452	Russia	gt1 × gt2a-y	*S. vaginata* gt-x	*S. vaginata*	
	2456	Russia	gt2 × gt1		*S. vaginata*	
	2457	Russia	gt2 × gt1		*S. vaginata*	
	2458	Russia	gt2 × gt1		*S. vaginata* gt-x	
	2459	Russia	gt2 × gt1		*S. vaginata*	
	2460	Russia	gt2 × gt1		*S. filiformis*	
	2467	Russia	gt2 × gt1		*S. vaginata*	
S. pectinata × *S. vaginata*	1027	Denmark	gt1a × gt1	*S. vaginata*		

	1840	Russia	gt1a* × gt1	*S. vaginata*		
	2087	Finland	gt1a × gt1	*S. vaginata*		
	2088	Finland	gt1a* × gt1	*S. vaginata*		
	2136	Russia	gt1a × gt1	*S. vaginata*		
	2466	Russia	gt1a* × gt1	*S. pectinata*		
	2555	Denmark	gt1a × gt1	*S. vaginata*		
	2556	Denmark	gt1a × gt1	*S. vaginata*		
	1868	USA	gt2b × gt2a	*S. vaginata*		
	1870	USA	gt2b × gt2	*S. vaginata*		
	1875	USA	gt2b × gt2a	*S. vaginata*		
	1978	USA	gt2b × gt2a	*S. vaginata*		
	3075	USA	gt2b × gt2a	*S. vaginata*		
S. striata × *S.* sp.	855	Argentina	*S. striata* × *S.* sp.	*S.* sp.	*S.* sp.	*S. striata*, *S.* sp.
