# Supplementary material for: Evolutionary history and patterns of geographical variation, fertility, and hybridization in Stuckenia (Potamogetonaceae)
Source: Front Plant Sci. 2022 Nov 3;13:1042517. doi: 10.3389/fpls.2022.1042517 (PMC9670304; doi:10.3389/fpls.2022.1042517)
Supplement: Supplementary file 10 [file Table_6.pdf]

Supplementary Table 6 | Hybrid identification based on *ITS*.

Stuckenia Species / Hybrid	Position in Alignment																																					
S. pectinata* × *S. filiformis	**4**	**5**	**18-19**	**28**	**29**	**37**	**60**	**63**	**64**	**84**	**85**	**98**	**100**	**102**	**116**	**117-118**	**130**	**163**	**215**	**237**	**446-447**	**452**	**477**	**484**	**516**	**538**	**547**	**563**	**600**	**601**	**603**	**615**	**616**	**629**	**630-638**	**643**	**644**	**656**	**659**
***S. pectinata* 2283 c4 gt2c**	C	G	GA	T	T	A	C	C	G	G	C	G	T	C	-	TG	T	A	G	C	-	T	C	C	G	G	A	A	T	C	G	A	A	G	-	G	C	A	T
pect gt2c × fili gt1 2287 a1	C	G	GA	T	T	A	C	C	G	G	C	G	T	C	-	TG	T	A	G	C	-	T	C	C	G	G	A	A	T	C	G	A	A	G	-	G	C	A	T
pect gt2c × fili gt1 2303 c1	C	G	GA	T	T	A	C	C	G	G	C	G	T	C	-	TG	T	A	G	C	-	T	C	C	G	G	A	A	T	C	G	A	A	G	-	G	C	A	T
pect gt2c × fili gt1 2294 M	C	G	GA	T	T	A	C	C	G	G	C	G	T	C	-	TG	T	A	G	C	-	T	C	C	G	G	A	A	T	C	G	A	A	G	-	G	C	A	T
pect gt2c × fili gt1 2293 c2	C	G	GA	T	T	A	C	C	G	G	C	G	T	C	-	TG	T	A	G	C	-	T	C	C	G	G	A	A	T	C	G	A	A	G	-	G	C	A	T
pect gt2c × fili gt1 2002 c4	C	G	GA	T	T	A	C	C	G	G	C	G	T	C	-	TG	T	A	G	C	-	T	C	C	G	G	A	A	T	C	G	A	A	G	-	G	C	A	T
pect gt2* x fili gt1 2004 c5	C	G	GA	A	C	A	C	C	G	G	C	G	T	C	-	-	T	A	G	C	-	T	C	C	G	G	A	A	T	C	G	A	A	G	-	G	C	A	T
pect gt2* x fili gt1 2004 c8	C	G	GA	A	C	A	C	C	G	G	C	G	T	C	-	TG	T	A	G	C	-	T	C	C	G	G	A	A	T	C	G	A	A	G	-	G	C	A	T
pect gt2* x fili gt1 2004 c2	C	G	GA	T	T	A	C	C	G	G	C	G	T	C	-	TG	T	A	G	C	-	T	C	C	G	G	A	A	T	C	G	A	A	G	-	G	C	A	T
pect gt2* x fili gt1 2004 c6	C	G	GA	T	T	A	C	C	G	G	C	G	T	C	-	TG	T	A	G	C	-	T	C	C	G	G	A	A	T	C	G	A	A	G	-	T	T	A	T
***S. pectinata* 2228 m gt2e**	C	G	GA	T	T	A	C	C	G	G	C	G	T	C	-	-	T	A	G	C	AA	T	C	C	G	G	A	A	T	C	G	A	A	G	-				
***S. pectinata* 3210 c9 gt2d**	C	G	GA	A	C	A	C	C	G	G	C	G	T	C	-	-	T	A	G	C	AA	T	C	C	G	G	A	A	T	C	G	A	A	G	-	T	T	A	T
pect gt2d × fili gt1 2003 m	C	G	GA	A	C	A	C	C	G	G	C	G	T	C	-	-	T	A	G																				
***S. pectinata* 1841 gt2a**	C	G	GA	A	C	A	C	C	G	G	C	G	T	C	-	TG	T	A	G	C	AA	T	C	C	G	G	A	A	T	C	G	A	A	G	-	G	C	A	T
***S. pectinata* 1650 gt2b**	C	G	GA	A	C	A	C	C	G	G	C	G	T	C	-	-	T	A	G	C	AA	T	C	C	G	G	A	A	T	C	G	A	A	G	-	G	C	A	T
pect gt2b × fili gt1 2010 M	C	G	GA	A	C	A	C	C	G	G	C	G	T	C	-	-	T	A	G	C	AA	T	C	C	G	G	A	A	T	C	G	A	A	G	-	G	C	A	T
pect gt2* × fili gt1 1993 c9	C	G	GA	A	C	A	C	C	G	G	C	G	T	C	-	-	T	A	G	C	AA	T	C	C	A	G	A	A	T	C	G	A	A	G	-	G	C	A	T
pect gt2* × fili gt1 2314 c2	C	G	GA	A	C	A	C	C	G	G	C	G	T	C	-	-	T	A	G	C	AA	T	C	C	A	G	A	A	T	C	G	A	A	G	-	G	C	A	T
pect gt2* × fili gt1 2314 c5	C	G	-	A	C	A	C	C	G	G	C	G	T	C	-	-	T	A	G	C	AA	T	C	C	G	G	A	A	T	C	G	A	A	G	-	G	C	A	T
pect gt2b × fili gt1 2321 M	C	G	GA	A	C	A	C	C	G	G	C	G	T	C	-	-	T	A	G	C	AA	T	C	C	G	G	A	A	T	C	G	A	A	G	-	G	C	A	T
pect gt2b × fili gt1 2327 a1	C	G	GA	A	C	A	C	C	G	R	C	G	T	C	-	-	T	A	G	C	AA	T	C	C	G	G	A												
pect gt2b × fili gt1 3223 c5	C	G	GA	A	C	A	C	C	G	G	C	G	T	C	-	-	T	A	G	C	AA	T	C	C	G	G	A	A	T	C	G	A	A	G	-	G	C	A	T
pect gt2b × fili gt1 3226 a1	C	G	GA	A	C	A	C	C	G	G	C	G	T	C	-	-	T	A	G	C	AA	T	C	C	G	G	A	A											
pect gt2b × fili gt1 1995 a1	C	G	GA	A	C	A	C	C	G	G	C	G	T	C	-	-	T	A	G	C	AA	T	C	C	G	G	A	A	T	C	G	A	A	G	-	G	C	A	T
***S. pectinata* 1010 gt1a**	C	G	GA	A	C	A	G	C	C	G	C	G	T	C	-	TG	T	A	G	T	AA	T	C	C	G	A	A	A	A	A	C	A	A	G	-	G	C	A	T
pect gt1a × fili gt2 2253 c4	C	G	GA	A	C	A	G	C	C	G	C	G	T	C	-	TG	T	A	G	T	AA	T	C	C	G	A	A	A	A	A	C	A	A	G	-	G	C	A	T
***S. pectinata* 2040 gt1b**	C	G	GA	A	C	A	G	C	C	G	C	G	T	C	-	TG	T	A	G	C	AA	T	C	C	G	A	A	A	A	A	C	A	A	G	-	G	C	A	T
pect gt1a × fili gt2 1009 d	M	G	GA	A	C	W	S	Y	S	K	Y	S	W	S	g/-	TG	Y	W	R	Y	AA	K	Y	c/-	G	R	M	W	W	M	S	W	M	R	9 bp/-	G	C	W	K
pect gt1a × fili gt2 2168 d	M	G	GA	A	C	W	S	Y	S	K	Y	S	W	S	g/-	TG	Y	W	R	Y	AA	K	Y	c/-	G	R	M	W	W	M	S	W	M						
pect gt2b × fili gt1 1996 d	M	R	GA	A	C	W	C	Y	G	K	Y	S	W	S	g/-	tg/-																							
pect gt2b × fili gt1 1998 d	M	R	GA	A	C	W	C	Y	G	K	Y	S	W	S	g/-	tg/-																							
***S. filiformis* 2006 gt1**	A	A	GA	A	C	T	C	T	G	T	T	C	A	G	G	TG	C	T	A	C	AA	G	T	-	G	G	C	T	T	C	G	T	C	A	9 bp	G	C	T	G
pect gt2* × fili gt1 1993 c1	A	A	GA	A	C	T	C	T	G	T	T	C	A	G	G	TG	C	T	A	C	AA	G	T	-	G	G	C	T	T	C	G	T	C	A	9 bp	G	C	T	G
pect gt2b × fili gt1 2327 a2	A	A	GA	A	C	T	C	T	G	T	T	C	A	G	G	TG	C	T	A	C	AA	G	T	-	G	G	C	T	T	C	G	T	C	A	9 bp	G	C		
pect gt2b × fili gt1 3226 a2	A	A	GA	A	C	T	C	T	G	T	T	C	A	G	G	TG	C	T	A	C	AA	G	T	-	G	G	C	T											
pect gt2b × fili gt1 1995 a2	A	A	GA	A	C	T	C	T	G	T	T	C	A	G	G	TG	C	T	A	C	AA	G	T	-	G	G	C	T	T	C	G	T	C	A	9 bp	G	C	T	G
pect gt2c × fili gt1 2002 c5	A	A	GA	A	C	T	C	T	G	T	T	C	A	G	G	TG	C	T	A	C	AA	G	T	-	G	G	C	T	T	C	G	T	C	A	9 bp	G	C	T	G
pect gt2* × fili gt1 2004 c11	A	A	GA	A	C	T	C	T	G	T	T	C	A	G	G	TG	C	T	A	C	AA	G	T	-	G	G	C	T	T	C	G	T	C	A	9 bp	G	C	T	G
pect gt2c × fili gt1 2293 a2	A	A	GA	A	C	T	C	T	G	T	T	C	A	G	G	TG	C	T	A	C	AA	G	T	-	G	G	C	T	T	C	G	T	C	A	9 bp	G	C	T	G
pect gt2c × fili gt1 2294 m	A	A	GA	A	C	T	C	T	G	T	T	C	A	G	G	TG	C	T	A	C	AA	G	T	-															
pect gt2* × fili gt1 2314 c10	A	A	GA	A	C	T	C	T	G	T	T	C	A	G	G	TG	C	T	A	C	AA	G	T	-	G	G	C	T	T	C	G	T	C	A	9 bp	G	C	T	G
pect gt2b × fili gt1 2321 m	A	A	GA	A	C	T	C	T	G	T	T	C	A	G	G	TG	C	T	A	C	AA	G	T	-	G	G	C	T	T	C	G	T	C	A	9 bp	G	C		
pect gt2d × fili gt1 2003 M	A	A	GA	A	C	T	C	T	G	T	T	C	A	G	G	TG	C	T	A	C				-															
pect gt2b × fili gt1 2010 m	A	A	GA	A	C	T	C	T	G	T	T	C	A	G	G	TG	C	T	A	C	AA	G		-															
pect gt2c × fili gt1 2287 a2	A	A	GA	A	C	T	C	T	G	T	T	C	A	G	G	TG	C	T	A	C	AA	G	T	-															
pect gt2c × fili gt1 2303 c2	A	A	GA	A	C	T	C	T	G	T	T	C	A	G	G	TG	C	T	A	C	AA	G	T	-	G	G	C	T	T	C	G	T	C	A	9 bp	G	C	T	G
pect gt2b × fili gt1 3223 c10	A	A	GA	A	C	T	C	T	G	T	T	C	A	G	G	TG	C	T	A	C	AA	G	T	-	G	G	C	T	T	C	G	T	C	A	9 bp	G	C	T	G
***S. filiformis* 1187 gt2**	A	G	GA	A	C	T	C	T	G	T	T	C	A	G	G	TG	C	T	A	C	AA	G	T	-	G	G	C	T	T	C	G	T	C	A	9 bp	G	C	T	G
pect gt1a × fili gt2 2253 c2	A	G	GA	A	C	T	C	T	G	T	T	C	A	G	G	TG	C	T	A	C	AA	G	T	-	G	G	C	T	T	C	G	T	C	A	9 bp	G	C	T	G

Reference sequences of the parental species (see Fig. 1) are given in bold. gt: genotype, c: clone, a: allele, M: major ribotype, m: minor ribotype,
*: genotype variant occurring only in hybrids, -: deletion, empty cells: incomplete end of sequence.

Stuckenia Species / Hybrid	Position in Alignment																																							
S. pectinata* × *S. vaginata	**4**	**28**	**29**	**37**	**60**	**63**	**64**	**69**	**84**	**85**	**92**	**93**	**98**	**100**	**102**	**116**	**117-118**	**130**	**163**	**215**	**237**	**446-447**	**452**	**477**	**484**	**538**	**547**	**563**	**564**	**572**	**600**	**601**	**603**	**615**	**616**	**629**	**630-638**	**643**	**644**	**656**	**659**
***S. pectinata* 2283 c4 gt2c**	C	T	T	A	C	C	G	A	G	C	G	C	G	T	C	-	TG	T	A	G	C	-	T	C	C	G	A	A	T	G	T	C	G	A	A	G	-	G	C	A	T
***S. pectinata* 2228 m gt2e**	C	T	T	A	C	C	G	A	G	C	G	C	G	T	C	-	-	T	A	G	C	AA	T	C	C	G	A	A	T	G	T	C	G	A	A	G					
***S. pectinata* 1841 gt2a**	C	A	C	A	C	C	G	A	G	C	G	C	G	T	C	-	TG	T	A	G	C	AA	T	C	C	G	A	A	T	G	T	C	G	A	A	G	-	G	C	A	T
***S. pectinata* 1650 gt2b**	C	A	C	A	C	C	G	A	G	C	G	C	G	T	C	-	-	T	A	G	C	AA	T	C	C	G	A	A	T	G	T	C	G	A	A	G	-	G	C	A	T
pect gt2b × vag gt2a 1868 M	C	A	C	A	C	C	G	A	G	C	G	C	G	T	C	-	-	T	A	G	C	AA	T	C	C	G	A	A	T	G	T	C	G	A	A	G	-	G	C	A	T
pect gt2b × vag gt2 1870 c3	C	A	C	A	C	C	G	A	G	C	G	C	G	T	C	-	-	T	A	G	C	AA	T	C	C	G	A	A	T	G	T	C	G	A	A	G	-	G	C	A	T
pect gt2b × vag gt2a 1875 M	C	A	C	A	C	C	G	A	G	C	G	C	G	T	C	-	-	T	A	G	C	AA	T	C	C	G	A	A	T	G	T	C	G	A	A	G	-	G	C	A	T
pect gt2b × vag gt2a 1978 M	C	A	C	A	C	C	G	A	G	C	G	C	G	T	C	-	-	T	A	G	C	AA	T	C	C	G	A	A	T	G	T	C	G	A	A	G	-	G	C	A	T
pect gt2b × vag gt2a 3075 M	C	A	C	A	C	C	G	A	G	C	G	C	G	T	C	-	-	T	A	G	C	AA	T	C	C	G	A	A	T	G	T	C	G	A	A	G	-	G	C	A	T
***S. pectinata* 3210 c9 gt2d**	C	A	C	A	C	C	G	A	G	C	G	C	G	T	C	-	-	T	A	G	C	AA	T	C	C	G	A	A	T	G	T	C	G	A	A	G	-	T	T	A	T
***S. pectinata* 2040 gt1b**	C	A	C	A	G	C	C	A	G	C	G	C	G	T	C	-	TG	T	A	G	C	AA	T	C	C	A	A	A	T	G	A	A	C	A	A	G	-	G	C	A	T
***S. pectinata* 1010 gt1a**	C	A	C	A	G	C	C	A	G	C	G	C	G	T	C	-	TG	T	A	G	T	AA	T	C	C	A	A	A	T	G	A	A	C	A	A	G	-	G	C	A	T
pect gt1a* × vag gt1 2088 c3	C	A	C	A	G	C	C	A	G	C	T	A	G	T	C	-	TG	T	A	G	T	AA	T	C	C	A	A	A	T	G	A	A	C	A	A	G	-	G	C	A	T
pect gt1a* × vag gt1 1840 a2	C	A	C	A	G	C	C	A	G	C	T	A	G	T	C	-	TG	T	A	G	T	AA	T	C	C	A	A	A	T	G	A	A	C	A	A	G	-	G	C	A	T
pect gt1a* × vag gt1 2466 a1	C	A	C	A	G	C	C	A	G	C	T	A	G	T	C	-	TG	T	A	G	T	AA	T	C	C	A	A	A	T	G	A	A	C	A	A						
pect gt1a × vag gt1 1027 a1	C	A	C	A	G	C	C	A	G	C	K	M	G	T	C	-	TG	T	A	G	Y	AA	T	C	C	A	A	A	T	G	A	A	C	A	A						
pect gt1a p × vag gt1 2087 a1	C	A	C	A	G	C	C	A	G	C	K	M	G	T	C	-	TG	T	A	G	Y	AA	T	C	C	A	A	A	T	G	A	A	C	A	A						
pect gt1a p × vag gt1 2136 a1	C	A	C	A	G	C	C	A	G	C	K	M	G	T	C	-	TG	T	A	G	Y	AA	T	C	C	A	A	A	T	G	A	A	C	A	A	G	-	G	C	A	T
pect gt1a p × vag gt1 2555 a1	C	A	C	A	G	C	C	A	G	C	K	M	G	T	C	-	TG	T	A	G	Y	AA	T	C	C	A	A	A	T	G	A	A	C	A	A						
pect gt1a p × vag gt1 2556 d	M	A	C	W	S	Y	S	A	K	Y	K	M	S	W	S	g/-	TG	Y	W	R	Y	AA	K	Y	c/-	R	M	W	T	K	W	M	S	W	M						
***S. vaginata* 1919 gt1**	A	A	C	T	C	T	G	A	T	T	G	C	C	A	G	G	TG	C	T	A	C	AA	G	T	-	G	C	T	T	T	T	C	G	T	C	A	9 bp	G	C	T	G
pect gt1a* × vag gt1 2088 c1	A	A	C	T	C	T	G	A	T	T	G	C	C	A	G	G	TG	C	T	A	C	AA	G	T	-	G	C	T	T	T	T	C	G	T	C	A	9 bp	G	C	T	G
pect gt1a p. × vag gt1 2555 a2	A	A	C	T	C	T	G	A	T	T	G	C	C	A	G	G	TG	C	T	A	C	AA	G	T	-	G	C	T	T	T	T	C	G	T	C						
pect gt1a p. × vag gt1 2136 a2	A	A	C	T	C	T	G	A	T	T	G	C	C	A	G	G	TG	C	T	A	C	AA	G	T	-	G	C	T	T	T	T	C	G	T	C	A	9 bp	G	C	T	G
pect gt1a p. × vag gt1 1027 a2	A	A	C	T	C	T	G	A	T	T	G	C	C	A	G	G	TG	C	T	A	C	AA	G	T	-	G	C	T	T	T	T	C	G	T	C						
pect gt1a* × vag gt1 1840 a1	A	A	C	T	C	T	G	A	T	T	G	C	C	A	G	G	TG	C	T	A	C	AA	G	T	-	G	C	T	T	T	T	C	G	T	C	A	9 bp	G	C		
pect gt1a* × vag gt1 2466 a2	A	A	C	T	C	T	G	A	T	T	G	C	C	A	G	G	TG	C	T	A	C	AA	G	T	-	G	C	T	T	T	T	C	G	T	C						
pect gt1a p. × vag gt1 2087 a2	A	A	C	T	C	T	G	A	T	T	G	C	C	A	G	G	TG	C	T	A	C	AA	G	T	-	G	C	T	T	T	T	C	G	T	C						
***S. vaginata* 1063 gt2a**	A	A	C	T	C	T	G	G	T	T	G	C	C	A	G	G	TG	C	T	A	C	AA	G	T	-	G	C	T	T	G	T	C	G	T	C	A	9 bp	G	C	T	G
pect gt2b × vag gt2a 3075 m	A	A	C	T	C	T	G	G	T	T	G	C	C	A	G	G	TG	C	T	A	C	AA	G	T	-	G	C	T	T	G	T	C	G	T	C	A	9 bp	G	C		
pect gt2b × vag gt2a 1978 m	A	A	C	T	C	T	G	G	T	T	G	C	C	A	G	G	TG	C	T	A	C	AA	G	T	-	G	C	T	T	G	T	C	G	T	C	A	9 bp	G	C	T	G
pect gt2b × vag gt2a 1875 m	A	A	C	T	C	T	G	G	T	T	G	C	C	A	G	G	TG	C	T	A	C	AA	G	T	-	G	C	T	T	G	T	C	G	T	C	A	9 bp	G	C	T	G
pect gt2b × vag gt2a 1868 m	A	A	C	T	C	T	G	G	T	T	G	C	C	A	G	G	TG	C	T	A	C	AA	G	T	-	G	C	T	T	G	T	C	G	T	C	A	9 bp	G	C	T	G
pect gt2b × vag gt2 1870 a2	A	A	C	T	C	T	G	G	T	T	G	C	C	A	G	G	TG	C	T	A	C	AA	G																		
***S. vaginata* 2016 gt2b**	A	A	C	T	C	T	G	G	T	T	G	C	C	A	G	G	TG	C	T	A	C	AA	G	T	-	G	C	T	A	G	T	C	G	T	C	A	9 bp	G	C	T	G

Reference sequences of the parental species (see Figure 1) are given in bold. gt: genotype, c: clone, a: allele, M: major ribotype, m: minor ribotype,
*: genotype variant occurring only in hybrids, p: genotype with polymorphic positions, -: deletion, empty cells: incomplete end of sequence.

Stuckenia Species / Hybrid	Position in Alignment														
***S. amblyphylla* × *S. filiformis*<br>*S. filiformis* × *S. vaginata*<br>*S. striata* × *S.* sp.**	**5**	**69**	**162**	**177**	**222**	**439**	**440**	**461**	**519**	**563**	**566**	**571**	**607**	**615**	**642**
***S. amblyphylla* 2602**	G	A	A	T	T	A	A	G	C	T	C	G	C	A	G
***S. amblyphylla* 2603**	G	A	A	T	T	A	A	G	C	T	C	G	C	A	G
amb × fili gt2 2183	G	A	A	T	T	M	A	G	Y	T	C	G	C	M	G
amb × fili gt2 2666	G	A	A	T	T	M	A	G	Y	T	C	G	C	M	G
amb × fili gt2 2789	G	A	A	T	T	M	A	G	Y	T	C	G	C	M	G
amb × fili gt2 3258	G	A	A	T	T	M	A	G	Y	T	C	G	C	M	G
***S. filiformis* 1187 gt2**	G	A	A	T	T	C	A	G	T	T	C	G	C	C	G
***S. filiformis* 2006 gt1**	A	A	A	T	T	C	A	G	T	T	C	G	C	C	G
fili gt1 × vag gt2a 1878	R	R	A	T	T	C	A	G	T	T	C	G	C	C	G
fili gt1 × vag gt2a 1879	R	R	A	T	T	C	A	G	T	T	C	G	C	C	G
fili gt1 × vag gt2a 1980	R	R	A	T	T	C	A	G	T	T	C	G	C	C	G
fili gt1 × vag gt2a 1991	R	R	A	T	T	C	A	G	T	T	C	G	C	C	G
fili gt1 × vag gt2a 2652	R	R	A	T	T	C	A	G	T	T	C	G	C	C	G
fili gt1 × vag gt2-y 2446	R	A	A	Y	T	C	A	G	T	T	S	G	C	C	G
fili gt1 × vag gt2-y 2452	R	A	A	Y	T	C	A	G	T	T	S	G	C	C	G
fili gt1 × vag gt1-x 2454	R	A	A	Y	T	C	A	G	T	T	S	K	C	C	G
fili gt1 × vag gt1-x 2455	R	A	A	Y	T	C	A	G	T	T	S	K	C	C	G
fili gt1 × vag gt1 2141	R	A	A	T	T	C	A	G	T	T	C	K	C	C	G
***S. vaginata* 1919 gt1**	G	A	A	T	T	C	A	G	T	T	C	T	C	C	G
fili gt2 × vag gt1 2456	G	A	A	T	T	C	A	G	T	T	C	K	C	C	G
fili gt2 × vag gt1 2457	G	A	A	T	T	C	A	G	T	T	C	K	C	C	G
fili gt2 × vag gt1 2458	G	A	A	T	T	C	A	G	T	T	C	K	C	C	G
fili gt2 × vag gt1 2459	G	A	A	T	T	C	A	G	T	T	C	K	C	C	G
fili gt2 × vag gt1 2460	G	A	A	T	T	C	A	G	T	T	C	K	C	C	G
fili gt2 × vag gt1 2467	G	A	A	T	T	C	A	G	T	T	C	K	C	C	G
fili gt2 × vag gt2a-x 1651	G	g/-	A	T	T	C	A	G	T	T	C	G	C	C	G
fili gt2 × vag gt2a-x 1710	G	g/-	A	T	T	C	A	G	T	T	C	G	C	C	G
fili gt2 × vag gt2a 1877	G	R	A	T	T	C	A	G	T	T	C	G	C	C	G
***S. vaginata* 1063 gt 2a**	G	G	A	T	T	C	A	G	T	T	C	G	C	C	G
***S. vaginata* 2016 gt 2b**	G	G	A	T	T	C	A	G	T	A	C	G	C	C	G
S. striata × *S.* sp. 855 c9	A	G	G	T	C	C	G	T	T	T	C	G	T	C	C

Reference sequences of the parental species (see Figure 1) are in bold.
gt: genotype, c: clone
